# The chloroplast genome of *Farsetia hamiltonii* Royle*,* phylogenetic analysis, and comparative study with other members of Clade C of Brassicaceae

**DOI:** 10.1186/s12870-022-03750-2

**Published:** 2022-08-02

**Authors:** Nida Javaid, Musarrat Ramzan, Ishtiaq Ahmad Khan, Tahani Awad Alahmadi, Rahul Datta, Shah Fahad, Subhan Danish

**Affiliations:** 1 Department of Botany, The Islamia University, Bahawalpur, Pakistan; 2 Jamil-ur-Rahman Center for Genome Research, Dr. Panjwani Center for Molecular Medicine and Drug Research, International Center for Chemical and Biological Sciences University of Karachi, Karachi, 75270 Pakistan; 3 Department of Pediatrics, College of Medicine and King Khalid University Hospital, King Saud University, Medical City, PO Box-2925, Riyadh, 11461 Saudi Arabia; 4 Department of Geology and Pedology, Faculty of Forestry and Wood Technology, Mendel University in Brno, Zemedelska1, 61300 Brno, Czech Republic; 5 Department of Agronomy, The University of Haripur, Khyber Pakhtunkhwa, Haripur, 22620 Pakistan; 6 Hainan Key Laboratory for Sustainable Utilization of Tropical Bioresource, College of Tropical Crops, Hainan University, Haikou, 570228 China; 7 Department of Soil Science, Faculty of Agricultural Sciences and Technology, Bahauddin Zakariya University, Multan, 60800 Punjab Pakistan

**Keywords:** Farsetia hamiltonii, Farsetiaoccidentalis, Monophyletic, Polymorphic regions, Synonymous substitutions, Brassicaceae

## Abstract

**Background:**

*Farsetia hamiltonii* Royle is a medicinally important annual plant from the Cholistan desert that belongs to the tribe Anastaticeae and clade C of the Brassicaceae family. We provide the entire chloroplast sequence of *F.hamiltonii*, obtained using the Illumina HiSeq2500 and paired-end sequencing. We compared *F. hamiltonii* to nine other clade C species, including *Farsetia occidentalis, Lobularia libyca, Notoceras bicorne, Parolinia ornata, Morettia canescens, Cochlearia borzaeana, Megacarpaea polyandra, Biscutella laevigata,* and *Iberis amara*. We conducted phylogenetic research on the 22 Brassicaceae species, which included members from 17 tribes and six clades.

**Results:**

The chloroplast genome sequence of *F.hamiltonii* of 154,802 bp sizes with 36.30% GC content and have a typical structure comprised of a Large Single Copy (LSC) of 83,906 bp, a Small Single Copy (SSC) of 17,988 bp, and two copies of Inverted Repeats (IRs) of 26,454 bp. The genomes of *F. hamiltonii* and *F. occidentalis* show shared amino acid frequencies and codon use, RNA editing sites, simple sequence repeats, and oligonucleotide repeats. The maximum likelihood tree revealed *Farsetia* as a monophyletic genus, closely linked to *Morettia*, with a bootstrap score of 100. The rate of transversion substitutions (Tv) was higher than the rate of transition substitutions (Ts), resulting in Ts/Tv less than one in all comparisons with *F. hamiltonii*, indicating that the species are closely related. The rate of synonymous substitutions (Ks) was greater than non-synonymous substitutions (Ka) in all comparisons with *F. hamiltonii*, with a Ka/Ks ratio smaller than one, indicating that genes underwent purifying selection. Low nucleotide diversity values range from 0.00085 to 0.08516, and IR regions comprise comparable genes on junctions with minimal change, supporting the conserved status of the selected chloroplast genomes of the clade C of the Brassicaceae family. We identified ten polymorphic regions, including *rps8-rpl14, rps15-ycf1, ndhG-ndhI, psbK-psbI, ccsA-ndhD, rpl36-rps8, petA-psbJ, ndhF-rpl32, psaJ-rpl3,* and *ycf1* that might be exploited to construct genuine and inexpensive to solve taxonomic discrepancy and understand phylogenetic relationship amongst Brassicaceae species.

**Conclusion:**

The entire chloroplast sequencing of *F. hamiltonii* sheds light on the divergence of genic chloroplast sequences among members of the clade C. When other *Farsetia* species are sequenced in the future, the full *F. hamiltonii* chloroplast will be used as a source for comprehensive taxonomical investigations of the genus. The comparison of *F. hamiltonii* and other clade C species adds new information to the phylogenetic data and evolutionary processes of the clade. The results of this study will also provide further molecular uses of clade C chloroplasts for possible plant genetic modifications and will help recognise more Brassicaceae family species.

**Supplementary Information:**

The online version contains supplementary material available at 10.1186/s12870-022-03750-2.

## Introduction

*Farsetia hamiltonii* Royle is a part of the Brassicaceae family, which is the enormous angiosperm family, with 52 tribes, 321 genera, about 4000 species [1–3], and is segregated into six clades (A, B, C, D, E, and F) [3–5]. The Brassicaceae family has a diverse range of medicinally useful plants [6]. *F. hamiltonii, also known as Freeden Booti, is a medicinal plant with over 20 species belongi*ng to the clade C and tribe Anastaticeae of the family Brassicaceae [6, 7].

*F. hamiltonii* is common in Pakistan and India's arid regions [8]. This plant is used medicinally to cure Arthritis, reduce soreness, burning sensations, and inflammation in joints, diabetes difficulties, gastrointestinal and infectious illness, and locals use its boiling extract to repair camel wounds [9, 10]. Hayat et al. [11] Experiments verified *F. hamiltonii's* anti-diabetes and anti-spasmodic effects. Other *Farsetia* species have also been claimed to have medicinal properties [11]. This plant was chosen for research because of the therapeutic value of the genus *Farsetia* [8].

As high-throughput sequencing technologies progress, chloroplast genomes holding a huge amount of genetic evidence have become increasingly accessible [12]. The chloroplast genome sequences constitute a key molecular source for phylogenetic studies [13–16]. In most angiosperms, chloroplast (cp) genomes are quadripartite, double-helical, and spherical [17]. The chloroplast genome has been considered an "ultra-barcode" for species recognition [18] and phylogenetic analysis [19]. The chloroplast genomes are appropriate for inferring phylogenic analysis due to their conserved genetic research, slowly changing traits, and uni-parental hereditary data [17, 20, 21].

As a result, multiple studies have used chloroplast genomes to explore phylogenetic relationships in the Brassicaceae family [12, 22, 23]. However, the systematic position of the *Farsetia* genus remains unknown because there is no published literature supporting their phylogenetic position based on the chloroplast genome. Despite the therapeutic relevance of the *Farsetia* genus, there is a scarcity of data on molecular evidence and chloroplast genomes. On NCBI, there is one whole chloroplast genome of *F. occidentalis* and one partial genome of *F. stylosa* [24]. This evidence is insufficient to support the *Farsetia* genus evolutionary dynamics. More chloroplast genomes must be sequenced and analyzed to understand the evolutionary features and species detection of the *Farsetia* genus and Clade C of the Brassicaceae. Here we reported the chloroplast genomic sequence of *F. hamiltonii* and made comparative investigations with *F. occidentalis* and eight other members of Clade C of Brassicaceae including *Lobularia libyca, Notoceras bicorne, Parolinia ornata, Morettia canescens*, *Cochlearia borzaeana, Megacarpaea polyandra, Biscutella laevigata,* and *Iberis amara*. We also performed phylogenetic analysis among 22 species of the Brassicaceae family, covering 17 different tribes and six clades. This study will help to add-up significant molecular and phylogenetic data to the account of *Farsetia* species as well as Clade C of family Brassicaceae for species detection and understanding phylogenetic details.

## Materials and methods

### Collection of plant material, DNA Extraction, and genome sequencing

*F. hamiltonii* plants were obtained from the Lesser Cholistan desert in Punjab, Pakistan. Healthy and fresh-cut leaves from the plants were chosen. The DNA extraction was done using the technique followed by Ahmed et al. [25] with a few adjustments: via 1 µL 2‐Mercaptoethanol and precipitating DNA with absolute ethanol after washing with 70% ethanol. The quantitative and qualitative estimation of extracted DNA was done with the help of Nanodrop (Thermo Scientific) and 1% agarose gel electrophoresis. A complete genome shotgun by the Paired-end library of 150 bp was created with the help of Illumina Hiseq2500 at Beijing Institute of Genomics (BIG), Beijing, China.

### de novo assembly and annotations

FastQC analysis [26] was used to estimate raw data qualitatively. Small-sized reads are assembled into elongated contigs with the help of Velvet 1.2.10 [27] by setting 71, 91, 101, & 111 kmer values. The contigs produced by Velvet 1.2.10 were de novo assembled with the help of Geneious Prime 2021.1.1 [28]. Sequence scaffolding was visually examined to define margins of large single copy (LSC), inverted repeats (IRs), and small single copy (SSC) regions. GeSeq [29] and CpGAVAS with default parameters [30] were employed to annotate the assembled cp genome sequence of *F. hamiltonii*. The confirmation of annotations was done by making pair-wise alignment of the *F. hamiltonii* genome with two closely similar genomes, *F. occidentalis* (MK637823) and *F. stylosa* (KY912025), aligning them with MAFFT (Multiple Alignment with Fast Fourier Transform) [31]. The tRNA genes were reconfirmed via tRNAscan-SE 1.23 [32]. Through matching sequencing short reads with their complete de novo assembled cp genome using BWA [33], the average sequencing coverage intensity for the assembled *F. hamiltonii* genome was determined and anticipated in Tablet [34]. OGDraw v1.2 [35] was employed to create a circular map of the cp genome. The finalized chloroplast genome of F. hamiltonii was submitted to GenBank and assigned the accession number MT884003. The unprocessed data produced in the present work was deposited to Sequence Read Archive (SRA) portal in the project number PRJNA660981.

### Amino acid frequency, RSCU, RNA editing site prediction

We obtained the chloroplast genome of *F. occidentalis* from NCBI to conduct a basic within genus comparison to confirm annotations and genome organization and to compare with *Farsetia hamiltonii*. Amino acid frequency was examined using Geneious Prime 2021.1.1. RSCU (Relative Synonymous Codon Usage) in protein-coding sequences of these two *Farsetia* species was examined using MEGA-X [36]. For the estimation of RNA editing sites in 26 genes, PREP-cp (Predictive RNA Editors for Plants Chloroplast) was utilized [37].

### Synthesis SSRs and oligonucleotide repeats

Simple Sequence Repeats (SSRs) were examined in chloroplast genomes of *F. hamiltonii* and *F. occidentalis* by a Perl script MISA (MIcroSAtellite Identification Tools) [38]. SSRs repeats were observed by limiting the number of repetitions to 10 for mononucleotide, five for di- and four for trinucleotide, and three for tetra Penta and hexanucleotide SSRs. REPuter program [39] was employed to discover complementary (C), palindromic (P), forward (F), and reverse (R), oligonucleotide repeats by a minimum repeat size of 10 bp, the edit distance of two, and maximum computed repeats as 100 to discover repeat pair within 90% correspondence.

### Phylogenetic analysis of family Brassicaceae

Phylogenetic relationships were inferred among 24 species, 22 of which belong to 17 distinct tribes and six clades (A, B, C, D, E & F) of the Brassicaceae family [5], and two outgroups of the *Calotropis* genus (*Calotropis procera, Calotropis gigantea*) of the Apocynaceae family. Six species, including *Farsetia hamiltonii*, are members of Clade C tribe Anastaticeae, whereas 16 representative species are members of 16 other tribes. To do this, the selected species were obtained from NCBI, protein-coding sequences from every species were extracted, and the sequences were concatenated in Geneious Prime 2021.1.1. These protein-coding sequences were aligned through MAFFT in Geneious Prime 2021.1.1. The best-fit model TVM + F + I + G4 was used to reconstruct the phylogenetic tree. The maximum likelihood tree was created online in Galaxy (https://usegalaxy.org) using IQ-TREE by selecting the maximum likelihood (ML) technique and 1000 bootstrap replications through Ultrafast bootstrap parameters [40]. We finalized the tree presentation with the help of iTOL (interactive tree of life) [41], which we used online.

### IR contraction and expansion, Comparative analysis of *F. hamiltonii* with Clade C members

Based on the phylogenetic analysis results, the cp genomes of nine species of Clade C five tribes, including tribes Anastaticeae (*Farsetia occidentalis*, *Lobularia libyca, Notoceras bicorne, Parolinia ornata,* and *Morettia canescens)*, Tribe Cochlearieae (*Cochlearia borzaeana*)*,* Tribe Megacarpaeeae *(Megacarpaea polyandra*)*,* Tribe Biscutelleae *(Biscutella laevigata*)*,* and Tribe Iberideae *(Iberis amara*)*,* were compared to that of *F. hamiltonii*. Using annotations of their available cp genomes in GenBank, IRscope [42] highlighted the LSC/IRB/SSC/IRA links between species. The basic comparison was based on a manual examination of the cp genomes using Geneious Prime 2021.1.1.

### Synonymous (Ks) and non-synonymous substitutions rates (Ka)

We observed Ks (synonymous), Ka (non-synonymous) substitution, and Ka/Ks ratio using pairwise alignment of protein-coding sequences of *F. hamiltonii* and other nine selected species (*F. occidentalis, L. libyca, N. bicorne, P. ornata, M. canescens, C. borzaeana, M. polyandra, B. laevigata,* and *I. amara*). We used *F. hamiltonii* as the reference member in each pair of alignments to make pairwise alignments with every gene of the selected species. We took out 77 common protein-coding sequences of selected chloroplast genomes and prepared 693 pair-wise alignments by MAFFT with the help of Geneious Prime 2021.1.1.DnaSP [43] was employed to examine Ka and Ks substitutions.

### Determination of SNPs and Indels mutations

For contrast comparison, the LSC, SSC, and IR regions of *F. occidentalis, L. libyca, N. bicorne, P. ornata, M. canescens, C. borzaeana, M. polyandra, B. laevigata,* and *I. amara* were pairwise aligned with relevant parts of the *F. hamiltonii* cp genome utilized as a reference genome using MAFFT alignment [31]. SNPs (Single Nucleotide Polymorphisms) were determined in Geneious Prime 2021.1.1 to compute the number, coordinate locations, and type of substitutions (transition and transversion). DnaSP [43] was utilized to identify InDels mutations in every segment of the pairwise aligned cp genomes. Alignment length, inDel average length, k(i) inDel diversity and Pi(i) inDel diversity also were computed for each site.

### Nucleotide diversity and highly polymorphic regions in *F. hamiltonii* and *F. occidentalis*

Nucleotide diversity (π) was examined in IGS (intergenic spacer regions), CDS (protein-coding sequences), and intronic regions of *F. hamiltonii, F. occidentalis, L. libyca, N. bicorne, P. ornata, M. canescens, C. borzaeana, M. polyandra, B. laevigata,* and *I. amara*. A total of 910 regions with more than 200 base pairs in length were extracted, including 58 protein-coding genes, 25 IGS, and eight intronic regions observed commonly in all species. Multiple alignments of 91 regions from ten species were created using MAFFT alignment in Geneious Prime 2021.1.1, and nucleotide diversity (π) was calculated by dividing the figure of mutations by the silent length of the alignment (alignment without gaps) [44]. It was further confirmed using DnaSP [43]. Ten regions with higher nucleotide diversity were chosen to show polymorphic regions between *F. hamiltonii* and the other nine species of Clade C of the Brassicaceae family.

## Results

### *F. hamiltonii* cp genome structure

**Fig. 1:**
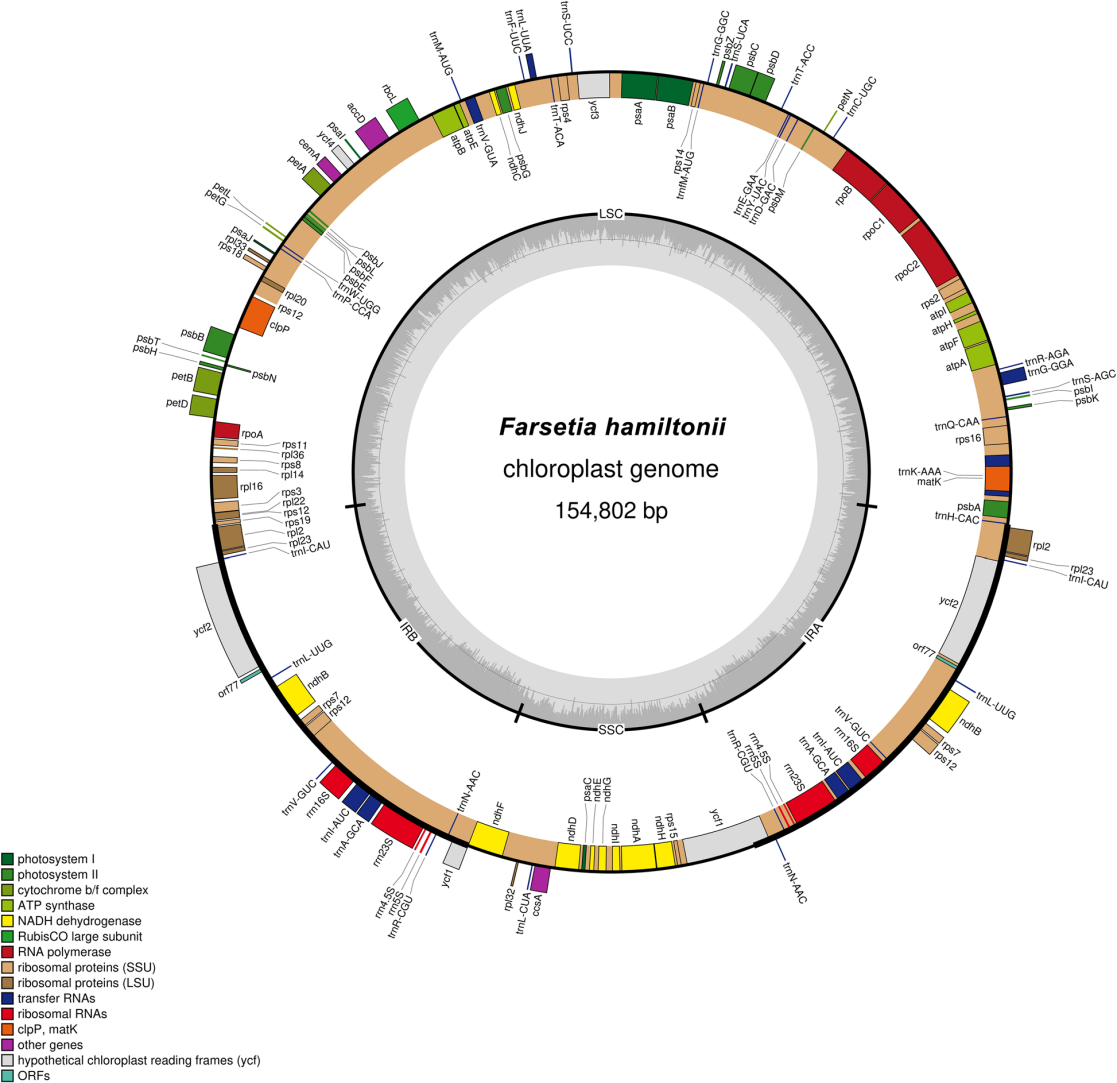
*F. hamiltonii* cp genome map. The genes on the circle's rim are transcribed clockwise, whereas those on the interior are transcribed anticlockwise. The hue of a gene identifies protein-coding genes on the base of their function. The inner-circle displays the genome's AT and GC makeup as light Grey and dark, respectively. Inverted Repeats are designated by the letters IRb and IRa, Small Single Copy regions by SSC, and Large Single Copy regions by LSC

By using Illumina HiSeq2500, the paired-end sequencing with 150 bp reads constructed 13.6 GB of raw data for *F. hamiltonii*. The de novo assembled cp genome of *F. hamiltonii* possessed an average coverage depth of 1438. The cp genome of *F. hamiltonii* is 154802 bp in size (Fig. 1) comprised of the SSC region of 17,988 bp, LSC region of 83,906 bp, and two inverted repeats of 26,454 bp length. It displays an overall 36.30% GC content while IRs exhibit more GC content 42.40% than the LSC 34% and SSC 29.1%. *F. hamiltonii* chloroplast genome has 132 genes, having 87 protein-coding genes, eight rRNA genes, and 37 tRNA genes. Twenty genes are replicated in the inverted repeat regions (Table 1). In *F.hamiltonii*, 21 genes containing introns (13 protein-coding genes and eight tRNA genes) were found, alongside 19 genes having one intron while *ycf3* and *clpP* possess two introns (Table S1). Trans-splicing was discovered in the *rps12* gene. The *ycf1* gene begins in IRs and terminates in an SSC region, having a reduced duplication in the IRb region.

**Table 1:** The full cp genome of Farsetia hamiltonii is described in detail

FEATURE	*Farsetia hamiltonii*
COMPLETE CP GENOME	154,802
Length of SSC (bp)	17,988
Length of LSC (bp)	83,906
Length of IR (bp)	26,454
CG content overall	36.30%
AT content overall	63.72%
LSC region GC %	34.00%
SSC region GC %	29.10%
IR region GC %	42.40%
Total Genes	132
Protien Coding Genes (CDS)	87
tRNA genes	37
rRNA genes	8
Duplicated Genes in IRS (Total)	20
no. of rRNA genes in IR	4
no. of tRNA genes in IR	7
no. of Protein-coding (CDS) genes in IR	9
Total Intron containg genes (IGSs)	21
ICGs Protien coding (CDS)	13
ICGs in tRNA	8
ICGs in rrna	0
1 Intron containing Genes	19
2 Intron containing Genes	2 (*ycf3, clpP*)
Trans-spliting Event	*rps12*
ACESSION NUMBER	MT884003

### RSCU and Amino Acid frequencies

**Fig. 2:**
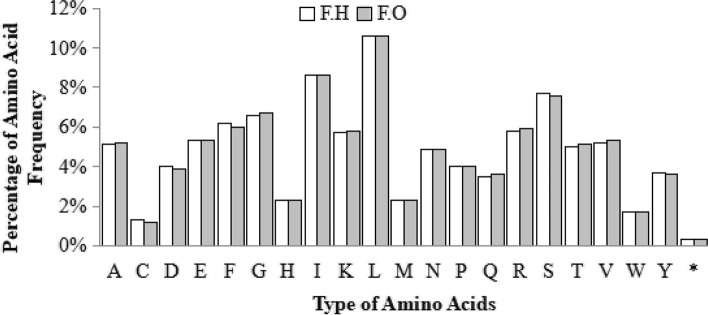
Comparative analysis of amino acids and their frequency (%) of *F. hamiltonii* and *F. occidentalis*

A basic comparative analysis of *Farsetia hamiltonii* was performed with *Farsetia occidentalis* to confirm annotations and genome organization. The similarities in RSCU levels and amino acid frequencies were investigated in both cp genomes. *F.hamiltonii* has 80,043 bp of coding sequences with 51,600 codons, while *F. occidentalis* have 79,134 bp of coding sequences with 51,597 codons. The most prevalent amino acid in both cp genomes was leucine, which was found 10.6% of the time, followed by isoleucine, which was found 8.6% of the time. Cysteine was identified as a rare amino acid in the cp genomes of *F. hamiltonii* and *F. occidentalis*, with 1.3 percent and 1.2 percent abundance, respectively (Fig. 2 and Table S2). We found RSCU values for 64 codons, out of which 34 mutant codons possessed RSCU values greater than one, indicating that they are largely employed in *F. hamiltonii* and *F. occidentalis* to code for specific amino acids. The AGA codon, which codes for Arginine, had the highest usage bias (2), whereas CGC, which also codes for Arginine, had the lowest (0.5) usage bias in both species. Three similar stop codons TAA, TAG, and TGA observed in *F. hamiltonii* and *F. occidentalis*. With RSCU equal to 1.00, codons and TGG (Tryptophan) in both cp genomes revealed no bias (Table S3).

### RNA editing sites

**Fig. 3:**
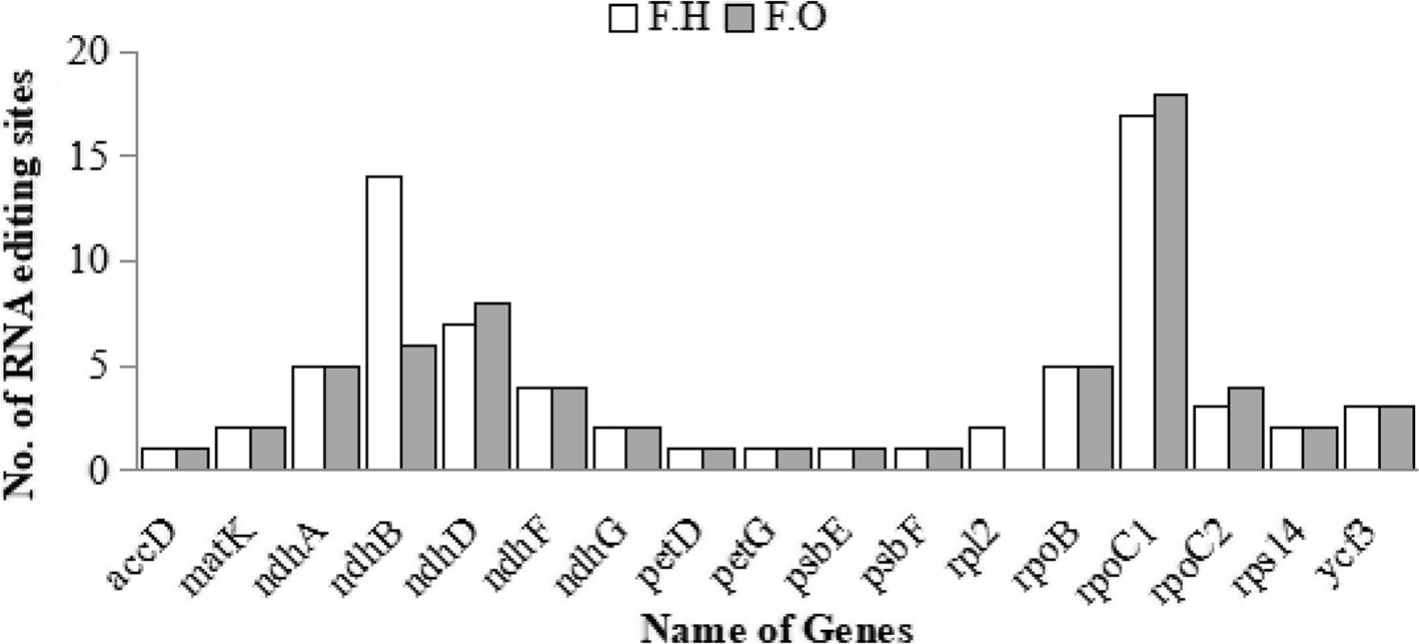
Comparative analysis of RNA editing sites of *Farsetia hamiltonii* and *F. occidentalis*. It shows 17 genes with the number of RNA editing sites found in both species

In *F. hamiltonii*, the PREP-cp perceived 71 RNA editing sites in 17 genes and 64 RNA editing sites in 16 genes in *F. occidentalis*. The higher figure of RNA editing sites was determined in *rpoC1* with 17 sites in *F. hamiltonii* while 18 sites in *F. occidentalis.* The *ndhB* gene, which has 14 sites in *F. hamiltonii*, and the *ndhD* gene, which has 8 sites in *F. occidentalis*, have the 2^nd^ largest number of sites (Fig. 3). Among the 71 RNA editing sites found in *F. hmailtonii*, altering a nucleotide at the 1^st^ position of a codon resulted in 27 (38%) editing sites, whereas shifting a nucleotide at the 2^nd^ position of a codon resulted in 44 (62%) editing sites. While in *F. occidentalis,* alteration of the first nucleotide was determined in 23 RNA editing sites (36%) out of 64 sites, and change in the second position was observed in 41 editing sites (64%). Most of the RNA editing sites were found in codons encoding Serine, with 21 sites (30%) in *F. hmailtonii* and 19 sites (30%) in *F. occidentalis*. In *F. hmailtonii*, 62 percent of Serine was converted to Leucine, and 38 percent of Serine was converted to Phenylalanine; while in *F. occidentalis*, 68 percent of Serine was converted to Leucine, and 32 percent of Serine was converted to Phenylalanine. In both genomes, the second-largest conversions were found in a codon coding for Proline and the third-highest in a codon that codes for Threonine. Except for Proline, Serine, and Threonine, which were found in both species, all amino acids exhibited just one form of nucleotide conversion, while Arginine in *F. hamiltonii* also showed a different kind of conversion. Out of total RNA editing sites in *F. hamiltonii* 34% and *F. occidentalis* 31% conversions consequence in hydrophobic amino acids (including Proline, Alanine, Leucine). Conversions of non-polar to polar (29, 21), non-polar to non-polar conversions (58, 59), 55 polar to non-polar conversions, and 17 polar to polar amino acid conversions have also been identified in *F. hamiltonii* and *F. occidentalis* respectively. Table S4 lists the RNA editing sites in detail.

### Analyzing SSRs

**Fig. 4:**
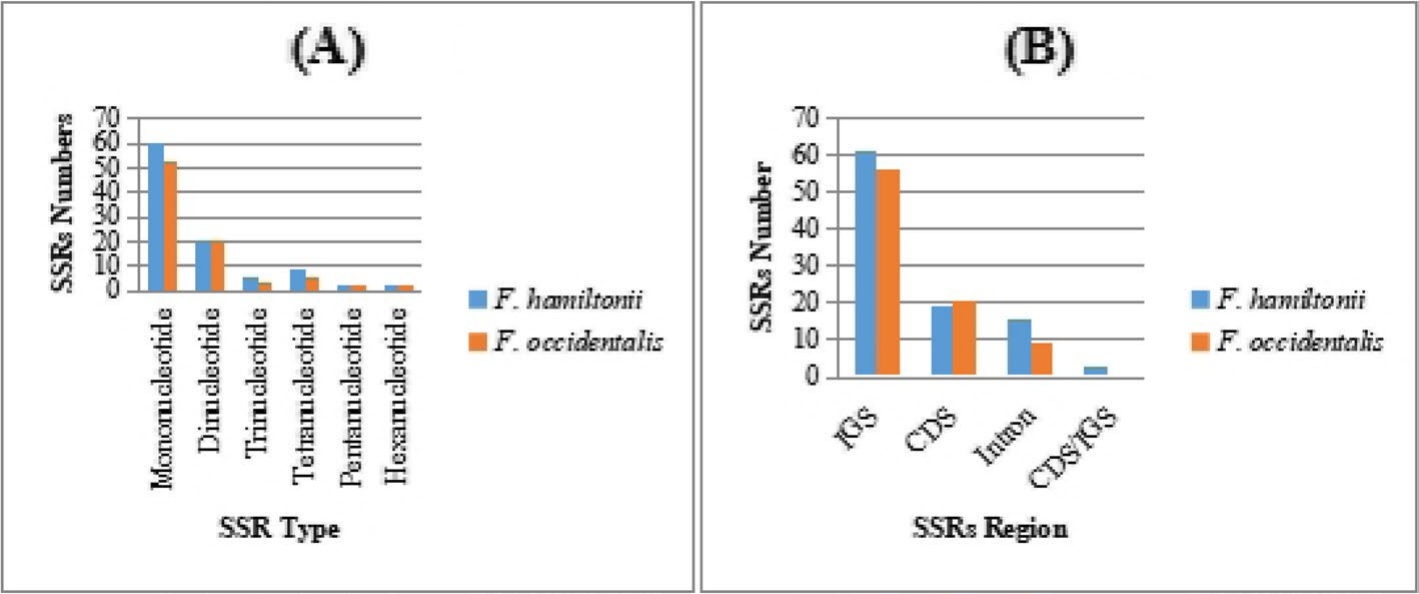
**A** Comparative analyses of the number of several types of SSRs of *Farsetia hamiltonii* and *F. occidentalis*. **B** Represent numerous SSRs found in functional cp genome areas such as IGS, CDS, intronic region, and shared regions

By using Perl script MISA we observed 99 SSRs in *F. hamiltonii* and 87 SSRs in *F. occidentalis* (Table S5). Most of SSRs (61%) were mononucleotides ranging from 10–18 repeat units in *F. hamiltonii,* while 10 to 20 repeat units in *F. occidentalis* (Table S6). 21 di-nucleotide in both 6 and 4 Tri-nucleotide, 9 and 6 tetra-nucleotide, 1 pentanucleotide, and 2 hexanucleotide repeats were observed in *F. hamiltonii* and *F. occidentalis* respectively (Fig. 4A). Almost mononucleotide SSRs including A/T motifs whereas *F. hamiltonii* showed 1 mononucleotide with a C/G motif. We observed 1 type (AT/AT) of di-nucleotide in *F. hamiltonii,* while two types AG/CT and AT/AT observed in *F. occidentalis*. Two types of tri-nucleotides (AAG/CTT and AAT/ATT), four types of tetra-nucleotides (AAAC/GTTT, AAAT/ATTT, AATT/AATT, AGAT/ATCT) and 1 type (AGCTCC/AGCTGG) of hexanucleotide motifs were observed in both cp genomes. The pentanucleotide was observed containing AAAAG/CTTTT motifs in *F. hamiltonii* and AATAC/ATTGT in *F. occidentalis* (Table S7). Most of the SSRs were situated in the LSC location (68 in *F. hamiltonii* and 53 in *F. occidentalis*) than in the SSC area (19 in *F. hamiltonii* and 20 in *F. occidentalis*) and after that in the IR region (12 in *F. hamiltonii* and 14 in *F. occidentalis*) (Table S5). The following sequence was observed in the ratio of SSRs in various parts: intergenic regions (62% in *F. hamiltonii* and 64% in *F. occidentalis*) > protein-coding regions (19% in *F. hamiltonii* and 24% in *F. occidentalis*) > intronic regions (16% in *F. hamiltonii* and 10% in *F. occidentalis*) (Fig. 4B). Also observed are the SSRs shared between CDS/IGS in both species.

### Analysis of oligonucleotide repeats

**Fig. 5:**
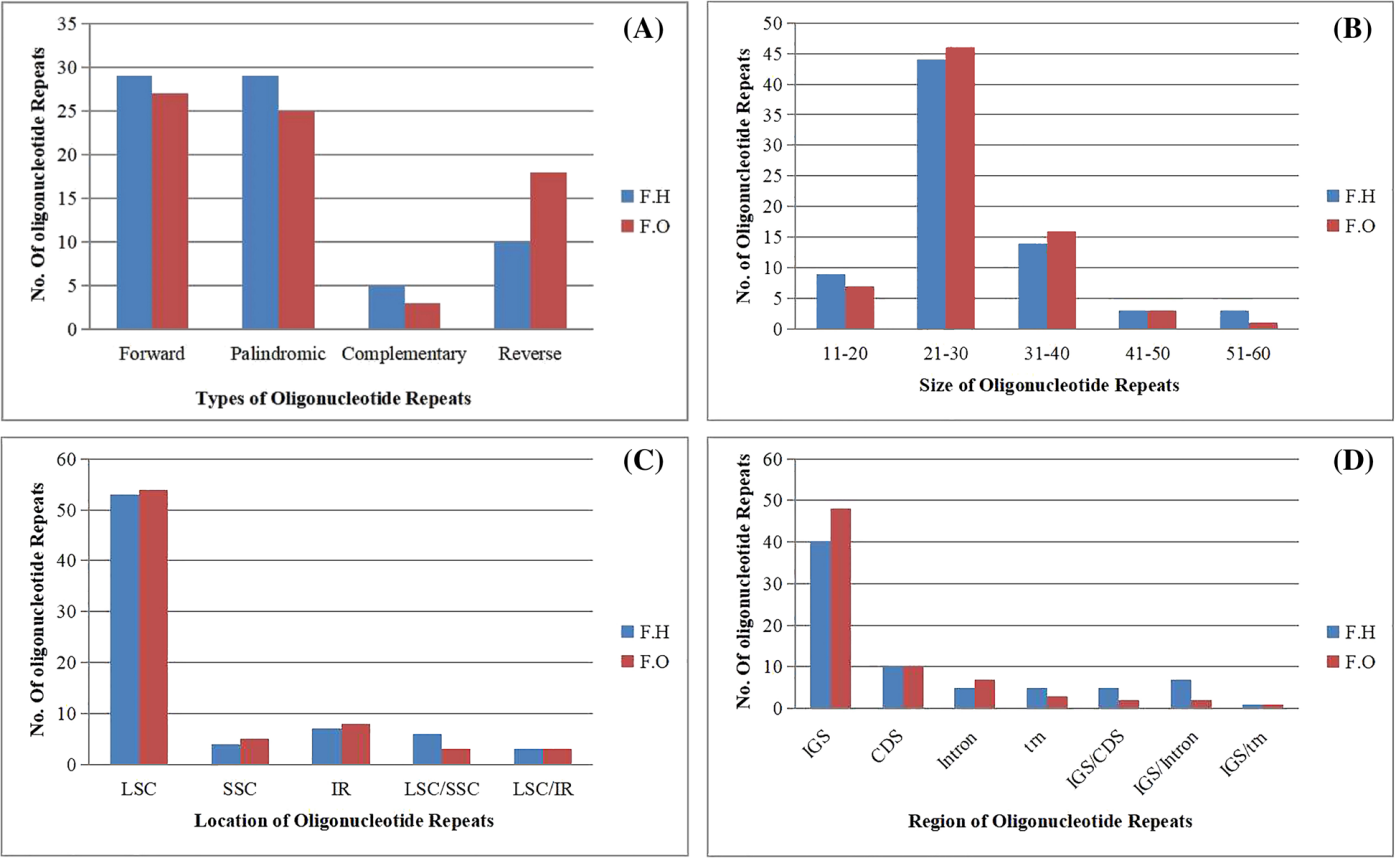
**A** The no. of four categories of oligonucleotide repeats revealedcountingreverse, complementary, palindromic, and forward repeats in F. hamiltonii and F.occidentalis. **B** Describe oligonucleotide repeats based on their size ranging from 20 to 56 bp in F. hamiltonii and F. occidentalis. **C** Characterize the no. of oligonucleotide repeats in the three regions and shared regions of the cp genome, LSC, SSC, IR, whereas LSC/IR and LSC/SSC depict mutual repeats in these zones, i.e. LSC/SSC denotes those repeats for which one repeat exists in LSC and other one exists in SSC. **D** Exhibit oligonucleotide repeats in functional units of *F. hamiltonii* and *F. occidentalis* chloroplast genomes. IGS, CDS, intron, and trn have been used to symbolize every functional region. Shared repetitions between two regions, such as IGS/CDS, IGS/Intron, and IGS/trn, have been detected

Using the REPuter software, we discovered oligonucleotide repeat sequences in the cp genomes of *F. hamiltonii* and *F. occidentalis* [39]. We found 73 oligonucleotide repeats in both species, (F = 29, *P* = 29, C = 5, and R = 10 in *F. hamiltonii* and, F = 27, *P* = 25, C = 3, and R = 18 in *F. occidentalis*) shown in Fig. 5 (A). The size of these repeats ranges from 20 to 56 bp (Fig. 5 B). LSC (53, 54), IR (7, 8), and SSC (4, 5) were discovered to have the most repeats in *F. hamiltonii* and *F. occidentalis*, respectively. We also discovered that LSC and IR have similar repeat patterns (3 in *F. hamiltonii* and 3 in *F. occidentalis*); and LSC/SSC (6 in *F. hamiltonii* and 3 in *F. occidentalis*) (Fig. 5 C). In intergenic spacer areas, the number of oligonucleotide repeats was greater (40, 48), followed by CDS (10, 10), intronic region (5, 7), then trn (5, 3); also observed mutual repeats in the regions IGS/CDS (5, 2), IGS/Intron (7, 2) and IGS/trn (1, 1), respectively in *F. hamiltonii* and *F. occidentalis* (Fig. 5 D). The location, position, and areas of repeats are all listed in Table S8.

### Phylogenetic analysis of family Brassicaceae

**Fig. 6:**
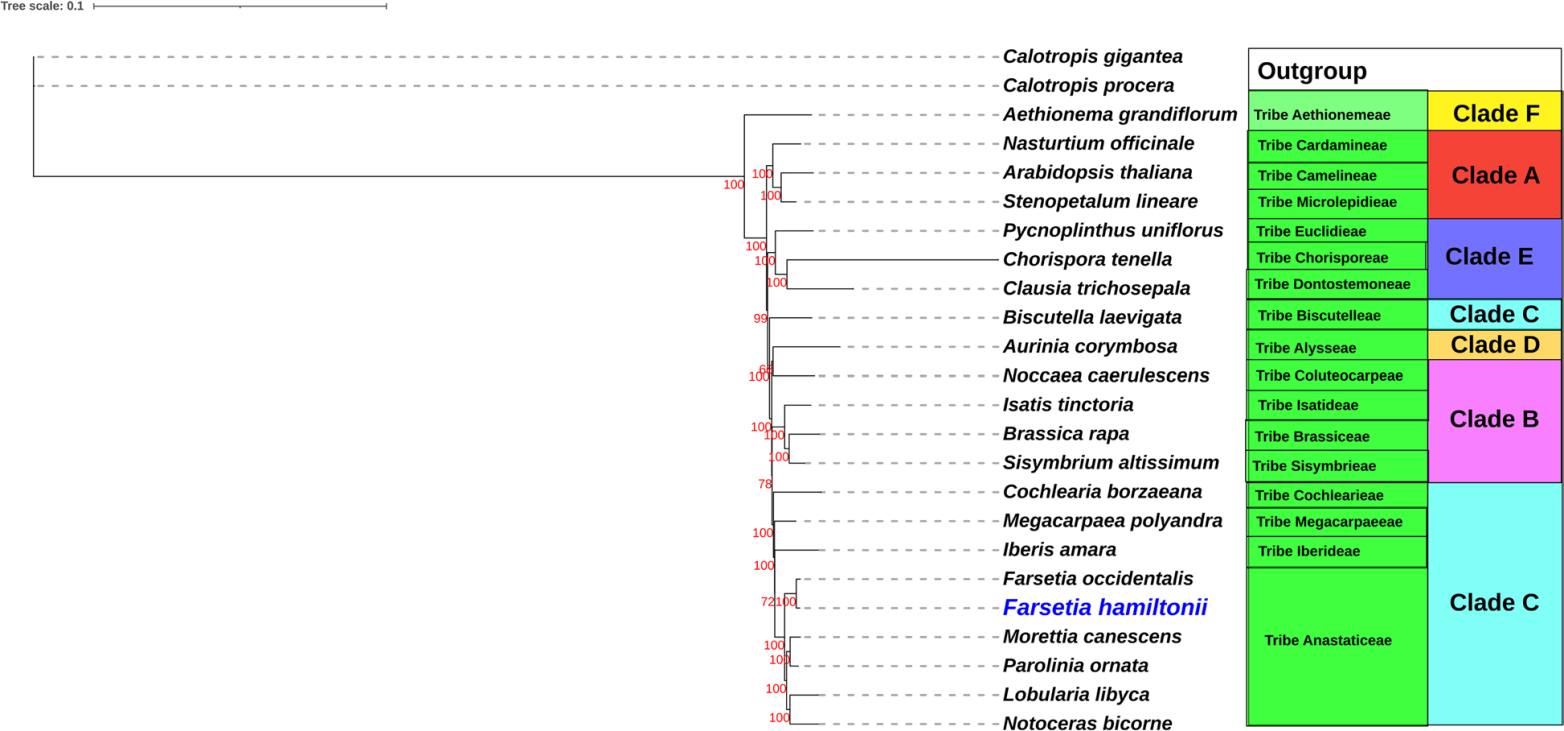
The maximum likelihood (ML) tree of Family Brassicaceae is based upon protein-coding genes. Calotropis procera and Calotropis gigantea represent the out-group (Family Apocynaceae). The genus Farsetia is closely related to the genus Morettia

For 22 taxa belonging to 17 different tribes and six clades of the family Brassicaceae [5], a maximum likelihood (ML) tree was rebuilt based upon protein-coding genes. *Calotropis procera* and *Calotropis gigantea* was chosen as an out-group from the Brassicaceae family. We used the NCBI to obtain 23 species with particular accession numbers for phylogeny inference (Table S9). The alignment of 24 species (23 from NCBI + *F. hamiltonii*) included 94,510 bp consensus sequence nucleotide positions with 89.9 percent pairwise identity. The phylogenetic tree produced 21 branches with bootstrap node values greater than 65 (Fig. 4). The bootstrap node values for 17 of these branches were 100. The phylogenetic tree demonstrated that the genus *Farsetia* is monophyletic, with four other genera of the tribe Anastaticeae*, Parolinia, Morettia, Lobularia,* and *Notoceras*, all displaying a strong bootstrap support value of 100. The tree also showed that the *Farsetia* genus is close to the *Morettia* genus. All of the Clade C selected genera bear a striking similarity to the *Farsetia* (Fig. 6).

### IRs contraction expansion and comparative analysis of *F. hamiltonii* with species of Clade C

**Fig. 7:**
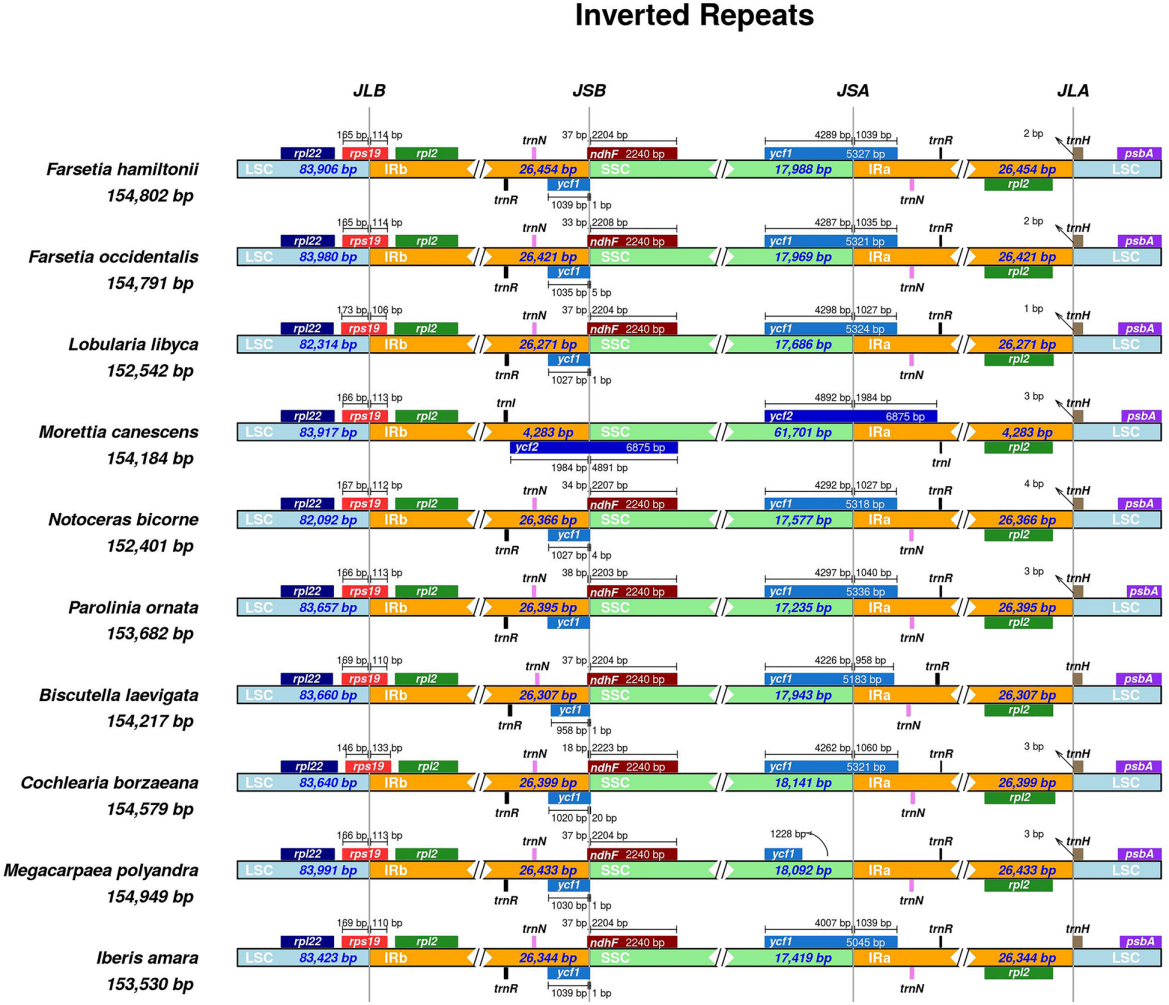
A look at the joints in the cp genomes of F. hamiltonii and nine additional Brassicaceae Clade C species. Genes in each species are transcribed straight right to left in the positive strand, while genes in the negative strand are transcribed from left to right. The coordinate position of each gene that begins or ends at the next junction is shown by an arrow. The T bar above or below denotes all of the genes that integrate from one part of the cp genome to the other. The length of the base pair integrating genes is represented by the T bars. The shown genes and distances at the junction points exhibit the genome-scale projection. IRb/LSC (JLB), IRa/LSC (JLA), IRb/SSC (JSA), and SSC/IRa (JSA) are the link parts connecting homologous sections of the genome (JSB)

Following phylogenetic results, we chose nine clades C species to compare with *F. hamiltonii*, including *F. occidentalis, L. libyca, N. bicorne, P. ornata, M. canescens, C. borzaeana, M. polyandra, B. laevigata,* and *I. amara*.To find divergence among the *F. hamiltonii* cp genome and nine linked Brassicaceae clade C species, these cp genomes were well conserved, as seen in Table 2. In summary, the entire chloroplast sequence length varies from 152,401 bp (*N. bicorne*) to 154,949 bp (M. polyandra), and every part of the quadripartite cycle was analogous throughout the chosen cp genomes. The total GC content of these cp genomes was likewise relatively close (36.1–36.6 percent). The gene composition in these cp genomes was identical, except for two genes absent from the C. borzaeana cp genome, rps12 and orf77. Fundamental changes in the length of IR regions impact the size of chloroplast genomes throughout time. The shrinkage and extension of IRs areas at the intersection of IRa/LSC, SSC/IRa, IRb/SSC, and LSC/IRb in *F.hamiltonii* and other nine species were compared (Fig. 5). Except for *M. canescens*, which has *ycf2* at both ends, all species have a working copy of the *ycf1* gene at the SSC/IRa border, as well as a pseudo copy (*ycf1*Ψ) at the IRb/SSC junction. The length of the *ycf1*Ψ gene ranges from 958 to 1039 bp. The *ndhF* gene is found at the IRb/SSC junction in all species except *M. canescens*, and it has the same length of 2240 bp. The *rps19* gene is found embedded in IRb sections at the LSC/IRb junction, whereas the *rpl2* gene is entirely in the IR region and the *rpl22* gene is found exclusively in the LSC in all species. The *trnH* gene is entirely visible in the LSC region at the IRa/LSC joint, whereas *psbA* is shown in the LSC region. The existence of identical genes at each junction of the chloroplast genomes also suggested similarities in gene content. Figure 7 shows a comprehensive comparison of IR contraction and expansion.

**Table 2:** Shows the results of cp genome comparison across 10 Brassicaceae clade C species

Genome Features	*F. hamiltonii*	*F. occidentalis*	*L. libyca*	*N. bicorne*	*P. ornata*	*M. canescens*	*C. borzaeana*	*M. polyandra*	*B. laevigata*	*I. amara*
Genome Size (bp)	154,802	154,791	152,542	152,401	153,682	154,184	154,599	154,949	154,217	153,530
Length of LSC (bp)	83,906	83,980	82,306	82,092	83,657	83,917	83,660	83,991	83,660	83,423
Length of SSC (bp)	17,988	17,969	17,686	17,577	17,235	17,794	18,141	18,092	17,943	17,419
Length of IR (bp)	26,454	26,421	26,275	26,366	26,395	26,227	26,399	26,433	26,307	26,344
GC content %	36.3	36.2	36.5	36.6	36.4	36.4	36.1	36.1	36.4	36.6
Total No. of genes	132	132	131	132	132	131	127	132	132	132
Protein Coding Genes	87	87	86	87	87	86	83	87	87	87
No. of tRNA genes	37	37	37	37	37	37	36	37	37	37
No. of rRNA genes	8	8	8	8	8	8	8	8	8	8
Accession Nubmer	MT884003	MK637823	KY912029	MK637762	MK637776	KY912031	LN866844	MK637758	MK637669	MK637733

### Ka and Ks rate, Number of substitutions

Gene nucleotide substitution patterns, both non-synonymous (Ka) and synonymous (Ks), are key indications of gene evolution [23]. The Ka/Ks value is utilized to decide the charge of selection pressure upon protein-coding genes and measure the pace of gene divergence. Ka/Ks values greater than one, close to one, or less than one suggest that the gene is subjected to positive, unbiased, or pure selection, respectively [45].

**Fig. 8:**
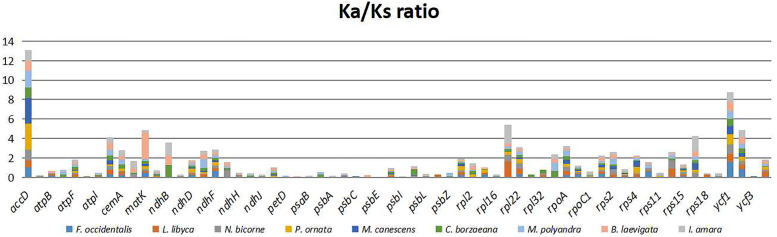
Displayed Ka/Ks Value for 32 genes, genes with zero Ka/Ks values are not shown here

The Ka/Ks value of the *F. hamiltonii* cp genome was determined in this work and compared to nine closely related species of Clade C of the Brassicaceae family, including *F. occidentalis, L. libyca, N. bicorne, P. ornata, M. canescens, C. borzaeana, M. polyandra, B. laevigata*, and *I. amara* (Fig. 8). To determine the Ka/Ks among the chosen cp genomes, 693 pair-wise alignments were done using MAFFT in Geneious Prime 2021.1.1 on 77 homologous protein-coding genes. Genes with not applicable (N/A) Ka/Ks ratios were set to zero. (Table S10). After deleting the genes with a Ka or Ks value of 0, the average Ka/Ks ratio was 0.31, showing that the genes in the *F. hamiltonii* cp genome were exposed to significant purifying selection pressures. In all comparisons, the bulk of genes had a Ka/Ks value of less than one, and their ratios were comparable, except the *matK, ycf1, ndhB, rpl20, accD,* and *rps16* genes, whose Ka/Ks value was elusive, as shown in Table 3. For example, the Ka/Ks value of rps16 was 1.60 in the comparison of *I. amara* vs. *F. hamiltonii*, while it was less than 1 in the other eight comparisons. The Ka/Ks value of *accD* was 0.71 less than one when compared to *L. libyca*, but it was more than one in the other eight comparisons, indicating it underwent strong positive selection in *F.hamiltonii*.

**Table 3:** List of 6 exceptional genes showing more than 1 Ka/Ks ratio for different species comparisons with *F. hamiltonii*

GENES	Species that Pairwise aligned with *F. hamiltonii*	Ks	Ka	Ka/Ks
***matK***	*Biscutella laevigata*	0.0187	0.0506	2.7059
***rps16***	*Iberis amara*	0.0366	0.0584	1.60
***rpl20***	*Iberis amara*	0.0117	0.023	1.965811966
	*Lobularia libyca*	0.0116	0.0192	1.655172414
***ycf1***	*Farsetia occidentalis*	0.0065	0.0102	1.57
	*Parolinia ornata*	0.0322	0.033	1.02
	*Iberis amara*	0.0747	0.0755	1.01
***ndhB***	*Iberis amara*	0.0034	0.0046	1.352941176
	*Cochlearia borzaeana*	0.0027	0.0034	1.259259259
	*Biscutella laevigata*	0.0034	0.0034	1
***accD***	*Parolinia ornata*	0.0134	0.0358	2.671641791
	*Morettia canescens*	0.0153	0.0394	2.575163399
	*Notoceras bicorne*	0.0269	0.0301	1.118959108
	*Cochlearia borzaeana*	0.0373	0.041	1.09919571
	*Iberis amara*	0.0349	0.038	1.088825215
	*Farsetia occidentalis*	0.0033	0.0035	1.060606061
	*Biscutella laevigata*	0.0392	0.0406	1.035714286
	*Megacarpaea polyandra*	0.0168	0.0283	1.68452381

The highest Ka/Ks ratio was reported in *matK* (2.71) when comparing *F. hamiltonii* to *B. laevigata*, and the second-highest in *accD* (2.67) when compared to *P. ornata* (Table S10).

### Investigating SNPs and InDel mutations analysis in *F. hamiltonii*

We found SNPs (single nucleotide polymorphisms) and InDels mutations in the LSC, IR, and SSC regions of *F. hamiltonii* by comparing them to nine other species of clade C using pairwise alignments of the respective regions. When comparing *F. hamiltonii* to *Cochlearia borzaeana*, the highest number of SNPs (4,324) was found, and the lowest when compared to *F. occidentalis* (500 SNPs). The transition rate was less than the rate of a transversion, resulting in the transition to transversion ratios of less than one in all of the species, except for the LSC region comparison of *M. polyandra* vs *F. hamiltonii* (Table 4). The LSC region had the highest rate of substitutions, followed by SSC then the IR sections (Table S12).

**Table 4:** Transition and Transversion substitutions, their ratio in LSC, IRs, and SSC

Region	*Pairwise alignment with Farsetia hamiltonii*	Transition substituations	Transversion substituaions	Ts/Tv
**Large Single Copy**	*Farsetia occidentalis*	142	213	0.6667
	*Lobularia libyca*	850	1016	0.8366
	*Notoceras bicorne*	916	1064	0.8609
	*Parolinia ornata*	580	641	0.9048
	*Morettia canescens*	702	762	0.9213
	*Cochlearia borzaeana*	1500	1697	0.8839
	*Megacarpaea polyandra*	996	954	1.0440
	*Biscutella laevigata*	1306	1340	0.9746
	*Iberis amara*	1276	1662	0.7677
**Inverted Repeat**	*Farsetia occidentalis*	6	15	0.4000
	*Lobularia libyca*	61	70	0.8714
	*Notoceras bicorne*	74	76	0.9737
	*Parolinia ornata*	44	56	0.7857
	*Morettia canescens*	45	65	0.6923
	*Cochlearia borzaeana*	96	121	0.7934
	*Megacarpaea polyandra*	66	76	0.8684
	*Biscutella laevigata*	77	90	0.8556
	*Iberis amara*	75	93	0.8065
**Small Single Copy**	*Farsetia occidentalis*	45	79	0.5696
	*Lobularia libyca*	273	375	0.7280
	*Notoceras bicorne*	260	359	0.7242
	*Parolinia ornata*	163	197	0.8274
	*Morettia canescens*	217	271	0.8007
	*Cochlearia borzaeana*	411	545	0.7541
	*Megacarpaea polyandra*	291	293	0.9932
	*Biscutella laevigata*	418	492	0.8496
	*Iberis amara*	384	480	0.8000

The LSC area included the highest no. of inDels, then the SSC region, while the inverted repeat regions had the fewest. The maximum number of inDels was found in the pairwise alignment of *F. hamiltonii* and *B. laevigata* (4,892). *N. bicorne* has the second-highest inDels (4,803), whereas *F. occidentalis* has the fewest (994). When compared to the results of other species, the alignment of *F. hamiltonii* with *B. laevigata, P. ornata,* and *M. canescens* had a considerable amount of inDels in the LSC, SSC, and IR regions, respectively. The findings of this comprehensive investigation of InDels, Average InDel Length, InDel Diversity per site Pi(i), alignment length, and InDel Diversity K(i) are shown in Table S11.

### Nucleotide diversity and highly polymorphic regions in *Farsetia* species

**Fig. 9:**
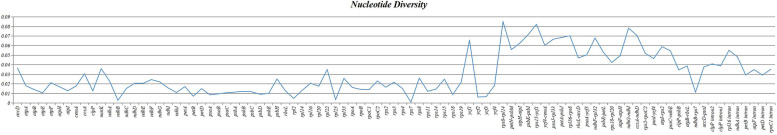
Nucleotide diversity (π) in different sections of the chloroplast genome in Clade C species. The X-axis denotes cp genome regions, whereas the Y-axis shows nucleotide diversity in each area

We analyzed IGS, CDS, and intronic regions in *F. hamiltonii* and nine other selected species of Clade C to assess nucleotide diversity and highly polymorphic regions. For this purpose, 25 IGS, 58 CDS, and 8 intronic regions with lengths greater than 200 base pairs were selected from each genome and made 91 multiple alignments. Intergenic spacers (IGS) areas had the greatest average nucleotide diversity (0.057), following the intronic regions (0.039) and coding regions (0.017). Nucleotide diversity rate range starting 0.00085 to 0.08516 (Table S13), The *rps8-rpl14* region, which comprises 19 mutations, has the highest nucleotide diversity (π = 0.08516). We selected ten highly polymorphic regions (Table 5), Out of ten; IGS regions have nine polymorphic sites, while the protein-coding region has one polymorphic site. Variations among the nucleotide diversity of the selected chloroplast genome regions are given in Fig. 9.

**Table 5:** Ten Highly Polymorphic regions among Clade C species

*Region*	Location	Nucleotide Diversity	Avg No. of Mutations	Region Length	Alignment Length
*rps8-rpl14*	IGS	0.08516	19.0	213	297
*rps15-ycf1*	IGS	0.08223	26.0	294	645
*ndhG-ndhI*	IGS	0.0783	24.9	281	458
*psbK-psbI*	IGS	0.07146	26.4	231	441
*ccsA-ndhD*	IGS	0.07077	15.2	194	248
*rpl36-rps8*	IGS	0.07028	32.8	441	554
*petA-psbJ*	IGS	0.06852	63.2	834	1177
*ndhF-rpl32*	IGS	0.06798	45.9	543	1269
*psaJ-rpl33*	IGS	0.06666	27.8	382	501
*ycf1*	CDS	0.06567	173.4	2641	5585

## Discussion

In this paper, we presented the complete cp genome of *F. hamiltonii* and compared it to *F. occidentalis* and eight other species of the Clade C of Brassicaceae including *L. libyca, N. bicorne, P. ornata, M. canescens, C. borzaeana, M. polyandra, B. laevigata,* and *I. amara*. The cp genome of *F. hamiltonii* was discovered to contain a distinctive quadripartite organization, having two inverted repeats, a large single copy, and a small single copy with incredibly similar structure and genomic contents to *F. occidentalis* and other Brassicaceae cp genomes [46, 47].To validate and demonstrate the close link within the *Farsetia* genus, we performed a basic comparison with the sole genome accessible, *F. occidentalis*. Codon usage bias is an attribute shared by every genome, and it has been hypothesized that it governs translation dynamics like reliability, accuracy, and protein folding [48]. Recent research found that codon use significantly influences the evolution of the cp genome [49, 50]. In this investigation, the more frequent amino acid in the cp genomes of *F. hamiltonii* and *F. occidentalis* was leucine, followed by isoleucine, and cysteine was identified as a rare amino acid. The findings were comparable to those observed in other cp genome studies [12, 22, 51–53]. We found RSCU values for 64 mutant codons, 34 of which had RSCU values larger than one, including three stop codons. In both species, the arginine with codon AGA had the highest usage bias, while the arginine with codon CGC had the lowest usage bias. Codons ATG and TGG indicated no bias when RSCU was equal to 1.00. These findings are correspondent to prior cp genomic studies [53–56], indicating that usage bias of particular codons was induced by adaptation evolution of the chloroplast genomes or by the compositional bias of the large concentration of A/T.

RNA editing is a type of post-transcriptional alteration that could have a major impact on the sequencing and performance of associated proteins and genetic material [51]. In *F. hamiltonii*, we found 71 RNA editing sites in 17 genes whereas 64 in 16 genes in *F. occidentalis*. The *rpoC1* gene, which encodes for DNA-dependent RNA polymerase, had the highest quantity of RNA editing sites. Among the RNA editing sites discovered in *F. hmailtonii* and *F. occidentalis*, changing a nucleotide at the second position was more common than other position shifts. The bulk of the RNA editing sites was discovered in Serine codons, with the largest conversion into Leucine. It corresponded to the general characteristics of chloroplast gene RNA editing in higher plants [51, 57–59]. This demonstrated that, through the functionality of RNA editing, a sole gene might translate a diversity of protein products, thus enhancing the genetic data of the genome [58] and evaluating the trends and transmission of RNA editing in substantial plants provides information on the evolutionary configuration of RNA editing while also assisting us in clearer grasp its biological functions [59]. Furthermore, RNA editing can modify the encoding amino acid and the primary, secondary, and tertiary organizations of proteins, which may be needed for their role [51, 59].

In addition, we looked at SSRs and oligonucleotide repeats found in the cp genomes of *F. hamiltonii* and *F. occidentalis*. The concentration of repeats in the cp genomes may also be utilized to identify highly polymorphic regions of the genome that could be employed to generate molecular markers for phylogenetic inference [60–63]. SSRs make up a large fraction of the genomes of eukaryotic organisms and can be utilized as extremely insightful genetic markers in polymorphic studies, comparative population genomics, genetic map development, and crop improvement [64, 65]. Within the cp genome, the SSRs also had a role in numerous forms of alternations, reductions, insertion, and huge variations [66].

The longest SSR type was hexanucleotides (AGCTCC/AGCTGG); the most abundant SSR type (61%) were mono-nucleotides with two repeat forms, A and T, in both cp genomes, while *F. hamiltonii* also possesses one C type mononucleotide; then dinucleotide categories composed of AT/TA. Similar results were reported in other species of Brassicaceae; *Sinapis alba* [22], *Brassica napus* [67], *Raphanus sativus* [23], and *Nasturtium officinale* [53], demonstrating that small polythymine (polyT) or polyadenine (polyA) repeats are widespread characteristics of the chloroplast genomes [68]. Two hexanucleotide simple sequence repeats were observed in the chloroplast genomes of *Farsetia* genus and *Sinapis alba* [22], while not in cp genomes of *B. napus*, *R. sativus*, and *N. officinale*. Such findings imply that SSRs change the chloroplast genomes and play a constructive role in detecting genomic variability between species [22].

In IGS, we found a lot of oligonucleotide repeats, then protein-coding regions, and finally intronic regions. In comparison to other repeats, palindromic repeats were abundant. In other angiosperms, a similar trend of oligonucleotide repeat transmission was discovered [69–72]. However, rather than intergenic spacer areas, some findings show that oligonucleotide repeats were abundant in protein-coding regions [73]. In comparison to SSC and inverted repeat areas, an increasing quantity of oligonucleotide repeats in LSC was determined. The SSRs and oligonucleotide repeats observed can play a valuable part in population genetics and phylogenetic investigations [72].

With the advancement of scale-up sequencing methods, cp genomes holding a huge amount of genetic evidence have become increasingly accessible [12]. The cp genome sequences are an important molecular source for phylogenetic studies [13–16]. Brassicaceae is a plant family with 52 tribes, 321 genera, and around 4000 species [1–3]. It is separated into six clades A, B, C, D, E, and F [4, 5]. As a result, multiple reports have used cp genomes to identify phylogenetic connections in the Brassicaceae family [12, 22, 23]; however the systematic position of the *Farsetia* genus remains unknown because there is no published literature confirming their phylogenetic position based on Chloroplast genome. *Farsetia*, an essential medicinal plant of the Brassicaceae family clade C [74], provides significant genetic assets for creating and identifying other valuable species. Understanding evolutionary links between *F. hamiltonii* and the other 21 species belonging to 17 other Brassicaceae tribes may guide the genetic variation of beneficial genes into comparable species. Our findings revealed a close relationship between the *Farsetia* and *Morretia* genera. All of the other Brassicaceae family genera are considered to bear a striking similarity to the *Farsetia* genus. These studies also demonstrated that the *Farsetia* genus and four other chosen members (*Parolinia, Morettia, Lobularia*, and *Notoceras*) of the tribe Anastaticeae are part of the same subgroup, which supports the *Farsetia* genus place within this tribe and Clade C [4, 5, 75].

Even though the cp genome is stable throughout the plant lineage, the size of the cp genome and its regions can alter because of IR contraction and expansion [73, 76, 77]. According to previous studies in various angiosperm plants, changes in the size and location of genes at the junction of inverted repeats result in variances in total genome boundaries [78]. Here, we compared the inverted repeat regions of *F. hamiltonii* and the selected nine species of the Clade C of Brassicaceae, including *F. occidentalis*, *L. libyca, N. bicorne, P. ornata, M. canescens, C. borzaeana, M. polyandra, B. laevigata,* and *I. amara.* With slight changes, the results showed similarities at the crossroads of four sections IRB/SSC/IRA/LSC, and surrounding genes of all species. Our findings support this hypothesis, demonstrating that the IR area is more conserved and that most substitutions occur in the SSC and LSC regions. Similar findings have been made in other plastid genomes [79–82]. In terms of gene content and gene organization, the chloroplast genomes are either conserved [72, 76, 83] or extensively polymorphic [84–87]. The comparative analysis of the basic genomes of *F. hamiltonii* and nine Clade C species revealed an extremely preserved organization of gene data, intron information, and gene structure. Conversely, the dimensions of cp genomes differed because of the changing length of IGS sites and IRs, as previously reported [55, 78, 88].

The pattern of Ks and Ka nucleotide substitutions is a known phenomenon for quantifying genomic evolution, and the Ka/Ks value shows selection pressures on genes [5, 45]. Purifying, impartial, and positive selections are experienced by genes with Ka/Ks less than one, Ka/Ks equal to one, and Ka/Ks more than one, respectively [45]. The Ka/Ks value of the *F. hamiltonii* cp genome was determined in this work when compared to nine closely related species of Clade C of the Brassicaceae family, including *F. occidentalis, L. libyca, N. bicorne, P. ornata, M. canescens, C. borzaeana, M. polyandra, B. laevigata*, and *I. amara*. The average Ka/Ks ratio was less than one, representing that the genes in the *F. hamiltonii* cp genome were subjected to clear purifying selection. Most of the genes had Ka/Ks values less than one in all comparisons and were consistent throughout, except for the *accD, ndhB, matK, ycf1, rpl20*, and *rps16* genes, whose Ka/Ks values were different. The Ka/Ks value smaller than one implies that there are more synonymous substitutions (Ks) than non-synonymous substitutions (Ka) in all comparisons with the *F. hamiltonii*, which is corroborated by similar investigations in several Brassicaceae members [4, 12, 22, 75]. The *accD* gene Ka/Ks ratio was more than one, indicating that it has undergone significant positive selection in *F.hamiltonii*. Similar findings have been obtained in prior research [12, 45].

In all species comparisons with *F. hamiltonii*, the no. of substitutions and inDels was highest in the LSC, then the SSC, and lowest in inverted repeats. These results supported prior observations showing, in contrast to inverted repeats, which are more conserved, substitutions and indels are more prevalent in SSC and LSC zones [62, 89]. We reported a Ts/Tv ratio smaller than one because the number of transversion substitutions (Tv) was larger than the rate of transition substitutions (Ts). Other investigations on the cp genomes of angiosperms and gymnosperms have shown similar findings [90–92]. As proxies, these InDels and substitutions can be used to infer mutational hotspot sites [62, 76, 78].

To estimate nucleotide diversity and highly polymorphic areas, we examined IGS, CDS, and intronic regions in *F. hamiltonii* and nine additional Clade C species. Intergenic spacer (IGS) regions exhibited the highest average nucleotide diversity, followed by intronic and coding regions. The finding that protein-coding genes have little nucleotide diversity supports the assumption that they are more conserved in Brassicaceae species [3, 12, 22, 93, 94]. Nucleotide diversity varies from 0.00085 to 0.08516; such low nucleotide diversity values indicate plastome structure conservation in clade C members, and a lower rate of nucleotide diversity has been seen in numerous other plants [95–98].

In this work, we reported ten highly polymorphic areas as potential molecular markers, including the *ycf1* gene, which was also reported in the previous studies as a potential marker for identification among the cp genomes [79, 99, 100]. These regions could be used to generate reliable and credible markers among the Brassicaceae family. More research into the Farsetia genus is required to prove the legitimacy of these markers.

## Conclusion

The complete *F. hamiltonii* cp genome was de Novo assembled employing the Illumina Hiseq platform in this study. The *F. hamiltonii* cp genome is a quadripartite cycle of 154,802 bp containing 79 protein-coding genes, four rRNA genes, and 30 tRNA genes. 99 SSR loci and 73 Oligonucleotide repeats were found in *F. hamiltonii*, which might be employed for developing specific markers, phylogenetic, and ecological studies. The amino acid Leucine was prevalent, whereas codons encoding Arginine exhibited the largest and lowest usage bias. The second codon location in *F. hamiltonii* contains the maximum no. of RNA editing sites, indicating that RNA editing, particularly at the second codon position, might influence the encoding amino acid and the protein structure, which can be needed for protein job. The data of *F. hamiltonii* RNA editing sites, amino acid frequencies, codon use bias, simple sequence repeats, and oligonucleotide repeats were compared to those of *F. occidentalis* and found essentially identical findings with minor differences. The close similarity of the cp genome sequences of the two *Farsetia* species suggests that they are perfectly correlated. Furthermore, the phylogenetic tree exposed that the *Farsetia* genus was quite firmly connected to the *Morettia* genus, both of which are members of the Anastaticeae tribe of Clade C. The phylogenetic trees in this study further supported the genus *Farsetia*'s placement in Clade C by displaying considerable similarity to other members of the clade. We then compared *F. hamiltonii* to *F. occidentalis* and eight additional Clade C members, including *L. libyca, N. bicorne, P. ornata, M. canescens, C. borzaeana, M. polyandra, B. laevigata,* and *I. amara.* Compared to other clade C plants, it was discovered that the plastomes of clade C are highly conserved, with very small variations in genome content and structure. The remarkable polymorphic regions revealed in this study provide genetic data for creating molecular markers that may be utilized to evaluate phylogenetic connections. Such loci may also be beneficial in identifying the genetic diversity of the *Farsetia* genus. We attempted to contribute significantly to the extremely limited present understanding of the evolutionary dynamics of the *Farsetia* genus and clade C of the Brassicaceae family based on chloroplast genomes.

## Supplementary Information

**Additional file 1.** **Supplementary Table 1.** Genes containing introns and their length in *F. hamiltonii. ***Supplementary Table 2.** Frequency of Amino acid in F. hamiltonii and F.occidentalis. * shows the stop codon. **Supplementary Table 3.** Frequency of Amino acid and relative synonymous codon usagein *F. hamiltonii* (F.H) and *F. occidentalis *(F.O). * shows the stopcodons. **Supplementary Table 4.** RNA editing sites in *F.hamiltonii* and *F. occidentalis. ***Supplementary Table 5. **Simple sequence repeats types, size, and location in*F. hamiltonii*and*F. occidentalis. ***Supplementary Table 6.** Types and number of simple sequence repeats motifs in *F. hamiltonii *and *F. occidentalis. ***Supplementary Table 7.** Frequency of complementary simple sequence repeats in *F. hamiltonii *and *F. occidentalis. ***Supplementary Table 8.** Oligo-repeats analysis in F. hamiltonii and F.occidentalis. **Supplementary Table 9. **Farsetia hamiltonii, and 21 selected NCBI genomes of Brassicaceae familyfor Phylogenetic tree analysis. Two species of *Calotropis (Calotropis procera,Calotropis gigantea) *of family Apocynaceae were used as outgroup.**Supplementary Table 10. **Non-synonymous (Ka) and synonymous rate (Ks) ofsubstitution, Ka/Ks Value  in *F.hamiltonii *by making pairwise alignment with *Farsetia occidentalis* and eight other species of Clade C of Brassicaceae I.e. *Lobularia libyca, Notoceras bicorne, Paroliniaornata, Morettia canescens, Cochlearia borzaeana, Megacarpaea polyandra,Biscutella laevigata, and Iberis amara*. **Supplementary Table 11. **The detailedanalysis of InDels, Average InDel Length, InDel Diversity K(i), InDel Diversity persite Pi(i) and alignment length in LSC, IR and SSC regions of *F. hamiltonii *by making pairwise alignment with ninespecies of Clade C of Brassicaceae I.e. *Farsetia occidentalis*, *Lobularia libyca, Notoceras bicorne, Paroliniaornata, Morettia canescens, Cochlearia borzaeana, Megacarpaea polyandra,Biscutella laevigata, and Iberis amara*. **SupplementaryTable 12. **Singlenucleotide polymorphisms (SNPs) in LSC, IR and SSC regions of *F. hamiltonii *by making pairwise alignment with ninespecies of Clade C of Brassicaceae I.e. *Farsetia occidentalis*, *Lobularia libyca, Notoceras bicorne, Paroliniaornata, Morettia canescens, Cochlearia borzaeana, Megacarpaea polyandra,Biscutella laevigata, and Iberis amara*. **Supplementary Table 13. **NucleotideDiversity and Alignment Length incoding, non-coding, and intronic regions among *F. hamiltonii *andnine species ofClade C of Brassicaceae I.e. *Farsetia occidentalis*, *Lobularia libyca, Notoceras bicorne, Paroliniaornata, Morettia canescens, Cochlearia borzaeana, Megacarpaea polyandra,Biscutella laevigata, and Iberis amara. *

## Data Availability

The datasets generated and/or analysed during the current study are available in the NCBI repository, https://www.ncbi.nlm.nih.gov/nuccore/MT884003.1 / ACCESSION MT884003. All other data is provided in manuscript and supplymentry file.
