# Supplementary material for: The chloroplast genome of Farsetia hamiltonii Royle, phylogenetic analysis, and comparative study with other members of Clade C of Brassicaceae
Source: BMC Plant Biol. 2022 Aug 2;22:384. doi: 10.1186/s12870-022-03750-2 (PMC9344719; doi:10.1186/s12870-022-03750-2)
Supplement: Supplementary file 1 — Additional file 1. Supplementary Table 1. Genes containing introns and their length in F. hamiltonii. Supplementary Table 2. Frequency of Amino acid in F. hamiltonii and F.occidentalis. * shows the stop codon. Supplementary Table 3. Frequency of Amino acid and relative synonymous codon usagein F. hamiltonii (F.H) and F. occidentalis (F.O). * shows the stopcodons. Supplementary Table 4. RNA editing sites in F.hamiltonii and F. occidentalis. Supplementary Table 5. Simple sequence repeats types, size, and location inF. hamiltoniiandF. occidentalis. Supplementary Table 6. Types and number of simple sequence repeats motifs in F. hamiltonii and F. occidentalis. Supplementary Table 7. Frequency of complementary simple sequence repeats in F. hamiltonii and F. occidentalis. Supplementary Table 8. Oligo-repeats analysis in F. hamiltonii and F.occidentalis. Supplementary Table 9. Farsetia hamiltonii, and 21 selected NCBI genomes of Brassicaceae familyfor Phylogenetic tree analysis. Two species of Calotropis (Calotropis procera,Calotropis gigantea) of family Apocynaceae were used as outgroup.Supplementary Table 10. Non-synonymous (Ka) and synonymous rate (Ks) ofsubstitution, Ka/Ks Value in F.hamiltonii by making pairwise alignment with Farsetia occidentalis and eight other species of Clade C of Brassicaceae I.e. Lobularia libyca, Notoceras bicorne, Paroliniaornata, Morettia canescens, Cochlearia borzaeana, Megacarpaea polyandra,Biscutella laevigata, and Iberis amara. Supplementary Table 11. The detailedanalysis of InDels, Average InDel Length, InDel Diversity K(i), InDel Diversity persite Pi(i) and alignment length in LSC, IR and SSC regions of F. hamiltonii by making pairwise alignment with ninespecies of Clade C of Brassicaceae I.e. Farsetia occidentalis, Lobularia libyca, Notoceras bicorne, Paroliniaornata, Morettia canescens, Cochlearia borzaeana, Megacarpaea polyandra,Biscutella laevigata, and Iberis amara. SupplementaryTable 12. Singlenucleotide polymorphisms (SNPs) in LS [file 12870_2022_3750_MOESM1_ESM.docx]

**The chloroplast genome of *Farsetia hamiltonii* Royle*,* phylogenetic analysis, and comparative study with other members of Clade C of Brassicaceae**

Nida Javaid1, Musarrat Ramzan1*, Ishtiaq Ahmad Khan2, Tahani Awad Alahmadi3, Rahul Datta4, Shah Fahad5,6*, Subhan Danish6,7*

^1^The Islamia University Bahawalpur, Pakistan; nidajatala1@gmail.com; musarrat.ramzan@iub.edu.pk

^2^Jamil-ur-Rehman Center for Genome Research, International Center for Chemical and Biological Sciences, University of Karachi, Karachi, 75270, Pakistan; ishtiaq.ahmed@iccs.edu

^3^Department of Pediatrics, College of Medicine and King Khalid University Hospital, King Saud University, Medical City, PO Box-2925, Riyadh -11461, Saudi Arabia; talahmadi@ksu.edu.sa

^4^Department of Geology and Pedology, Faculty of Forestry and Wood Technology, Mendel University in Brno, Zemedelska1, 61300 Brno, Czech Republic; rahulmedcure@gmail.com

^5^Department of Agronomy, The University of Haripur, Khyber Pakhtunkhwa 22620, Pakistan; shah_fahad80@yahoo.com

^6^Hainan Key Laboratory for Sustainable Utilization of Tropical Bioresource, College of Tropical Crops, Hainan University, Haikou 570228, China

^7^Department of Soil Science, Faculty of Agricultural Sciences and Technology, Bahauddin Zakariya University, Multan, Punjab 60800, Pakistan; sd96850@gmail.com; [niaz.ahmad@bzu.edu.pk](mailto:niaz.ahmad@bzu.edu.pk)

**Supplementary table 1:** Genes containing introns and their length in *F. hamiltonii.*

| **Genes** | **Strand** | **Gene length** | | **Length** | **Exon I** | **Intron I** | **Exon II** | **Intron II** | **Exon III** |
| --- | --- | --- | --- | --- | --- | --- | --- | --- | --- |
|  |  | **Start** | **End** |  |  |  |  |  |  |
| ***Farsetia hamiltonii*** | | | | | | | | | |
| *clpP* | reverse | 69437 | 71554 | 2118 | 71 | 943 | 292 | 584 | 228 |
| *ycf3* | reverse | 42032 | 44077 | 2046 | 126 | 715 | 228 | 824 | 153 |
| *trnI-AUC* | reverse | 135214 | 136232 | 1019 | 37 | 947 | 35 |  |  |
| *trnA-GCA* | reverse | 134277 | 135149 | 873 | 38 | 800 | 35 |  |  |
| *trnA-GCA* | forward | 103560 | 104432 | 873 | 38 | 800 | 35 |  |  |
| *trnI-AUC* | forward | 102477 | 103495 | 1019 | 37 | 947 | 35 |  |  |
| *trnV-GUA* | reverse | 50532 | 51211 | 680 | 39 | 606 | 35 |  |  |
| *trnL-UUA* | forward | 46544 | 46949 | 406 | 35 | 321 | 50 |  |  |
| *trnG-GGA* | forward | 8610 | 9403 | 794 | 23 | 722 | 49 |  |  |
| *trnK-AAA* | reverse | 1700 | 4325 | 2626 | 37 | 2554 | 35 |  |  |
| *rpl2* | forward | 153128 | 154634 | 1507 | 435 | 682 | 390 |  |  |
| *ndhB* | forward | 141792 | 144015 | 2224 | 777 | 685 | 762 |  |  |
| *ndhA* | reverse | 120002 | 122173 | 2172 | 553 | 1089 | 530 |  |  |
| *ndhB* | reverse | 94694 | 96917 | 2224 | 777 | 685 | 762 |  |  |
| *rpl2* | reverse | 84075 | 85581 | 1507 | 435 | 682 | 390 |  |  |
| *rpl16* | reverse | 80868 | 82373 | 408 | 40 | 895 | 227 |  |  |
| *petD* | forward | 76157 | 77344 | 1188 | 8 | 705 | 475 |  |  |
| *petB* | forward | 74518 | 75962 | 1445 | 6 | 797 | 642 |  |  |
| *rpoC1* | reverse | 20264 | 23132 | 2869 | 432 | 826 | 1611 |  |  |
| *atpF* | reverse | 11531 | 12809 | 1279 | 145 | 724 | 410 |  |  |
| *rps16* | reverse | 4983 | 6144 | 1162 | 40 | 895 | 227 |  |  |

**Supplementary table 2:** Frequency of Amino acid in *F. hamiltonii* and *F. occidentalis*. * shows the stop codon.

| ***F. hamiltonii*** | | | ***F. occidentalis*** | | |
| --- | --- | --- | --- | --- | --- |
| **A.A** | **Frequency** | **%** | **A.A** | **Frequency** | **%** |
| A | 2,637 | 5.10% | A | 1,374 | 5.20% |
| C | 654 | 1.30% | C | 315 | 1.20% |
| D | 2,071 | 4.00% | D | 1,033 | 3.90% |
| E | 2,781 | 5.30% | E | 1,385 | 5.30% |
| F | 3,215 | 6.20% | F | 1,585 | 6.00% |
| G | 3,452 | 6.60% | G | 1,760 | 6.70% |
| H | 1,211 | 2.30% | H | 615 | 2.30% |
| I | 4,486 | 8.60% | I | 2,271 | 8.60% |
| K | 2,985 | 5.70% | K | 1,524 | 5.80% |
| L | 5,488 | 10.60% | L | 2,802 | 10.60% |
| M | 1,201 | 2.30% | M | 596 | 2.30% |
| N | 2,558 | 4.90% | N | 1,296 | 4.90% |
| P | 2,089 | 4.00% | P | 1,060 | 4.00% |
| Q | 1,831 | 3.50% | Q | 940 | 3.60% |
| R | 3,025 | 5.80% | R | 1,568 | 5.90% |
| S | 4,026 | 7.70% | S | 2,017 | 7.60% |
| T | 2,615 | 5.00% | T | 1,344 | 5.10% |
| V | 2,726 | 5.20% | V | 1,404 | 5.30% |
| W | 889 | 1.70% | W | 443 | 1.70% |
| Y | 1,902 | 3.70% | Y | 959 | 3.60% |
| * | 149 | 0.30% | * | 87 | 0.30% |

**Supplementary table 3:** Frequency of Amino acid and relative synonymous codon usage in *F. hamiltonii* (F.H) and *F. occidentalis* (F.O). * shows the stop codons.

| **Codon** | **AA** | **Freq of A.Acid** | | **RSCU** | | **Codon** | **AA** | **Freq of A.Acid** | | **RSCU** | |
| --- | --- | --- | --- | --- | --- | --- | --- | --- | --- | --- | --- |
|  |  | **F.H** | **F.O** | **F.H** | **F.O** |  |  | **F.H** | **F.O** | **F.H** | **F.O** |
| GCA | A | 731 | 385 | 1.18 | 1.12 | TTG | M | 9 |  |  |  |
| GCC | A | 419 | 206 | 0.84 | 0.92 | AAC | N | 609 | 310 | 0.61 | 0.61 |
| GCG | A | 289 | 145 | 0.62 | 0.70 | AAT | N | 1,949 | 986 | 1.39 | 1.39 |
| GCT | A | 1,198 | 638 | 1.36 | 1.26 | CCA | P | 584 | 304 | 1.22 | 1.21 |
| TGC | C | 161 | 80 | 0.78 | 0.80 | CCC | P | 396 | 199 | 1.00 | 1.01 |
| TGT | C | 493 | 235 | 1.22 | 1.20 | CCG | P | 276 | 140 | 0.65 | 0.64 |
| GAC | D | 393 | 192 | 0.53 | 0.61 | CCT | P | 833 | 417 | 1.13 | 1.14 |
| GAT | D | 1,678 | 841 | 1.47 | 1.39 | CAA | Q | 1,412 | 727 | 1.37 | 1.38 |
| GAA | E | 2,095 | 1,049 | 1.39 | 1.41 | CAG | Q | 419 | 213 | 0.63 | 0.62 |
| GAG | E | 686 | 336 | 0.61 | 0.59 | AGA | R | 902 | 471 | 1.98 | 2.01 |
| TTC | F | 1,068 | 511 | 0.70 | 0.75 | AGG | R | 330 | 164 | 0.98 | 1.13 |
| TTT | F | 2,147 | 1,074 | 1.30 | 1.25 | CGA | R | 685 | 363 | 1.09 | 0.96 |
| GGA | G | 1,417 | 733 | 1.44 | 1.38 | CGC | R | 225 | 114 | 0.45 | 0.51 |
| GGC | G | 349 | 168 | 0.59 | 0.63 | CGG | R | 236 | 116 | 0.73 | 0.70 |
| GGG | G | 542 | 277 | 0.93 | 0.94 | CGT | R | 647 | 340 | 0.77 | 0.69 |
| GGT | G | 1,144 | 582 | 1.04 | 1.06 | AGC | S | 259 | 117 | 0.58 | 0.63 |
| CAC | H | 298 | 146 | 0.61 | 0.58 | AGT | S | 767 | 401 | 0.92 | 0.92 |
| CAT | H | 913 | 469 | 1.39 | 1.42 | TCA | S | 825 | 406 | 1.26 | 1.17 |
| ATA | I | 1,437 | 732 | 1.07 | 1.07 | TCC | S | 596 | 296 | 1.06 | 1.14 |
| ATC | I | 820 | 409 | 0.66 | 0.72 | TCG | S | 405 | 199 | 0.73 | 0.71 |
| ATT | I | 2,229 | 1,130 | 1.26 | 1.20 | TCT | S | 1,174 | 598 | 1.46 | 1.42 |
| AAA | K | 2,265 | 1,164 | 1.40 | 1.39 | ACA | T | 810 | 414 | 1.18 | 1.13 |
| AAG | K | 720 | 360 | 0.60 | 0.61 | ACC | T | 468 | 237 | 0.94 | 1.05 |
| CTA | L | 778 | 390 | 1.00 | 0.94 | ACG | T | 267 | 132 | 0.59 | 0.68 |
| CTC | L | 360 | 179 | 0.69 | 0.72 | ACT | T | 1,070 | 561 | 1.28 | 1.14 |
| CTG | L | 342 | 174 | 0.51 | 0.52 | GTA | V | 978 | 503 | 1.23 | 1.20 |
| CTT | L | 1,151 | 584 | 1.31 | 1.21 | GTC | V | 338 | 174 | 0.67 | 0.72 |
| TTA | L | 1,822 | 940 | 1.35 | 1.39 | GTG | V | 393 | 200 | 0.70 | 0.72 |
| TTG | L | 1,035 | 535 | 1.15 | 1.22 | GTT | V | 1,017 | 527 | 1.40 | 1.36 |
| ATA | M | 8 |  |  |  | TGG | W | 889 | 443 | 1.00 | 1.00 |
| ATC | M | 11 |  |  |  | TAC | Y | 369 | 177 | 0.59 | 0.66 |
| ATG | M | 1,149 | 591 | 1.00 | 1.00 | TAT | Y | 1,533 | 782 | 1.41 | 1.34 |
| ATT | M | 13 |  |  |  | TAA | * | 89 | 51 | 1.31 | 1.27 |
| CTG | M | 2 |  |  |  | TAG | * | 35 | 24 | 0.78 | 0.79 |
| GTG | M | 9 | 5 |  |  | TGA | * | 25 | 12 | 0.91 | 0.94 |

**Supplementary table 4**: RNA editing sites in *F. hamiltonii* and *F. occidentalis.*

| ***Farsetia hamiltonii*** | | | | | **GENE NAME** | ***Farsetia occidentalis*** | | | | |
| --- | --- | --- | --- | --- | --- | --- | --- | --- | --- | --- |
| **Nt Pos** | **AA Pos** | **Align Col** | **Effect** | **Score** |  | **Nt Pos** | **AA Pos** | **Align Col** | **Effect** | **Score** |
| 742 | 248 | 275 | CAT (H) =>TAT (Y) | 0.8 | ***accD*** | 742 | 248 | 275 | CAT (H) =>TAT (Y) | 0.8 |
| 715 | 239 | 249 | CAT (H) =>TAT (Y) | 1 | ***matK*** | 715 | 239 | 249 | CAT (H) =>TAT (Y) | 1 |
| 1259 | 420 | 430 | TCA (S) =>TTA (L) | 0.86 |  | 1259 | 420 | 430 | TCA (S) => TTA (L) | 0.86 |
| 323 | 108 | 116 | ACC (T) => ATC (I) | 1 | ***ndhA*** | 323 | 108 | 116 | ACC (T) => ATC (I) | 1 |
| 938 | 313 | 321 | CCA (P) => CTA (L) | 1 |  | 938 | 313 | 322 | CCA (P) => CTA (L) | 1 |
| 956 | 319 | 327 | ACC (T) => ATC (I) | 1 |  | 956 | 319 | 328 | ACC (T) => ATC (I) | 1 |
| 1001 | 334 | 342 | CCA (P) => CTA (L) | 1 |  | 1001 | 334 | 343 | CCA (P) => CTA (L) | 1 |
| 1018 | 340 | 348 | CTC (L) =>TTC (F) | 1 |  | 1018 | 340 | 349 | CTC (L) =>TTC (F) | 1 |
| 116 | 39 | 39 | TCT (S) => TTT (F) | 1 | ***ndhB*** | 98 | 33 | 156 | CCA (P) => CTA (L) | 1 |
| 167 | 56 | 56 | TCG (S) => TTG (L) | 1 |  | 217 | 73 | 196 | CAT (H) =>TAT (Y) | 1 |
| 221 | 74 | 75 | GCT (A) => GTT (V) | 1 |  | 377 | 126 | 249 | TCT (S) => TTT (F) | 1 |
| 274 | 92 | 94 | CCA (P) =>TCA (S) | 1 |  | 461 | 154 | 277 | TCA (S) => TTA (L) | 1 |
| 491 | 164 | 166 | GCT (A) => GTT (V) | 1 |  | 886 | 296 | 419 | CAT (H) =>TAT (Y) | 1 |
| 611 | 204 | 206 | CCC (P) => CTC (L) | 1 |  | 1112 | 371 | 494 | CCA (P) => CTA (L) | 1 |
| 764 | 255 | 261 | TCC (S) => TTC (F) | 1 |  |  |  |  |  |  |
| 923 | 308 | 317 | CCC (P) => CTC (L) | 1 |  |  |  |  |  |  |
| 932 | 311 | 320 | ACC (T) => ATC (I) | 1 |  |  |  |  |  |  |
| 1274 | 425 | 434 | TCC (S) => TTC (F) | 1 |  |  |  |  |  |  |
| 1288 | 430 | 439 | CAT (H) =>TAT (Y) | 1 |  |  |  |  |  |  |
| 1340 | 447 | 456 | ACT (T) => ATT (I) | 1 |  |  |  |  |  |  |
| 1363 | 455 | 464 | CCA (P) =>TCA (S) | 1 |  |  |  |  |  |  |
| 1495 | 499 | 508 | CGT (R) =>TGT (C) | 1 |  |  |  |  |  |  |
| 20 | 7 | 7 | ACG (T) => ATG (M) | 1 | ***ndhD*** | 20 | 7 | 7 | ACG (T) => ATG (M) | 1 |
| 65 | 22 | 22 | TCT (S) => TTT (F) | 0.8 |  | 65 | 22 | 22 | TCT (S) => TTT (F) | 0.8 |
| 692 | 231 | 231 | TCA (S) => TTA (L) | 1 |  | 692 | 231 | 231 | TCA (S) => TTA (L) | 1 |
| 896 | 299 | 299 | TCA (S) => TTA (L) | 1 |  | 896 | 299 | 299 | TCA (S) => TTA (L) | 1 |
| 905 | 302 | 302 | CCC (P) => CTC (L) | 1 |  | 905 | 302 | 302 | CCC (P) => CTC (L) | 1 |
| 1328 | 443 | 443 | TCA (S) => TTA (L) | 0.8 |  | 1328 | 443 | 443 | TCA (S) => TTA (L) | 0.8 |
| 1423 | 475 | 475 | CTT (L) =>TTT (F) | 0.8 |  | 1415 | 472 | 472 | ACC (T) => ATC (I) | 1 |
|  |  |  |  |  |  | 1423 | 475 | 475 | CTT (L) =>TTT (F) | 0.8 |
| 205 | 69 | 69 | CAT (H) =>TAT (Y) | 0.8 | ***ndhF*** | 205 | 69 | 69 | CAT (H) =>TAT (Y) | 0.8 |
| 290 | 97 | 97 | TCA (S) => TTA (L) | 1 |  | 290 | 97 | 97 | TCA (S) => TTA (L) | 1 |
| 586 | 196 | 196 | CTT (L) =>TTT (F) | 0.8 |  | 586 | 196 | 196 | CTT (L) =>TTT (F) | 0.8 |
| 2135 | 712 | 719 | ACA (T) => ATA (I) | 0.8 |  | 2135 | 712 | 719 | ACA (T) => ATA (I) | 0.8 |
| 166 | 56 | 56 | CAT (H) =>TAT (Y) | 0.8 | ***ndhG*** | 166 | 56 | 56 | CAT (H) =>TAT (Y) | 0.8 |
| 314 | 105 | 105 | ACA (T) => ATA (I) | 0.8 |  | 314 | 105 | 105 | ACA (T) => ATA (I) | 0.8 |
| 305 | 102 | 102 | GCT (A) => GTT (V) | 0.86 | ***petD*** | 305 | 102 | 102 | GCT (A) => GTT (V) | 0.86 |
| 14 | 5 | 5 | TCT (S) => TTT (F) | 0.86 | ***petG*** | 14 | 5 | 5 | TCT (S) => TTT (F) | 0.86 |
| 214 | 72 | 72 | CCT (P) =>TCT (S) | 1 | ***psbE*** | 214 | 72 | 72 | CCT (P) =>TCT (S) | 1 |
| 77 | 26 | 26 | TCT (S) => TTT (F) | 1 | ***psbF*** | 77 | 26 | 26 | TCT (S) => TTT (F) | 1 |
| 221 | 74 | 77 | ACT (T) => ATT (I) | 1 | ***rpl2*** | **N/A** | | | | |
| 247 | 83 | 86 | CAC (H) =>TAC (Y) | 1 |  |  |  |  |  |  |
| 338 | 113 | 113 | TCT (S) => TTT (F) | 1 | ***rpoB*** | 338 | 113 | 113 | TCT (S) => TTT (F) | 1 |
| 551 | 184 | 185 | TCA (S) => TTA (L) | 1 |  | 551 | 184 | 185 | TCA (S) => TTA (L) | 1 |
| 566 | 189 | 190 | TCG (S) => TTG (L) | 1 |  | 566 | 189 | 190 | TCG (S) => TTG (L) | 1 |
| 973 | 325 | 332 | CTC (L) =>TTC (F) | 0.86 |  | 973 | 325 | 332 | CTC (L) =>TTC (F) | 0.86 |
| 2432 | 811 | 827 | TCA (S) => TTA (L) | 0.86 |  | 2432 | 811 | 827 | TCA (S) => TTA (L) | 0.86 |
| 74 | 25 | 27 | ACC (T) => ATC (I) | 0.86 | ***rpoC1*** | 74 | 25 | 27 | ACC (T) => ATC (I) | 0.86 |
| 122 | 41 | 43 | TCT (S) => TTT (F) | 0.86 |  | 122 | 41 | 43 | TCT (S) => TTT (F) | 0.86 |
| 316 | 106 | 109 | CAT (H) =>TAT (Y) | 1 |  | 316 | 106 | 109 | CAT (H) =>TAT (Y) | 1 |
| 365 | 122 | 125 | TCG (S) => TTG (L) | 1 |  | 365 | 122 | 125 | TCG (S) => TTG (L) | 1 |
| 401 | 134 | 137 | TCG (S) => TTG (L) | 0.86 |  | 401 | 134 | 137 | TCG (S) => TTG (L) | 0.86 |
| 520 | 174 | 182 | CTC (L) =>TTC (F) | 0.86 |  | 520 | 174 | 182 | CTC (L) =>TTC (F) | 0.86 |
| 739 | 247 | 283 | CGG (R) =>TGG (W) | 1 |  | 739 | 247 | 283 | CGG (R) =>TGG (W) | 1 |
| 1090 | 364 | 402 | CCC (P) =>TCC (S) | 1 |  | 1090 | 364 | 402 | CCC (P) =>TCC (S) | 1 |
| 1178 | 393 | 434 | ACT (T) => ATT (I) | 1 |  | 1106 | 369 | 407 | ACT (T) => ATT (I) | 1 |
| 1190 | 397 | 438 | ACC (T) => ATC (I) | 1 |  | 1178 | 393 | 434 | ACT (T) => ATT (I) | 1 |
| 1358 | 453 | 495 | CCC (P) => CTC (L) | 1 |  | 1190 | 397 | 438 | ACC (T) => ATC (I) | 1 |
| 1475 | 492 | 537 | TCG (S) => TTG (L) | 1 |  | 1358 | 453 | 495 | CCC (P) => CTC (L) | 1 |
| 1732 | 578 | 623 | CAT (H) =>TAT (Y) | 0.86 |  | 1475 | 492 | 537 | TCG (S) => TTG (L) | 1 |
| 1792 | 598 | 643 | CGG (R) =>TGG (W) | 1 |  | 1732 | 578 | 623 | CAT (H) =>TAT (Y) | 0.86 |
| 1858 | 620 | 669 | CCC (P) =>TCC (S) | 1 |  | 1792 | 598 | 643 | CGG (R) =>TGG (W) | 1 |
| 1933 | 645 | 694 | CAC (H) =>TAC (Y) | 1 |  | 1858 | 620 | 669 | CCC (P) =>TCC (S) | 1 |
| 2032 | 678 | 727 | CCG (P) =>TCG (S) | 0.86 |  | 1933 | 645 | 694 | CAC (H) =>TAC (Y) | 1 |
|  |  |  |  |  |  | 2032 | 678 | 727 | CCG (P) =>TCG (S) | 0.86 |
| 2293 | 765 | 948 | CGG (R) =>TGG (W) | 1 | ***rpoC2*** | 2251 | 751 | 934 | CCT (P) =>TCT (S) | 1 |
| 2342 | 781 | 968 | GCC (A) => GTC (V) | 0.86 |  | 2293 | 765 | 948 | CGG (R) =>TGG (W) | 1 |
| 3083 | 1028 | 1235 | ACT (T) => ATT (I) | 0.86 |  | 2342 | 781 | 968 | GCC (A) => GTC (V) | 0.86 |
|  |  |  |  |  |  | 3083 | 1028 | 1235 | ACT (T) => ATT (I) | 0.86 |
| 80 | 27 | 27 | TCA (S) => TTA (L) | 1 | ***rps14*** | 80 | 27 | 27 | TCA (S) => TTA (L) | 1 |
| 149 | 50 | 53 | CCA (P) => CTA (L) | 1 |  | 149 | 50 | 53 | CCA (P) => CTA (L) | 1 |
| 109 | 37 | 37 | CTC (L) =>TTC (F) | 1 | ***ycf3*** | 152 | 51 | 62 | ACG (T) => ATG (M) | 1 |
| 115 | 39 | 39 | CAT (H) =>TAT (Y) | 1 |  | 172 | 58 | 73 | CAT (H) =>TAT (Y) | 1 |
| 217 | 73 | 73 | CCG (P) =>TCG (S) | 1 |  | 218 | 73 | 92 | CCG (P) => CTG (L) | 1 |

**Supplementary table 5**: Simple sequence repeats types, size, and location in *F. hamiltonii* and

*F. occidentalis.*

| **SSR nr.** | **SSR type** | **SSR** | **Size** | **Start** | **End** | **Region** | **Locus** | **Location** |
| --- | --- | --- | --- | --- | --- | --- | --- | --- |
| ***Farsetia hamiltonii*** | | | | | | | | |
| 1 | p1 | (T)10 | 10 | 1585 | 1594 | LSC | IGS | *psbA-trnk-AAA* |
| 2 | p1 | (A)12 | 12 | 1764 | 1775 | LSC | Intron | *trnk-AAA* |
| 3 | p2 | (AT)5 | 10 | 3797 | 3806 | LSC | Intron | *trnk-AAA* |
| 4 | p1 | (T)10 | 10 | 4013 | 4022 | LSC | Intron | *trnk-AAA* |
| 5 | p1 | (A)16 | 16 | 4375 | 4390 | LSC | IGS | *trnk-AAA-rps16* |
| 6 | p2 | (AT)5 | 10 | 4678 | 4687 | LSC | IGS | *trnk-AAA-rps16* |
| 7 | p2 | (TA)5 | 10 | 6357 | 6366 | LSC | IGS | *rps16-trnQ-CAA* |
| 8 | c | (T)10g(A)10 | 21 | 7610 | 7630 | LSC | IGS | *psbK-psbI* |
| 9 | c | (A)14(TTTA)3 | 39 | 7762 | 7800 | LSC | CDS/IGS | *psbI/trnS-AGC* |
| 10 | p2 | (AT)5 | 10 | 8131 | 8140 | LSC | IGS | *trnS-AGC-trnG-GGA* |
| 11 | c | (T)11(A)11(T)11 | 143 | 8396 | 8538 | LSC | IGS | *trnS-AGC-trnG-GGA* |
| 12 | p1 | (C)10 | 10 | 9419 | 9428 | LSC | IGS | *trnG-GGA-trnR-AGA* |
| 13 | p1 | (T)10 | 10 | 9538 | 9547 | LSC | IGS | *trnG-GGA-trnR-AGA* |
| 14 | c | (TA)5(CTTTT)3 | 123 | 9818 | 9940 | LSC | IGS | *trnR-AGA-atpA* |
| 15 | p3 | (AAT)4 | 12 | 12994 | 13005 | LSC | IGS | *atpF-atpH* |
| 16 | p2 | (AT)7 | 14 | 13730 | 13743 | LSC | IGS | *atpH-atpI* |
| 17 | p1 | (A)10 | 10 | 14010 | 14019 | LSC | IGS | *atpH-atpI* |
| 18 | p4 | (ATTT)3 | 12 | 14989 | 15000 | LSC | IGS | *atpI-rps2* |
| 19 | p1 | (T)11 | 11 | 17939 | 17949 | LSC | CDS | *rpoC2* |
| 20 | p2 | (TA)5 | 10 | 19311 | 19320 | LSC | CDS | *rpoC2* |
| 21 | p1 | (T)14 | 14 | 22505 | 22518 | LSC | Intron | *rpoC1* |
| 22 | p1 | (T)10 | 10 | 25732 | 25741 | LSC | CDS | *rpoB* |
| 23 | p1 | (A)11 | 11 | 26580 | 26590 | LSC | IGS | *rpoB-trnC-UGC* |
| 24 | p4 | (CAAA)3 | 12 | 28473 | 28484 | LSC | IGS | *petN-psbM* |
| 25 | c | (TTA)4(T)13 | 25 | 28889 | 28913 | LSC | IGS | *psbM-trnD-GAC* |
| 26 | p2 | (TA)6 | 12 | 29579 | 29590 | LSC | IGS | *psbM-trnD-GAC* |
| 27 | p1 | (A)13 | 13 | 31850 | 31862 | LSC | IGS | *trnT-ACC-psbD* |
| 28 | p2 | (AT)5 | 10 | 35775 | 35784 | LSC | IGS | *psbZ-trnG-GGC* |
| 29 | p1 | (T)14 | 14 | 41528 | 41541 | LSC | IGS | *psaA-ycf3* |
| 30 | p1 | (A)11 | 11 | 42003 | 42013 | LSC | IGS | *psaA-ycf3* |
| 31 | p1 | (A)10 | 10 | 43924 | 43933 | LSC | Intron | *ycf3* |
| 32 | p1 | (A)18 | 18 | 47570 | 47587 | LSC | IGS | *trnF-UUC-ndhJ* |
| 33 | p4 | (AAAT)3 | 12 | 49991 | 50002 | LSC | IGS | *ndhC-trnV-GUA* |
| 34 | p1 | (T)10 | 10 | 53386 | 53395 | LSC | CDS | *atpB* |
| 35 | c* | (TAAA)2(T<AAA>...TTTCTCG | 22 | 55858 | 55879 | LSC | IGS | *rbcL-accD* |
| 36 | p1 | (T)10 | 10 | 56739 | 56748 | LSC | CDS | *accD* |
| 37 | p2 | (AT)5 | 10 | 59071 | 59080 | LSC | IGS/CDS | *psaI-ycf4/ycf4* |
| 38 | p1 | (T)10 | 10 | 59699 | 59708 | LSC | IGS | *ycf4-cemA* |
| 39 | c | (TA)5(TA)5(AT)6 | 75 | 62509 | 62583 | LSC | IGS | *petA-psbJ* |
| 40 | p4 | (TAAA)3 | 12 | 62692 | 62703 | LSC | IGS | *petA-psbJ* |
| 41 | p1 | (T)12 | 12 | 64023 | 64034 | LSC | IGS | *psbE-petL* |
| 42 | p3 | (AAT)5 | 15 | 64395 | 64409 | LSC | IGS | *psbE-petL* |
| 43 | p4 | (AAAT)3 | 12 | 64628 | 64639 | LSC | IGS | *psbE-petL* |
| 44 | p1 | (A)14 | 14 | 64806 | 64819 | LSC | IGS | *psbE-petL* |
| 45 | p1 | (T)13 | 13 | 66169 | 66181 | LSC | IGS | *trnP-CCA-psaJ* |
| 46 | p2 | (AT)6 | 12 | 67263 | 67274 | LSC | IGS | *rpl33-rps18* |
| 47 | p1 | (A)12 | 12 | 67848 | 67859 | LSC | IGS | *rps18-rpl20* |
| 48 | p1 | (A)10 | 10 | 68473 | 68482 | LSC | IGS | *rpl20-rps12* |
| 49 | p1 | (A)10 | 10 | 69724 | 69733 | LSC | Intron | *clpP* |
| 50 | p1 | (T)14 | 14 | 69976 | 69989 | LSC | Intron | *clpP* |
| 51 | p1 | (T)15 | 15 | 70980 | 70994 | LSC | Intron | *clpP* |
| 52 | p1 | (T)10 | 10 | 74818 | 74827 | LSC | Intron | *petB* |
| 53 | p1 | (A)13 | 13 | 76411 | 76423 | LSC | Intron | *petD* |
| 54 | p2 | (AT)5 | 10 | 76749 | 76758 | LSC | Intron | *petD* |
| 55 | p1 | (T)13 | 13 | 77732 | 77744 | LSC | CDS | *rpoA* |
| 56 | p2 | (TA)6 | 12 | 80196 | 80207 | LSC | IGS | *rps8-rpl14* |
| 57 | c | (T)11(A)11 | 48 | 81592 | 81639 | LSC | Intron | *rpl16* |
| 58 | p1 | (T)10 | 10 | 82317 | 82326 | LSC | Intron | *rpl16* |
| 59 | p6 | (GGAGCT)3 | 18 | 91344 | 91361 | IRB | CDS | *ycf2* |
| 60 | p2 | (TA)5 | 10 | 94276 | 94285 | IRB | IGS | *trnL-UUG-ndhB* |
| 61 | p1 | (A)10 | 10 | 96943 | 96952 | IRB | IGS | *ndhB-rps7* |
| 62 | p1 | (A)10 | 10 | 99117 | 99126 | IRB | IGS | *rps12-trnV-GUC* |
| 63 | p1 | (A)12 | 12 | 108009 | 108020 | IRB | IGS | *rrn55-trnR-CGU* |
| 64 | p1 | (A)10 | 10 | 110341 | 110350 | IRB | CDS | *ycf1* |
| 65 | p4 | (ATAG)3 | 12 | 112386 | 112397 | SSC | CDS | *ndhF* |
| 66 | p2 | (TA)5 | 10 | 112630 | 112639 | SSC | IGS | *ndhF-rpl32* |
| 67 | c | (TA)5(A)10 | 48 | 112969 | 113016 | SSC | IGS | *ndhF-rpl32* |
| 68 | p3 | (AAT)4 | 12 | 113333 | 113344 | SSC | IGS | *ndhF-rpl32* |
| 69 | p1 | (T)13 | 13 | 113754 | 113766 | SSC | IGS | *rpl32-trnL-CUA* |
| 70 | p4 | (AATT)3 | 12 | 113878 | 113889 | SSC | IGS | *rpl32-trnL-CUA* |
| 71 | p3 | (ATA)4 | 12 | 114017 | 114028 | SSC | IGS | *rpl32-trnL-CUA* |
| 72 | p1 | (A)14 | 14 | 114906 | 114919 | SSC | CDS | *ccsA* |
| 73 | p1 | (A)11 | 11 | 117448 | 117458 | SSC | IGS | *ndhD-psaC* |
| 74 | p1 | (A)11 | 11 | 121007 | 121017 | SSC | Intron | *ndhA* |
| 75 | p2 | (AT)5 | 10 | 121532 | 121541 | SSC | Intron | *ndhA* |
| 76 | c | (T)12(T)10(T)16 | 152 | 124298 | 124449 | SSC | CDS | *ycf1* |
| 77 | p1 | (T)11 | 11 | 125981 | 125991 | SSC | CDS | *ycf1* |
| 78 | p1 | (A)10 | 10 | 126142 | 126151 | SSC | CDS | *ycf1* |
| 79 | c | (TTC)4(T)11 | 67 | 127138 | 127204 | SSC | CDS | *ycf1* |
| 80 | p1 | (T)10 | 10 | 128359 | 128368 | IRA | CDS | *ycf1* |
| 81 | p1 | (T)12 | 12 | 130689 | 130700 | IRA | IGS | *trnR-CGU-rrn5S* |
| 82 | p1 | (T)10 | 10 | 139583 | 139592 | IRA | IGS | *trnV-GUC-rps12* |
| 83 | p1 | (T)10 | 10 | 141757 | 141766 | IRA | IGS | *rps7-ndhB* |
| 84 | p2 | (AT)5 | 10 | 144423 | 144432 | IRA | IGS | *ndhB-trnL-UUG* |
| 85 | p6 | (AGCTCC)3 | 18 | 147348 | 147365 | IRA | CDS | *ycf1* |
| ***Farsetia occidentalis*** | | | | | | | | |
| **SSR nr.** | **SSR type** | **SSR** | **Size** | **Start** | **End** | **Region** | **Locus** | **Location** |
| 1 | p1 | (T)10 | 10 | 1589 | 1598 | LSC | IGS | *psbA-trnk-AAA* |
| 2 | p2 | (AT)5 | 10 | 3797 | 3806 | LSC | Intron | *trnK-AAA* |
| 3 | p1 | (A)14 | 14 | 4346 | 4359 | LSC | IGS | *trnk-AAA-rps16* |
| 4 | p2 | (AT)5 | 10 | 4647 | 4656 | LSC | IGS | *trnk-AAA-rps16* |
| 5 | c | (TA)5a(AT)5 | 21 | 6343 | 6363 | LSC | IGS | *rps16-trnQ-CAA* |
| 6 | p4 | (TTTA)3 | 12 | 7767 | 7778 | LSC | IGS | *psbI-trnS-AGC* |
| 7 | c | (T)10(TA)6(AT)5 | 77 | 8062 | 8138 | LSC | IGS | *trnS-AGC-trnG-GGA* |
| 8 | p1 | (T)10 | 10 | 8522 | 8531 | LSC | IGS | *trnS-AGC-trnG-GGA* |
| 9 | p1 | (T)13 | 13 | 9516 | 9528 | LSC | IGS | *trnG-GGA-trnR-AGA* |
| 10 | p3 | (AAT)4 | 12 | 12974 | 12985 | LSC | IGS | *atpF-atpH* |
| 11 | p2 | (AT)6 | 12 | 13715 | 13726 | LSC | IGS | *atpH-atpI* |
| 12 | p1 | (T)11 | 11 | 17920 | 17930 | LSC | CDS | *rpoC2* |
| 13 | p2 | (TA)5 | 10 | 19292 | 19301 | LSC | CDS | *rpoC2* |
| 14 | p1 | (T)11 | 11 | 22487 | 22497 | LSC | Intron | *rpoC1* |
| 15 | p1 | (T)10 | 10 | 25711 | 25720 | LSC | CDS | *rpoB* |
| 16 | p1 | (T)11 | 11 | 27453 | 27463 | LSC | IGS | *rpoB-trnC-UGC* |
| 17 | p4 | (CAAA)3 | 12 | 28549 | 28560 | LSC | IGS | *petN-psbM* |
| 18 | c | (TTA)4(T)10 | 22 | 28966 | 28987 | LSC | IGS | *psbM-trnD-GAC* |
| 19 | p2 | (AT)5 | 10 | 31147 | 31156 | LSC | IGS | *trnE-GAA-trnT-ACC* |
| 20 | p1 | (A)10 | 10 | 31981 | 31990 | LSC | IGS | *trnT-ACC-psbD* |
| 21 | p2 | (AT)5 | 10 | 35900 | 35909 | LSC | IGS | *psbZ-trnG-GGC* |
| 22 | p1 | (A)10 | 10 | 36257 | 36266 | LSC | IGS | *trnG-GGC-trnfM-AUG* |
| 23 | p1 | (T)13 | 13 | 41647 | 41659 | LSC | IGS | *psaA-ycf3* |
| 24 | p1 | (A)20 | 20 | 42121 | 42140 | LSC | IGS | *psaA-ycf3* |
| 25 | p1 | (A)14 | 14 | 47695 | 47708 | LSC | IGS | *trnF-UUC-ndhJ* |
| 26 | p1 | (T)14 | 14 | 53930 | 53943 | LSC | IGS | *atpB-rbcL* |
| 27 | p1 | (A)12 | 12 | 55993 | 56004 | LSC | IGS | *rbcL-accD* |
| 28 | p1 | (T)13 | 13 | 56282 | 56294 | LSC | IGS | *rbcL-accD* |
| 29 | p1 | (T)10 | 10 | 56869 | 56878 | LSC | CDS | *accD* |
| 30 | p2 | (AT)5 | 10 | 59202 | 59211 | LSC | IGS/CDS | *psaI-ycf4/ycf4* |
| 31 | c | (TA)5(AT)6(TAAA)3 | 138 | 62639 | 62776 | LSC | IGS | *petA-psbJ* |
| 32 | p1 | (T)13 | 13 | 64096 | 64108 | LSC | IGS | *psbE-petL* |
| 33 | p3 | (AAT)5 | 15 | 64469 | 64483 | LSC | IGS | *psbE-petL* |
| 34 | p4 | (AAAT)3 | 12 | 64705 | 64716 | LSC | IGS | *psbE-petL* |
| 35 | p1 | (A)11 | 11 | 64883 | 64893 | LSC | IGS | *psbE-petL* |
| 36 | p1 | (T)12 | 12 | 66251 | 66262 | LSC | IGS | *trnP-CCA-psaJ* |
| 37 | c | (AT)7(T)11 | 122 | 67345 | 67466 | LSC | IGS | *rpl33-rps18* |
| 38 | p1 | (A)13 | 13 | 67934 | 67946 | LSC | IGS | *rps18-rpl20* |
| 39 | p1 | (T)17 | 17 | 71054 | 71070 | LSC | Intron | *clpP* |
| 40 | p1 | (A)14 | 14 | 76480 | 76493 | LSC | Intron | *petD* |
| 41 | p1 | (T)10 | 10 | 77601 | 77610 | LSC | IGS | *petD-rpoA* |
| 42 | p1 | (T)13 | 13 | 77810 | 77822 | LSC | CDS | *rpoA* |
| 43 | p2 | (TA)6 | 12 | 80275 | 80286 | LSC | IGS | *rps8-rpl14* |
| 44 | c | (T)11(A)11 | 48 | 81671 | 81718 | LSC | Intron | *rpl16* |
| 45 | p1 | (T)13 | 13 | 82399 | 82411 | LSC | Intron | *rpl16* |
| 46 | p2 | (GA)5 | 10 | 89565 | 89574 | IRB | CDS | *ycf2* |
| 47 | p6 | (GGAGCT)3 | 18 | 91412 | 91429 | IRB | CDS | *ycf2* |
| 48 | p2 | (TA)5 | 10 | 94338 | 94347 | IRB | IGS | *trnL-UUG-ndhB* |
| 49 | p1 | (A)10 | 10 | 99167 | 99176 | IRB | IGS | *rps12-trnV-GUC* |
| 50 | p1 | (A)11 | 11 | 108059 | 108069 | IRB | IGS | *rrn5S-trnR-CGU* |
| 51 | p1 | (T)11 | 11 | 108510 | 108520 | IRB | IGS | *trnR-CGU-trnN-AAC* |
| 52 | p1 | (A)10 | 10 | 110386 | 110395 | IRB | CDS | *ycf1* |
| 53 | p4 | (ATAG)3 | 12 | 112431 | 112442 | SSC | CDS | *ndhF* |
| 54 | p1 | (T)11 | 11 | 112681 | 112691 | SSC | IGS | *ndhF-rpl32* |
| 55 | p1 | (T)11 | 11 | 113796 | 113806 | SSC | IGS | *rpl32-trnL-CUA* |
| 56 | p4 | (AATT)3 | 12 | 113908 | 113919 | SSC | IGS | *rpl32-trnL-CUA* |
| 57 | p1 | (A)14 | 14 | 114906 | 114919 | SSC | CDS | *ccsA* |
| 58 | p1 | (A)10 | 10 | 117443 | 117452 | SSC | IGS | *ndhD-psaC* |
| 59 | p1 | (T)10 | 10 | 117971 | 117980 | SSC | IGS | *psaC-ndhE* |
| 60 | p2 | (AT)5 | 10 | 118415 | 118424 | SSC | IGS | *ndhE-ndhG* |
| 61 | p1 | (T)12 | 12 | 119138 | 119149 | SSC | IGS | *ndhG-ndhI* |
| 62 | p5 | (AATAC)3 | 15 | 119340 | 119354 | SSC | IGS | *ndhG-ndhI* |
| 63 | p1 | (A)10 | 10 | 121039 | 121048 | SSC | Intron | *ndhA* |
| 64 | p2 | (AT)5 | 10 | 121556 | 121565 | SSC | Intron | *ndhA* |
| 65 | c | (T)12(T)10(T)10 | 152 | 124322 | 124473 | SSC | CDS | *ycf1* |
| 66 | c | (T)10(T)11 | 64 | 125946 | 126009 | SSC | CDS | *ycf1* |
| 67 | p1 | (A)10 | 10 | 126160 | 126169 | SSC | CDS | *ycf1* |
| 68 | c | (TTC)4(T)11 | 67 | 127156 | 127222 | SSC | CDS | *ycf1* |
| 69 | p1 | (T)10 | 10 | 128377 | 128386 | IRA | CDS | *ycf1* |
| 70 | p1 | (A)11 | 11 | 130252 | 130262 | IRA | IGS | *trnN-AAC-trnR-CGU* |
| 71 | p1 | (T)11 | 11 | 130703 | 130713 | IRA | IGS | *trnR-CGU-rrn5S* |
| 72 | p1 | (T)10 | 10 | 139596 | 139605 | IRA | IGS | *trnV-GUC-rps12* |
| 73 | p2 | (AT)5 | 10 | 144424 | 144433 | IRA | IGS | *ndhB-trnL-UUG* |
| 74 | p6 | (AGCTCC)3 | 18 | 147343 | 147360 | IRA | CDS | *ycf2* |
| 75 | p2 | (TC)5 | 10 | 149198 | 149207 | IRA | CDS | *ycf2* |

**Supplementary table 6**: Types and number of simple sequence repeats motifs in *F. hamiltonii* and *F. occidentalis.*

| **Repeats** | **3** | **4** | **5** | **6** | **7** | **8** | **9** | **10** | **11** | **12** | **13** | **14** | **15** | **16** | **17** | **18** | **19** | **20** | **Total** |
| --- | --- | --- | --- | --- | --- | --- | --- | --- | --- | --- | --- | --- | --- | --- | --- | --- | --- | --- | --- |
| ***F. hamiltonii*** | | | | | | | | | | | | | | | | | | | |
| A | - | - | - | - | - | - | - | 10 | 6 | 3 | 3 | 3 |  | 1 |  | 1 |  |  | 27 |
| C | - | - | - | - | - | - | - | 1 |  |  |  |  |  |  |  |  |  |  | 1 |
| T | - | - | - | - | - | - | - | 14 | 6 | 3 | 4 | 3 | 1 | 1 |  |  |  |  | 32 |
| AT | - | - | 8 | 2 | 1 |  |  |  |  |  |  |  |  |  |  |  |  |  | 11 |
| TA | - | - | 8 | 2 |  |  |  |  |  |  |  |  |  |  |  |  |  |  | 10 |
| AAT | - | 2 | 1 |  |  |  |  |  |  |  |  |  |  |  |  |  |  |  | 3 |
| ATA | - | 1 |  |  |  |  |  |  |  |  |  |  |  |  |  |  |  |  | 1 |
| TTA | - | 1 |  |  |  |  |  |  |  |  |  |  |  |  |  |  |  |  | 1 |
| TTC | - | 1 |  |  |  |  |  |  |  |  |  |  |  |  |  |  |  |  | 1 |
| AAAT | 2 |  |  |  |  |  |  |  |  |  |  |  |  |  |  |  |  |  | 2 |
| AATT | 1 |  |  |  |  |  |  |  |  |  |  |  |  |  |  |  |  |  | 1 |
| ATAG | 1 |  |  |  |  |  |  |  |  |  |  |  |  |  |  |  |  |  | 1 |
| ATTT | 1 |  |  |  |  |  |  |  |  |  |  |  |  |  |  |  |  |  | 1 |
| CAAA | 1 |  |  |  |  |  |  |  |  |  |  |  |  |  |  |  |  |  | 1 |
| TAAA | 2 |  |  |  |  |  |  |  |  |  |  |  |  |  |  |  |  |  | 2 |
| TTTA | 1 |  |  |  |  |  |  |  |  |  |  |  |  |  |  |  |  |  | 1 |
| CTTTT | 1 |  |  |  |  |  |  |  |  |  |  |  |  |  |  |  |  |  | 1 |
| AGCTCC | 1 |  |  |  |  |  |  |  |  |  |  |  |  |  |  |  |  |  | 1 |
| GGAGCT | 1 |  |  |  |  |  |  |  |  |  |  |  |  |  |  |  |  |  | 1 |
|  |  |  |  |  |  |  |  |  |  |  |  |  |  |  |  |  |  |  |  |
| ***F. occidentalis*** | | | | | | | | | | | | | | | | | | | |
| A | - | - | - | - | - | - | - | 7 | 4 | 1 | 1 | 4 |  |  |  |  |  | 1 | 18 |
| T | - | - | - | - | - | - | - | 13 | 11 | 3 | 6 | 1 |  |  | 1 |  |  |  | 35 |
| AT | - | - | 10 | 2 | 1 |  |  |  |  |  |  |  |  |  |  |  |  |  | 13 |
| GA | - | - | 1 |  |  |  |  |  |  |  |  |  |  |  |  |  |  |  | 1 |
| TA | - | - | 4 | 2 |  |  |  |  |  |  |  |  |  |  |  |  |  |  | 6 |
| TC | - | - | 1 |  |  |  |  |  |  |  |  |  |  |  |  |  |  |  | 1 |
| AAT | - | 1 | 1 |  |  |  |  |  |  |  |  |  |  |  |  |  |  |  | 2 |
| TTA | - | 1 |  |  |  |  |  |  |  |  |  |  |  |  |  |  |  |  | 1 |
| TTC | - | 1 |  |  |  |  |  |  |  |  |  |  |  |  |  |  |  |  | 1 |
| AAAT | 1 |  |  |  |  |  |  |  |  |  |  |  |  |  |  |  |  |  | 1 |
| AATT | 1 |  |  |  |  |  |  |  |  |  |  |  |  |  |  |  |  |  | 1 |
| ATAG | 1 |  |  |  |  |  |  |  |  |  |  |  |  |  |  |  |  |  | 1 |
| CAAA | 1 |  |  |  |  |  |  |  |  |  |  |  |  |  |  |  |  |  | 1 |
| TAAA | 1 |  |  |  |  |  |  |  |  |  |  |  |  |  |  |  |  |  | 1 |
| TTTA | 1 |  |  |  |  |  |  |  |  |  |  |  |  |  |  |  |  |  | 1 |
| AATAC | 1 |  |  |  |  |  |  |  |  |  |  |  |  |  |  |  |  |  | 1 |
| AGCTCC | 1 |  |  |  |  |  |  |  |  |  |  |  |  |  |  |  |  |  | 1 |
| GGAGCT | 1 |  |  |  |  |  |  |  |  |  |  |  |  |  |  |  |  |  | 1 |

**Supplementary table 7:** Frequency of complementary simple sequence repeats in *F. hamiltonii* and *F. occidentalis.*

| **Repeats** | **3** | **4** | **5** | **6** | **7** | **8** | **9** | **10** | **11** | **12** | **13** | **14** | **15** | **16** | **17** | **18** | **19** | **20** | **Total** |
| --- | --- | --- | --- | --- | --- | --- | --- | --- | --- | --- | --- | --- | --- | --- | --- | --- | --- | --- | --- |
| ***Farsetia hamiltonii*** | | | | | | | | | | | | | | | | | | | |
| A/T | - | - | - | - | - | - | - | 24 | 12 | 6 | 7 | 6 | 1 | 2 |  | 1 |  |  | 59 |
| C/G | - | - | - | - | - | - | - | 1 |  |  |  |  |  |  |  |  |  |  | 1 |
| AT/AT | - | - | 16 | 4 | 1 |  |  |  |  |  |  |  |  |  |  |  |  |  | 21 |
| AAG/CTT | - | 1 |  |  |  |  |  |  |  |  |  |  |  |  |  |  |  |  | 1 |
| AAT/ATT | - | 4 | 1 |  |  |  |  |  |  |  |  |  |  |  |  |  |  |  | 5 |
| AAAC/GTTT | 1 |  |  |  |  |  |  |  |  |  |  |  |  |  |  |  |  |  | 1 |
| AAAT/ATTT | 6 |  |  |  |  |  |  |  |  |  |  |  |  |  |  |  |  |  | 6 |
| AATT/AATT | 1 |  |  |  |  |  |  |  |  |  |  |  |  |  |  |  |  |  | 1 |
| AGAT/ATCT | 1 |  |  |  |  |  |  |  |  |  |  |  |  |  |  |  |  |  | 1 |
| AAAAG/CTTTT | 1 |  |  |  |  |  |  |  |  |  |  |  |  |  |  |  |  |  | 1 |
| AGCTCC/AGCTGG | 2 |  |  |  |  |  |  |  |  |  |  |  |  |  |  |  |  |  | 2 |
|  |  |  |  |  |  |  |  |  |  |  |  |  |  |  |  |  |  |  |  |
| ***Farsetia occidentalis*** | | | | | | | | | | | | | | | | | | | |
|  |  |  |  |  |  |  |  |  |  |  |  |  |  |  |  |  |  |  |  |
| A/T | - | - | - | - | - | - | - | 20 | 15 | 4 | 7 | 5 |  |  | 1 |  |  | 1 | 53 |
| AG/CT | - | - | 2 |  |  |  |  |  |  |  |  |  |  |  |  |  |  |  | 2 |
| AT/AT | - | - | 14 | 4 | 1 |  |  |  |  |  |  |  |  |  |  |  |  |  | 19 |
| AAG/CTT | - | 1 |  |  |  |  |  |  |  |  |  |  |  |  |  |  |  |  | 1 |
| AAT/ATT | - | 2 | 1 |  |  |  |  |  |  |  |  |  |  |  |  |  |  |  | 3 |
| AAAC/GTTT | 1 |  |  |  |  |  |  |  |  |  |  |  |  |  |  |  |  |  | 1 |
| AAAT/ATTT | 3 |  |  |  |  |  |  |  |  |  |  |  |  |  |  |  |  |  | 3 |
| AATT/AATT | 1 |  |  |  |  |  |  |  |  |  |  |  |  |  |  |  |  |  | 1 |
| AGAT/ATCT | 1 |  |  |  |  |  |  |  |  |  |  |  |  |  |  |  |  |  | 1 |
| AATAC/ATTGT | 1 |  |  |  |  |  |  |  |  |  |  |  |  |  |  |  |  |  | 1 |
| AGCTCC/AGCTGG | 2 |  |  |  |  |  |  |  |  |  |  |  |  |  |  |  |  |  | 2 |

**Supplementary table 8**: Oligo-repeats analysis in *F. hamiltonii* and *F. occidentalis.*

| **Size** | **Position 1** | **Type** | **Position 2** | **Location** | **Region** | **IGS/CDS/Intron** |
| --- | --- | --- | --- | --- | --- | --- |
|  |  |  |  |  |  |  |
| ***F. hamiltonii*** | | | | | | |
| 28 | 125462 | P | 125462 | *ycf1* | SSC | CDS |
| 45 | 113810 | P | 113810 | *rpl32-trnL-CUA* | SSC | IGS |
| 23 | 113338 | R | 113338 | *ndhF-rpl32* | SSC | IGS |
| 29 | 113246 | P | 113246 | *ndhF-rpl32* | SSC | IGS |
| 22 | 109937 | P | 109937 | *ycf1* | IR | CDS |
| 29 | 107650 | F | 107682 | *rrn4.5S-rrn5S* | IR | IGS |
| 33 | 107646 | F | 107678 | *rrn4.5S-rrn5S* | IR | IGS |
| 28 | 91283 | F | 91301 | *ycf2* | IR | CDS |
| 29 | 88854 | F | 88876 | *ycf2* | IR | CDS |
| 38 | 88820 | F | 88844 | *ycf2* | IR | CDS |
| 41 | 88808 | F | 88832 | *ycf2* | IR | CDS |
| 21 | 83661 | R | 83661 | *rpl22* | LSC | CDS |
| 28 | 80150 | F | 80168 | *rps8-rpl14* | LSC | IGS |
| 31 | 77512 | R | 77512 | *petD-rpoA* | LSC | IGS |
| 28 | 77508 | R | 77519 | *petD-rpoA* | LSC | IGS |
| 44 | 73881 | P | 73881 | *psbT-psbN* | LSC | IGS |
| 28 | 71724 | R | 71735 | *clpP-psbB* | LSC | IGS |
| 21 | 71711 | R | 71711 | *clpP-psbB* | LSC | IGS |
| 27 | 71196 | P | 71197 | *clpP* | LSC | Intron |
| 34 | 71193 | P | 71193 | *clpP* | LSC | Intron |
| 22 | 71139 | F | 71161 | *clpP* | LSC | Intron |
| 28 | 69713 | P | 99913 | *clpP/rps12-trnV-GUC* | LSC/IR | Intron/IGS |
| 28 | 64900 | R | 64900 | *psbE-petL* | LSC | IGS |
| 29 | 62560 | P | 62565 | *petA-psbJ* | LSC | IGS |
| 31 | 62508 | F | 62550 | *petA-psbJ* | LSC | IGS |
| 52 | 62487 | F | 62528 | *petA-psbJ* | LSC | IGS |
| 23 | 58743 | F | 58753 | *psaI-ycf4* | LSC | IGS |
| 21 | 55740 | P | 55768 | *rbcL-accD* | LSC | IGS |
| 39 | 51469 | P | 51469 | *trnM-AUG-atpE* | LSC | IGS |
| 32 | 49974 | P | 80175 | *ndhC-trnV-GUA/rpl16* | LSC | IGS/CDS |
| 28 | 46089 | P | 46089 | *trnT-ACA-trnL-UUA* | LSC | IGS |
| 29 | 44119 | P | 44124 | *ycf3-trnS-UCC* | LSC | IGS |
| 27 | 43287 | F | 98598 | *ycf3/rps12-trnV-GUC* | LSC/IR | Intron/IGS |
| 20 | 42967 | R | 64027 | *ycf3/psbE-petL* | LSC | Intron/IGS |
| 21 | 41527 | P | 114898 | *psaA-ycf3/ccsA* | LSC/SSC | IGS/CDS |
| 38 | 38173 | F | 40398 | *psaB/psaA* | LSC | CDS |
| 52 | 38170 | F | 40394 | *psaB/psaA* | LSC | CDS |
| 56 | 38149 | F | 40373 | *psaB/psaA* | LSC | CDS |
| 29 | 35766 | C | 113333 | *psbZ-trnG-GGC/ndhF-rpl32* | LSC/SSC | IGS |
| 35 | 35756 | P | 35756 | *psbZ-trnG-GGC* | LSC | IGS |
| 27 | 35750 | P | 35769 | *psbZ-trnG-GGC* | LSC | IGS |
| 28 | 34875 | P | 44315 | *trnS-UCA/ycf3-trnS-UCC* | LSC | trn/IGS |
| 25 | 34809 | P | 44378 | *trnS-UCA/trnS-UCC* | LSC | trn |
| 30 | 32121 | F | 32138 | *trnT-ACC-psbD* | LSC | IGS |
| 32 | 32119 | F | 32132 | *trnT-ACC-psbD* | LSC | IGS |
| 20 | 31852 | P | 82306 | *trnT-ACC-psbD/rpl22* | LSC | IGS/CDS |
| 20 | 31849 | F | 76410 | *trnT-ACC-psbD/petD* | LSC | IGS/Intron |
| 27 | 31503 | F | 114115 | *trnT-ACC-psbD/rpl32-trnL-CUA* | LSC/SSC | IGS |
| 20 | 30949 | F | 30960 | *trnE-GAA-trnT-ACC* | LSC | IGS |
| 21 | 30921 | R | 30921 | *trnE-GAA-trnT-ACC* | LSC | IGS |
| 24 | 30659 | P | 30659 | *trnE-GAA-trnT-ACC* | LSC | IGS |
| 40 | 28625 | P | 28625 | *petN-psbM* | LSC | IGS |
| 22 | 27159 | P | 27159 | *rpoB-trnC-UGC* | LSC | IGS |
| 20 | 22417 | R | 22417 | *rpoC1* | LSC | Intron |
| 38 | 22070 | F | 22103 | *rpoC1* | LSC | Intron |
| 20 | 13651 | P | 13651 | *atpH-atpI* | LSC | IGS |
| 20 | 13543 | F | 87817 | *atpH-atpI/ycf2* | LSC/IR | IGS/CDS |
| 27 | 9730 | F | 50686 | *trnR-AGA-atpA/trnV-GUA* | LSC | IGS/Intron |
| 31 | 9378 | F | 35974 | *trnG-GGA/trnG-GGC* | LSC | trn |
| 21 | 7853 | F | 34813 | *trnS-AGC/trnS-UCA* | LSC | trn |
| 25 | 7849 | F | 34809 | *trnS-AGC/trnS-UCA* | LSC | trn |
| 28 | 7849 | P | 44375 | *trnS-AGC/trnS-UCC* | LSC | trn |
| 29 | 7845 | F | 34806 | *psbI-trnS-AGC/psbC-trnS-UCA* | LSC | IGS |
| 28 | 7521 | P | 7523 | *psbK-psbI* | LSC | IGS |
| 20 | 7517 | C | 31853 | *psbK-psbI/trnT-ACC-psbD* | LSC | IGS |
| 25 | 6355 | C | 35773 | *rps16-trnQ-CAA/psbZ-trnG-GGC* | LSC | IGS |
| 29 | 6351 | C | 35769 | *rps16-trnQ-CAA/psbZ-trnG-GGC* | LSC | IGS |
| 22 | 6350 | P | 6350 | *rps16-trnQ-CAA* | LSC | IGS |
| 29 | 6345 | P | 31035 | *rps16-trnQ-CAA/trnE-GAA-trnT-ACC* | LSC | IGS |
| 29 | 5827 | F | 118324 | *rps16/ndhE-ndhG* | LSC/SSC | Intron/IGS |
| 32 | 5824 | F | 118320 | *rps16/ndhE-ndhG* | LSC/SSC | Intron/IGS |
| 20 | 4370 | C | 124429 | *trnK-AAA-rps16/ycf1* | LSC/SSC | IGS/CDS |
| 27 | 258 | P | 292 | *trnH-CAC-psbA* | LSC | IGS |
| ***F. occidentalis*** | | | | | | |
| 28 | 125480 | P | 125480 | *ycf1* | SSC | CDS |
| 35 | 113850 | P | 113850 | *rpl32-trnL-CUA* | SSC | IGS |
| 32 | 113377 | R | 113384 | *ndhF-rpl32* | SSC | IGS |
| 29 | 113292 | P | 113292 | *ndhF-rpl32* | SSC | IGS |
| 28 | 113089 | R | 113089 | *ndhF-rpl32* | SSC | IGS |
| 22 | 109982 | P | 109982 | *ycf1* | IR | CDS |
| 29 | 107700 | F | 107732 | *rrn4.4S-rrn5S* | IR | IGS |
| 33 | 107696 | F | 107728 | *rrn4.4S-rrn5S* | IR | IGS |
| 28 | 91351 | F | 91369 | *ycf2* | IR | CDS |
| 29 | 88922 | F | 88944 | *ycf2* | IR | CDS |
| 38 | 88888 | F | 88912 | *ycf2* | IR | CDS |
| 38 | 88883 | F | 88907 | *ycf2* | IR | CDS |
| 41 | 88876 | F | 88900 | *ycf2* | IR | CDS |
| 21 | 83735 | R | 83735 | *rpl22* | LSC | CDS |
| 32 | 77589 | R | 77589 | *petD-rpoA* | LSC | IGS |
| 44 | 73957 | P | 73957 | *psbT-psbN* | LSC | IGS |
| 28 | 71799 | R | 71810 | *clpP-psbB* | LSC | IGS |
| 21 | 71786 | R | 71786 | *clpP-psbB* | LSC | IGS |
| 27 | 71271 | P | 71272 | *clpP* | LSC | Intron |
| 34 | 71268 | P | 71268 | *clpP* | LSC | Intron |
| 22 | 71214 | F | 71236 | *clpP* | LSC | Intron |
| 27 | 69794 | P | 99963 | *clpP/rps12-trnV-GUC* | LSC/IR | Intron/IGS |
| 21 | 65186 | R | 65186 | *psbE-petL* | LSC | IGS |
| 28 | 64974 | R | 64974 | *psbE-petL* | LSC | IGS |
| 34 | 62638 | P | 62648 | *petA-psbJ* | LSC | IGS |
| 28 | 62638 | R | 62638 | *petA-psbJ* | LSC | IGS |
| 23 | 58874 | F | 58884 | *psaI-ycf4* | LSC | IGS |
| 21 | 55870 | P | 55898 | *rbcL-accD* | LSC | IGS |
| 39 | 51602 | P | 51602 | *trnM-AUG-atpE* | LSC | IGS |
| 22 | 50111 | R | 50111 | *ndhC-trnV-GUA* | LSC | IGS |
| 31 | 50099 | R | 50099 | *ndhC-trnV-GUA* | LSC | IGS |
| 32 | 46203 | F | 46230 | *trnT-ACA-trnL-UUA* | LSC | IGS |
| 29 | 44234 | P | 44239 | *ycf3-trnS-UCC* | LSC | IGS |
| 27 | 43402 | F | 98648 | *ycf3-trnS-UCC/rps12-trnV-GUC* | LSC/IR | IGS |
| 25 | 42827 | P | 42827 | *ycf3* | LSC | Intron |
| 46 | 38289 | F | 40513 | *psaB/psaA* | LSC | CDS |
| 56 | 38268 | F | 40492 | *psaB/psaA* | LSC | CDS |
| 29 | 35891 | C | 113379 | *psbZ-trnG-GGC/ndhF-rpl32* | LSC/SSC | IGS |
| 35 | 35881 | P | 35881 | *psbZ-trnG-GGC* | LSC | IGS |
| 27 | 35875 | P | 35894 | *psbZ-trnG-GGC* | LSC | IGS |
| 28 | 35008 | P | 44430 | *trnS-UCA/ycf3-trnS-UCC* | LSC | trn/IGS |
| 25 | 34942 | P | 44493 | *trnS-UCA/trnS-UCC* | LSC | trn |
| 32 | 32242 | F | 32255 | *trnT-ACC-psbD* | LSC | IGS |
| 26 | 31923 | F | 31949 | *trnT-ACC-psbD* | LSC | IGS |
| 27 | 31608 | F | 114136 | *trnT-ACC-psbD/rpl32-trnL-CUA* | LSC/SSC | IGS |
| 25 | 31140 | F | 80270 | *trnE-GAA-trnT-ACC/rps8-rpl14* | LSC | IGS |
| 20 | 31101 | F | 31121 | *trnE-GAA-trnT-ACC* | LSC | IGS |
| 20 | 31030 | F | 31041 | *trnE-GAA-trnT-ACC* | LSC | IGS |
| 21 | 31003 | R | 31003 | *trnE-GAA-trnT-ACC* | LSC | IGS |
| 22 | 30810 | R | 30810 | *trnE-GAA-trnT-ACC* | LSC | IGS |
| 24 | 30731 | P | 30731 | *trnE-GAA-trnT-ACC* | LSC | IGS |
| 40 | 28701 | P | 28701 | *petN-psbM* | LSC | IGS |
| 22 | 27170 | P | 27170 | *rpoB-trnC-UGC* | LSC | IGS |
| 22 | 26931 | F | 26951 | *rpoB-trnC-UGC* | LSC | IGS |
| 20 | 22399 | R | 22399 | *rpoC1* | LSC | Intron |
| 20 | 13631 | P | 13631 | *atpH-atpI* | LSC | IGS |
| 20 | 13523 | F | 87891 | *atpH-atpI/ycf2* | LSC/IR | IGS/CDS |
| 31 | 9366 | F | 36099 | *trnG-GGA/trnG-GGC* | LSC | trn |
| 20 | 8056 | R | 28972 | *trnS-AGC-trnG-GGA/psbM-trnD-GAC* | LSC | IGS |
| 28 | 7827 | P | 44490 | *trnS-AGC/trnS-UCC* | LSC | trn |
| 29 | 7823 | F | 34939 | *psbI-trnS-AGC/psbC-trnS-UCA* | LSC | IGS |
| 26 | 7738 | P | 112611 | *psbI/ndhF-rpl32* | LSC/SSC | CDS/IGS |
| 26 | 6350 | F | 31144 | *rps16-trnQ-CAA/trnE-GAA-trnT-ACC* | LSC | IGS |
| 34 | 6342 | F | 31136 | *rps16-trnQ-CAA/trnE-GAA-trnT-ACC* | LSC | IGS |
| 29 | 6341 | C | 35898 | *rps16-trnQ-CAA/psbZ-trnG-GGC* | LSC | IGS |
| 28 | 6336 | C | 35882 | *rps16-trnQ-CAA/psbZ-trnG-GGC* | LSC | IGS |
| 22 | 6336 | P | 6336 | *rps16-trnQ-CAA* | LSC | IGS |
| 28 | 6336 | R | 35888 | *rps16-trnQ-CAA/psbZ-trnG-GGC* | LSC | IGS |
| 30 | 4658 | F | 4687 | *trnK-AAA-rps16* | LSC | IGS |
| 20 | 4345 | F | 76479 | *trnK-AAA-rps16/petD* | LSC | IGS/Intron |
| 21 | 3807 | R | 3807 | *trnK-AAA* | LSC | Intron |
| 25 | 3797 | R | 3797 | *trnK-AAA* | LSC | Intron |
| 27 | 263 | P | 297 | *trnH-CAC-psbA* | LSC | IGS |

**Supplementary table 9:** *Farsetia hamiltonii,* and 21 selected NCBI genomes of Brassicaceae family for Phylogenetic tree analysis. Two species of *Calotropis* (*Calotropis procera,Calotropis gigantea*) of family Apocynaceae were used as outgroup.

|  | **Family** | **Clade** | **Tribe Name** | **Species** | **Gene bank accession number** |
| --- | --- | --- | --- | --- | --- |
|  | **Brassicaceae** | **Clade A** | **Tribe Microlepidieae** | *Stenopetalum lineare* | MK637800 |
|  |  |  | **Tribe Cardamineae** | *Nasturtium officinale* | MK045962 |
|  |  |  | **Tribe Camelineae** | *Arabidopsis thaliana* | MK380721 |
|  |  | **Clade B** | **Tribe Isatideae** | *Isatis tinctoria* | KT591187 |
|  |  |  | **Tribe Brassiceae** | *Brassica rapa* | MT726210 |
|  |  |  | **Tribe Sisymbrieae** | *Sisymbrium altissimum* | MK637790 |
|  |  |  | **Tribe Coluteocarpeae** | *Noccaea caerulescens* | MK637763 |
|  |  | **Clade C** | **Tribe Anastaticeae** | ***Farsetia hamiltonii*** | MT884003 |
|  |  |  |  | *Farsetia occidentalis* | MK637823 |
|  |  |  |  | *Lobularia libyca* | KY912029 |
|  |  |  |  | *Notoceras bicorne* | MK637762 |
|  |  |  |  | *Parolinia ornata* | MK637776 |
|  |  |  |  | *Morettia canescens* | KY912031 |
|  |  |  | **Tribe Cochlearieae** | *Cochlearia borzaeana* | LN866844 |
|  |  |  | **Tribe Megacarpaeeae** | *Megacarpaea polyandra* | MK637758 |
|  |  |  | **Tribe Biscutelleae** | *Biscutella laevigata* | MK637669 |
|  |  |  | **Tribe Iberideae** | *Iberis amara* | MK637733 |
|  |  | **Clade D** | **Tribe Alysseae** | *Aurinia corymbosa* | MK637650 |
|  |  | **Clade E** | **Tribe Euclidieae** | *Pycnoplinthus uniflorus* | MT845156 |
|  |  |  | **Tribe Chorisporeae** | *Chorispora tenella* | MK637697 |
|  |  |  | **Tribe Dontostemoneae** | *Clausia trichosepala* | MK637698 |
|  |  | **Clade F** | **Tribe Aethionemeae** | *Aethionema grandiflorum* | AP009367 |
| **Outgroup** | **Apocynaceae** |  | **Tribe Asclepiadeae** | *Calotropis procera* | MH939982 |
|  |  |  | **Tribe Asclepiadeae** | *Calotropis gigantea* | KJ953907 |

**Supplementary table 10:** Non-synonymous (Ka) and synonymous rate (Ks) of substitution, Ka/Ks Value in *F. hamiltonii* by making pairwise alignment with *Farsetia occidentalis* and eight other species of Clade C of Brassicaceae I.e. *Lobularia libyca, Notoceras bicorne, Parolinia ornata, Morettia canescens, Cochlearia borzaeana, Megacarpaea polyandra, Biscutella laevigata, and Iberis amara*.

| **GENES** | **Species which Pairwise aligned with *F. hamiltonii*** | **Ks** | **Ka** | **Ka/Ks** |
| --- | --- | --- | --- | --- |
| ***accD*** | *Farsetia occidentalis* | 0.0033 | 0.0035 | 1.060606061 |
|  | *Lobularia libyca* | 0.0374 | 0.0265 | 0.70855615 |
|  | *Notoceras bicorne* | 0.0269 | 0.0301 | 1.118959108 |
|  | *Parolinia ornata* | 0.0134 | 0.0358 | 2.671641791 |
|  | *Morettia canescens* | 0.0153 | 0.0394 | 2.575163399 |
|  | *Cochlearia borzaeana* | 0.0373 | 0.041 | 1.09919571 |
|  | *Megacarpaea polyandra* | 0.0168 | 0.0283 | 1.68452381 |
|  | *Biscutella laevigata* | 0.0392 | 0.0406 | 1.035714286 |
|  | *Iberis amara* | 0.0349 | 0.038 | 1.088825215 |
| ***atpA*** | *Farsetia occidentalis* | 0.0054 | 0 | 0 |
|  | *Lobularia libyca* | 0.0442 | 0.0026 | 0.058823529 |
|  | *Notoceras bicorne* | 0.0472 | 0.0017 | 0.036016949 |
|  | *Parolinia ornata* | 0.0329 | 0 | 0 |
|  | *Morettia canescens* | 0.0414 | 0 | 0 |
|  | *Cochlearia borzaeana* | 0.0911 | 0.0026 | 0.028540066 |
|  | *Megacarpaea polyandra* | 0.05 | 0.0009 | 0.018 |
|  | *Biscutella laevigata* | 0.091 | 0.0017 | 0.018681319 |
|  | *Iberis amara* | 0.0733 | 0.0017 | 0.02319236 |
| ***atpB*** | *Farsetia occidentalis* | 0.0054 | 0.0009 | 0.166666667 |
|  | *Lobularia libyca* | 0.0389 | 0.0071 | 0.18251928 |
|  | *Notoceras bicorne* | 0.0387 | 0.0027 | 0.069767442 |
|  | *Parolinia ornata* | 0.0247 | 0 | 0 |
|  | *Morettia canescens* | 0.0275 | 0 | 0 |
|  | *Cochlearia borzaeana* | 0.0594 | 0.0027 | 0.045454545 |
|  | *Megacarpaea polyandra* | 0.0275 | 0.0009 | 0.032727273 |
|  | *Biscutella laevigata* | 0.0445 | 0.0027 | 0.060674157 |
|  | *Iberis amara* | 0.0562 | 0 | 0 |
| ***atpE*** | *Farsetia occidentalis* | 0 | 0 | 0 |
|  | *Lobularia libyca* | 0.0219 | 0 | 0 |
|  | *Notoceras bicorne* | 0.0109 | 0 | 0 |
|  | *Parolinia ornata* | 0 | 0 | 0 |
|  | *Morettia canescens* | 0.0109 | 0 | 0 |
|  | *Cochlearia borzaeana* | 0.0273 | 0.0083 | 0.304029304 |
|  | *Megacarpaea polyandra* | 0.022 | 0.0066 | 0.3 |
|  | *Biscutella laevigata* | 0.0447 | 0 | 0 |
|  | *Iberis amara* | 0.0445 | 0.0033 | 0.074157303 |
| ***atpF*** | *Farsetia occidentalis* | 0 | 0 | 0 |
|  | *Lobularia libyca* | 0.0339 | 0.0117 | 0.345132743 |
|  | *Notoceras bicorne* | 0.0339 | 0.0047 | 0.138643068 |
|  | *Parolinia ornata* | 0.0254 | 0.0046 | 0.181102362 |
|  | *Morettia canescens* | 0.0253 | 0.0023 | 0.090909091 |
|  | *Cochlearia borzaeana* | 0.0425 | 0.0141 | 0.331764706 |
|  | *Megacarpaea polyandra* | 0.0603 | 0.0164 | 0.271973466 |
|  | *Biscutella laevigata* | 0.0826 | 0.0152 | 0.18401937 |
|  | *Iberis amara* | 0.0515 | 0.0164 | 0.318446602 |
| ***atpH*** | *Farsetia occidentalis* | 0.0153 | 0 | 0 |
|  | *Lobularia libyca* | 0.0309 | 0 | 0 |
|  | *Notoceras bicorne* | 0.0153 | 0 | 0 |
|  | *Parolinia ornata* | 0.0153 | 0 | 0 |
|  | *Morettia canescens* | 0.0309 | 0 | 0 |
|  | *Cochlearia borzaeana* | 0.0964 | 0.0057 | 0.059128631 |
|  | *Megacarpaea polyandra* | 0.063 | 0 | 0 |
|  | *Biscutella laevigata* | 0.0797 | 0 | 0 |
|  | *Iberis amara* | 0.1509 | 0 | 0 |
| ***atpI*** | *Farsetia occidentalis* | 0.0055 | 0 | 0 |
|  | *Lobularia libyca* | 0.0281 | 0.0035 | 0.12455516 |
|  | *Notoceras bicorne* | 0.0339 | 0 | 0 |
|  | *Parolinia ornata* | 0.0167 | 0 | 0 |
|  | *Morettia canescens* | 0.0339 | 0 | 0 |
|  | *Cochlearia borzaeana* | 0.0396 | 0.0053 | 0.133838384 |
|  | *Megacarpaea polyandra* | 0.0282 | 0.0035 | 0.124113475 |
|  | *Biscutella laevigata* | 0.0694 | 0 | 0 |
|  | *Iberis amara* | 0.0514 | 0.0018 | 0.035019455 |
| ***ccsA*** | *Farsetia occidentalis* | 0.0045 | 0.0013 | 0.288888889 |
|  | *Lobularia libyca* | 0.0329 | 0.0172 | 0.522796353 |
|  | *Notoceras bicorne* | 0.0525 | 0.0199 | 0.379047619 |
|  | *Parolinia ornata* | 0.0377 | 0.0052 | 0.137931034 |
|  | *Morettia canescens* | 0.0186 | 0.0079 | 0.424731183 |
|  | *Cochlearia borzaeana* | 0.05 | 0.0245 | 0.49 |
|  | *Megacarpaea polyandra* | 0.0354 | 0.0219 | 0.618644068 |
|  | *Biscutella laevigata* | 0.0377 | 0.0252 | 0.668435013 |
|  | *Iberis amara* | 0.0644 | 0.0383 | 0.594720497 |
| ***cemA*** | *Farsetia occidentalis* | 0.0069 | 0.0019 | 0.275362319 |
|  | *Lobularia libyca* | 0.0277 | 0.0093 | 0.335740072 |
|  | *Notoceras bicorne* | 0.0422 | 0.0019 | 0.045023697 |
|  | *Parolinia ornata* | 0.0138 | 0.0037 | 0.268115942 |
|  | *Morettia canescens* | 0 | 0.0037 | 0 |
|  | *Cochlearia borzaeana* | 0.0426 | 0.0168 | 0.394366197 |
|  | *Megacarpaea polyandra* | 0.0069 | 0.0037 | 0.536231884 |
|  | *Biscutella laevigata* | 0.0352 | 0.0112 | 0.318181818 |
|  | *Iberis amara* | 0.0351 | 0.0225 | 0.641025641 |
| ***clpP*** | *Farsetia occidentalis* | 0 | 0 | 0 |
|  | *Lobularia libyca* | 0.0371 | 0.0022 | 0.059299191 |
|  | *Notoceras bicorne* | 0.0146 | 0.0045 | 0.308219178 |
|  | *Parolinia ornata* | 0.0369 | 0 | 0 |
|  | *Morettia canescens* | 0.022 | 0 | 0 |
|  | *Cochlearia borzaeana* | 0.0295 | 0.0045 | 0.152542373 |
|  | *Megacarpaea polyandra* | 0.0146 | 0 | 0 |
|  | *Biscutella laevigata* | 0.0221 | 0.0089 | 0.402714932 |
|  | *Iberis amara* | 0.0145 | 0.0112 | 0.772413793 |
| ***matK*** | *Farsetia occidentalis* | 0.0085 | 0.0041 | 0.482352941 |
|  | *Lobularia libyca* | 0.0768 | 0.0138 | 0.1796875 |
|  | *Notoceras bicorne* | 0.0952 | 0.019 | 0.199579832 |
|  | *Parolinia ornata* | 0.0435 | 0.014 | 0.32183908 |
|  | *Morettia canescens* | 0.0464 | 0.0115 | 0.247844828 |
|  | *Cochlearia borzaeana* | 0.1107 | 0.0296 | 0.267389341 |
|  | *Megacarpaea polyandra* | 0.071 | 0.0149 | 0.209859155 |
|  | *Biscutella laevigata* | 0.0187 | 0.0506 | 2.705882353 |
|  | *Iberis amara* | 0.0912 | 0.0228 | 0.25 |
| ***ndhA*** | *Farsetia occidentalis* | 0.019 | 0.0012 | 0.063157895 |
|  | *Lobularia libyca* | 0.084 | 0.0049 | 0.058333333 |
|  | *Notoceras bicorne* | 0.0585 | 0.0025 | 0.042735043 |
|  | *Parolinia ornata* | 0.0384 | 0.0012 | 0.03125 |
|  | *Morettia canescens* | 0.0463 | 0.0037 | 0.079913607 |
|  | *Cochlearia borzaeana* | 0.0872 | 0.0074 | 0.084862385 |
|  | *Megacarpaea polyandra* | 0.0668 | 0.0111 | 0.166167665 |
|  | *Biscutella laevigata* | 0.0955 | 0.0049 | 0.051308901 |
|  | *Iberis amara* | 0.0849 | 0.0156 | 0.183745583 |
| ***ndhB*** | *Farsetia occidentalis* | 0 | 0 | 0 |
|  | *Lobularia libyca* | 0.0136 | 0 | 0 |
|  | *Notoceras bicorne* | 0 | 0 | 0 |
|  | *Parolinia ornata* | 0 | 0 | 0 |
|  | *Morettia canescens* | 0 | 0 | 0 |
|  | *Cochlearia borzaeana* | 0.0027 | 0.0034 | 1.259259259 |
|  | *Megacarpaea polyandra* | 0 | 0.0046 | 0 |
|  | *Biscutella laevigata* | 0.0034 | 0.0034 | 1 |
|  | *Iberis amara* | 0.0034 | 0.0046 | 1.352941176 |
| ***ndhC*** | *Farsetia occidentalis* | 0.0116 | 0 | 0 |
|  | *Lobularia libyca* | 0.0722 | 0 | 0 |
|  | *Notoceras bicorne* | 0.0473 | 0 | 0 |
|  | *Parolinia ornata* | 0.0233 | 0 | 0 |
|  | *Morettia canescens* | 0.0233 | 0 | 0 |
|  | *Cochlearia borzaeana* | 0.0474 | 0.0074 | 0.156118143 |
|  | *Megacarpaea polyandra* | 0.0474 | 0 | 0 |
|  | *Biscutella laevigata* | 0.0353 | 0 | 0 |
|  | *Iberis amara* | 0.0977 | 0.0037 | 0.037871034 |
| ***ndhD*** | *Farsetia occidentalis* | 0.0112 | 0.0026 | 0.232142857 |
|  | *Lobularia libyca* | 0.0485 | 0.007 | 0.144329897 |
|  | *Notoceras bicorne* | 0.0516 | 0.0052 | 0.100775194 |
|  | *Parolinia ornata* | 0.0167 | 0.007 | 0.419161677 |
|  | *Morettia canescens* | 0.0225 | 0.0035 | 0.155555556 |
|  | *Cochlearia borzaeana* | 0.0666 | 0.0113 | 0.16966967 |
|  | *Megacarpaea polyandra* | 0.0225 | 0.0061 | 0.271111111 |
|  | *Biscutella laevigata* | 0.0695 | 0.0096 | 0.138129496 |
|  | *Iberis amara* | 0.0756 | 0.0052 | 0.068783069 |
| ***ndhE*** | *Farsetia occidentalis* | 0 | 0 | 0 |
|  | *Lobularia libyca* | 0.0438 | 0.013 | 0.296803653 |
|  | *Notoceras bicorne* | 0.0747 | 0.0043 | 0.057563588 |
|  | *Parolinia ornata* | 0.044 | 0.0086 | 0.195454545 |
|  | *Morettia canescens* | 0.029 | 0.0043 | 0.148275862 |
|  | *Cochlearia borzaeana* | 0.0745 | 0.0218 | 0.29261745 |
|  | *Megacarpaea polyandra* | 0.0143 | 0.013 | 0.909090909 |
|  | *Biscutella laevigata* | 0.0288 | 0.013 | 0.451388889 |
|  | *Iberis amara* | 0.0441 | 0.0174 | 0.394557823 |
| ***ndhF*** | *Farsetia occidentalis* | 0.0059 | 0.0041 | 0.694915254 |
|  | *Lobularia libyca* | 0.0339 | 0.0105 | 0.309734513 |
|  | *Notoceras bicorne* | 0.0505 | 0.0117 | 0.231683168 |
|  | *Parolinia ornata* | 0.0178 | 0.0064 | 0.359550562 |
|  | *Morettia canescens* | 0.036 | 0.0058 | 0.161111111 |
|  | *Cochlearia borzaeana* | 0.0837 | 0.0208 | 0.248506571 |
|  | *Megacarpaea polyandra* | 0.0392 | 0.0114 | 0.290816327 |
|  | *Biscutella laevigata* | 0.074 | 0.0182 | 0.245945946 |
|  | *Iberis amara* | 0.0675 | 0.0217 | 0.321481481 |
| ***ndhG*** | *Farsetia occidentalis* | 0 | 0 | 0 |
|  | *Lobularia libyca* | 0.0389 | 0.0025 | 0.064267352 |
|  | *Notoceras bicorne* | 0.0232 | 0.0153 | 0.659482759 |
|  | *Parolinia ornata* | 0.0311 | 0 | 0 |
|  | *Morettia canescens* | 0.0232 | 0 | 0 |
|  | *Cochlearia borzaeana* | 0.0805 | 0.0204 | 0.253416149 |
|  | *Megacarpaea polyandra* | 0.0233 | 0.0051 | 0.21888412 |
|  | *Biscutella laevigata* | 0.0473 | 0.0127 | 0.268498943 |
|  | *Iberis amara* | 0.0719 | 0.0076 | 0.105702364 |
| ***ndhH*** | *Farsetia occidentalis* | 0.0038 | 0 | 0 |
|  | *Lobularia libyca* | 0.0504 | 0.0011 | 0.021825397 |
|  | *Notoceras bicorne* | 0.0385 | 0.0055 | 0.142857143 |
|  | *Parolinia ornata* | 0.0386 | 0.0011 | 0.028497409 |
|  | *Morettia canescens* | 0 | 0 | 0 |
|  | *Cochlearia borzaeana* | 0.0791 | 0.0033 | 0.041719343 |
|  | *Megacarpaea polyandra* | 0.0466 | 0.0022 | 0.0472103 |
|  | *Biscutella laevigata* | 0.0626 | 0.0055 | 0.087859425 |
|  | *Iberis amara* | 0.0587 | 0.0066 | 0.112436116 |
| ***ndhI*** | *Farsetia occidentalis* | 0.0087 | 0 | 0 |
|  | *Lobularia libyca* | 0.0722 | 0 | 0 |
|  | *Notoceras bicorne* | 0.0535 | 0 | 0 |
|  | *Parolinia ornata* | 0.0535 | 0 | 0 |
|  | *Morettia canescens* | 0.0445 | 0.0026 | 0.058426966 |
|  | *Cochlearia borzaeana* | 0.046 | 0.0054 | 0.117391304 |
|  | *Megacarpaea polyandra* | 0.0353 | 0.0026 | 0.073654391 |
|  | *Biscutella laevigata* | 0.0817 | 0 | 0 |
|  | *Iberis amara* | 0.1015 | 0.0078 | 0.076847291 |
| ***ndhJ*** | *Farsetia occidentalis* | 0 | 0 | 0 |
|  | *Lobularia libyca* | 0.057 | 0 | 0 |
|  | *Notoceras bicorne* | 0.0185 | 0 | 0 |
|  | *Parolinia ornata* | 0.0092 | 0 | 0 |
|  | *Morettia canescens* | 0.0185 | 0 | 0 |
|  | *Cochlearia borzaeana* | 0.0374 | 0.0055 | 0.147058824 |
|  | *Megacarpaea polyandra* | 0.0185 | 0 | 0 |
|  | *Biscutella laevigata* | 0.0469 | 0 | 0 |
|  | *Iberis amara* | 0.0185 | 0.0027 | 0.145945946 |
| ***petA*** | *Farsetia occidentalis* | 0.0044 | 0 | 0 |
|  | *Lobularia libyca* | 0.06 | 0.0027 | 0.045 |
|  | *Notoceras bicorne* | 0.0551 | 0.0027 | 0.049001815 |
|  | *Parolinia ornata* | 0.0271 | 0.0027 | 0.099630996 |
|  | *Morettia canescens* | 0.0225 | 0.0027 | 0.12 |
|  | *Cochlearia borzaeana* | 0.0504 | 0.0082 | 0.162698413 |
|  | *Megacarpaea polyandra* | 0.0318 | 0.0082 | 0.257861635 |
|  | *Biscutella laevigata* | 0.041 | 0.0096 | 0.234146341 |
|  | *Iberis amara* | 0.0791 | 0.0082 | 0.103666245 |
| ***petB*** | *Farsetia occidentalis* | 0 | 0 | 0 |
|  | *Lobularia libyca* | 0.0127 | 0 | 0 |
|  | *Notoceras bicorne* | 0.0388 | 0 | 0 |
|  | *Parolinia ornata* | 0.0192 | 0 | 0 |
|  | *Morettia canescens* | 0.0128 | 0 | 0 |
|  | *Cochlearia borzaeana* | 0.0192 | 0 | 0 |
|  | *Megacarpaea polyandra* | 0.0192 | 0 | 0 |
|  | *Biscutella laevigata* | 0.0389 | 0 | 0 |
|  | *Iberis amara* | 0.0389 | 0 | 0 |
| ***petD*** | *Farsetia occidentalis* | 0.0085 | 0 | 0 |
|  | *Lobularia libyca* | 0.0345 | 0 | 0 |
|  | *Notoceras bicorne* | 0.0434 | 0 | 0 |
|  | *Parolinia ornata* | 0.0257 | 0 | 0 |
|  | *Morettia canescens* | 0.0523 | 0 | 0 |
|  | *Cochlearia borzaeana* | 0.0616 | 0.0028 | 0.045454545 |
|  | *Megacarpaea polyandra* | 0.0618 | 0.0028 | 0.045307443 |
|  | *Biscutella laevigata* | 0.0614 | 0 | 0 |
|  | *Iberis amara* | 0.0523 | 0 | 0 |
| ***petG*** | *Farsetia occidentalis* | 0 | 0 | 0 |
|  | *Lobularia libyca* | 0.0775 | 0 | 0 |
|  | *Notoceras bicorne* | 0.0373 | 0 | 0 |
|  | *Parolinia ornata* | 0.0373 | 0 | 0 |
|  | *Morettia canescens* | 0.0373 | 0 | 0 |
|  | *Cochlearia borzaeana* | 0.0373 | 0 | 0 |
|  | *Megacarpaea polyandra* | 0.0373 | 0 | 0 |
|  | *Biscutella laevigata* | 0.0373 | 0 | 0 |
|  | *Iberis amara* | 0.0373 | 0 | 0 |
| ***petL*** | *Farsetia occidentalis* | 0 | 0 | 0 |
|  | *Lobularia libyca* | 0 | 0 | 0 |
|  | *Notoceras bicorne* | 0 | 0 | 0 |
|  | *Parolinia ornata* | 0 | 0 | 0 |
|  | *Morettia canescens* | 0 | 0 | 0 |
|  | *Cochlearia borzaeana* | 0.0817 | 0 | 0 |
|  | *Megacarpaea polyandra* | 0 | 0 | 0 |
|  | *Biscutella laevigata* | 0 | 0 | 0 |
|  | *Iberis amara* | 0.0397 | 0 | 0 |
| ***petN*** | *Farsetia occidentalis* | 0 | 0 | 0 |
|  | *Lobularia libyca* | 0 | 0 | 0 |
|  | *Notoceras bicorne* | 0.1027 | 0 | 0 |
|  | *Parolinia ornata* | 0 | 0 | 0 |
|  | *Morettia canescens* | 0 | 0 | 0 |
|  | *Cochlearia borzaeana* | 0 | 0 | 0 |
|  | *Megacarpaea polyandra* | 0 | 0 | 0 |
|  | *Biscutella laevigata* | 0 | 0.0153 | 0 |
|  | *Iberis amara* | 0.0496 | 0 | 0 |
| ***psaA*** | *Farsetia occidentalis* | 0 | 0.0006 | 0 |
|  | *Lobularia libyca* | 0.0271 | 0.0006 | 0.022140221 |
|  | *Notoceras bicorne* | 0.0271 | 0 | 0 |
|  | *Parolinia ornata* | 0.0115 | 0 | 0 |
|  | *Morettia canescens* | 0.0212 | 0 | 0 |
|  | *Cochlearia borzaeana* | 0.045 | 0 | 0 |
|  | *Megacarpaea polyandra* | 0.0192 | 0 | 0 |
|  | *Biscutella laevigata* | 0.051 | 0.0017 | 0.033333333 |
|  | *Iberis amara* | 0.027 | 0 | 0 |
| ***psaB*** | *Farsetia occidentalis* | 0.006 | 0 | 0 |
|  | *Lobularia libyca* | 0.0324 | 0.0006 | 0.018518519 |
|  | *Notoceras bicorne* | 0.0449 | 0 | 0 |
|  | *Parolinia ornata* | 0.0262 | 0 | 0 |
|  | *Morettia canescens* | 0.0283 | 0 | 0 |
|  | *Cochlearia borzaeana* | 0.062 | 0.0018 | 0.029032258 |
|  | *Megacarpaea polyandra* | 0.0387 | 0 | 0 |
|  | *Biscutella laevigata* | 0.0449 | 0.0006 | 0.013363029 |
|  | *Iberis amara* | 0.0512 | 0.0012 | 0.0234375 |
| ***psaC*** | *Farsetia occidentalis* | 0 | 0 | 0 |
|  | *Lobularia libyca* | 0.0542 | 0 | 0 |
|  | *Notoceras bicorne* | 0.0176 | 0 | 0 |
|  | *Parolinia ornata* | 0.0357 | 0 | 0 |
|  | *Morettia canescens* | 0.0176 | 0 | 0 |
|  | *Cochlearia borzaeana* | 0.0927 | 0 | 0 |
|  | *Megacarpaea polyandra* | 0.0357 | 0 | 0 |
|  | *Biscutella laevigata* | 0.0543 | 0 | 0 |
|  | *Iberis amara* | 0.0176 | 0 | 0 |
| ***psaI*** | *Farsetia occidentalis* | 0 | 0 | 0 |
|  | *Lobularia libyca* | 0.0406 | 0 | 0 |
|  | *Notoceras bicorne* | 0 | 0 | 0 |
|  | *Parolinia ornata* | 0 | 0 | 0 |
|  | *Morettia canescens* | 0 | 0 | 0 |
|  | *Cochlearia borzaeana* | 0.0408 | 0.0117 | 0.286764706 |
|  | *Megacarpaea polyandra* | 0.04 | 0.0118 | 0.295 |
|  | *Biscutella laevigata* | 0 | 0 | 0 |
|  | *Iberis amara* | 0 | 0 | 0 |
| ***psaJ*** | *Farsetia occidentalis* | 0.0311 | 0 | 0 |
|  | *Lobularia libyca* | 0 | 0 | 0 |
|  | *Notoceras bicorne* | 0.0308 | 0 | 0 |
|  | *Parolinia ornata* | 0.0632 | 0 | 0 |
|  | *Morettia canescens* | 0.0309 | 0 | 0 |
|  | *Cochlearia borzaeana* | 0 | 0 | 0 |
|  | *Megacarpaea polyandra* | 0.0308 | 0 | 0 |
|  | *Biscutella laevigata* | 0 | 0 | 0 |
|  | *Iberis amara* | 0 | 0 | 0 |
| ***psbA*** | *Farsetia occidentalis* | 0.0121 | 0 | 0 |
|  | *Lobularia libyca* | 0.0495 | 0 | 0 |
|  | *Notoceras bicorne* | 0.0453 | 0 | 0 |
|  | *Parolinia ornata* | 0.0285 | 0 | 0 |
|  | *Morettia canescens* | 0.041 | 0 | 0 |
|  | *Cochlearia borzaeana* | 0.0801 | 0.0025 | 0.031210986 |
|  | *Megacarpaea polyandra* | 0.0538 | 0.0012 | 0.022304833 |
|  | *Biscutella laevigata* | 0.0756 | 0.0025 | 0.033068783 |
|  | *Iberis amara* | 0.058 | 0.0025 | 0.043103448 |
| ***psbB*** | *Farsetia occidentalis* | 0.0082 | 0 | 0 |
|  | *Lobularia libyca* | 0.0393 | 0.0035 | 0.089058524 |
|  | *Notoceras bicorne* | 0.0479 | 0.0009 | 0.018789144 |
|  | *Parolinia ornata* | 0.0166 | 0.0009 | 0.054216867 |
|  | *Morettia canescens* | 0.0222 | 0.0009 | 0.040540541 |
|  | *Cochlearia borzaeana* | 0.0598 | 0.0009 | 0.015050167 |
|  | *Megacarpaea polyandra* | 0.0337 | 0.0017 | 0.050445104 |
|  | *Biscutella laevigata* | 0.0656 | 0.0017 | 0.025914634 |
|  | *Iberis amara* | 0.0335 | 0.0026 | 0.07761194 |
| ***psbC*** | *Farsetia occidentalis* | 0.003 | 0 | 0 |
|  | *Lobularia libyca* | 0.0522 | 0 | 0 |
|  | *Notoceras bicorne* | 0.0522 | 0 | 0 |
|  | *Parolinia ornata* | 0.0241 | 0 | 0 |
|  | *Morettia canescens* | 0.0276 | 0.0029 | 0.105072464 |
|  | *Cochlearia borzaeana* | 0.0459 | 0 | 0 |
|  | *Megacarpaea polyandra* | 0.0396 | 0 | 0 |
|  | *Biscutella laevigata* | 0.0782 | 0 | 0 |
|  | *Iberis amara* | 0.0586 | 0 | 0 |
| ***psbD*** | *Farsetia occidentalis* | 0.0081 | 0 | 0 |
|  | *Lobularia libyca* | 0.0245 | 0 | 0 |
|  | *Notoceras bicorne* | 0.0628 | 0 | 0 |
|  | *Parolinia ornata* | 0.0121 | 0 | 0 |
|  | *Morettia canescens* | 0.004 | 0 | 0 |
|  | *Cochlearia borzaeana* | 0.0457 | 0 | 0 |
|  | *Megacarpaea polyandra* | 0.0122 | 0.0012 | 0.098360656 |
|  | *Biscutella laevigata* | 0.0329 | 0.0012 | 0.036474164 |
|  | *Iberis amara* | 0.0245 | 0 | 0 |
| ***psbE*** | *Farsetia occidentalis* | 0 | 0 | 0 |
|  | *Lobularia libyca* | 0.0352 | 0 | 0 |
|  | *Notoceras bicorne* | 0.0174 | 0 | 0 |
|  | *Parolinia ornata* | 0 | 0 | 0 |
|  | *Morettia canescens* | 0.0174 | 0 | 0 |
|  | *Cochlearia borzaeana* | 0.0721 | 0 | 0 |
|  | *Megacarpaea polyandra* | 0.0174 | 0 | 0 |
|  | *Biscutella laevigata* | 0.0534 | 0.0053 | 0.099250936 |
|  | *Iberis amara* | 0.0174 | 0 | 0 |
| ***psbF*** | *Farsetia occidentalis* | 0 | 0 | 0 |
|  | *Lobularia libyca* | 0.0343 | 0 | 0 |
|  | *Notoceras bicorne* | 0.0343 | 0 | 0 |
|  | *Parolinia ornata* | 0.0343 | 0 | 0 |
|  | *Morettia canescens* | 0.0343 | 0 | 0 |
|  | *Cochlearia borzaeana* | 0.0702 | 0 | 0 |
|  | *Megacarpaea polyandra* | 0.108 | 0 | 0 |
|  | *Biscutella laevigata* | 0.0702 | 0 | 0 |
|  | *Iberis amara* | 0.0343 | 0 | 0 |
| ***psbH*** | *Farsetia occidentalis* | 0.0178 | 0 | 0 |
|  | *Lobularia libyca* | 0.0742 | 0.0124 | 0.167115903 |
|  | *Notoceras bicorne* | 0.094 | 0.0062 | 0.065957447 |
|  | *Parolinia ornata* | 0.055 | 0.0062 | 0.112727273 |
|  | *Morettia canescens* | 0.0742 | 0.0062 | 0.083557951 |
|  | *Cochlearia borzaeana* | 0.0742 | 0.0124 | 0.167115903 |
|  | *Megacarpaea polyandra* | 0.055 | 0.0062 | 0.112727273 |
|  | *Biscutella laevigata* | 0.0738 | 0.0062 | 0.08401084 |
|  | *Iberis amara* | 0.1352 | 0.025 | 0.184911243 |
| ***psbI*** | *Farsetia occidentalis* | 0 | 0 | 0 |
|  | *Lobularia libyca* | 0.0385 | 0 | 0 |
|  | *Notoceras bicorne* | 0.079 | 0 | 0 |
|  | *Parolinia ornata* | 0.0385 | 0 | 0 |
|  | *Morettia canescens* | 0.0385 | 0 | 0 |
|  | *Cochlearia borzaeana* | 0.079 | 0 | 0 |
|  | *Megacarpaea polyandra* | 0.0801 | 0 | 0 |
|  | *Biscutella laevigata* | 0.0385 | 0 | 0 |
|  | *Iberis amara* | 0.0795 | 0.0124 | 0.155974843 |
| ***psbJ*** | *Farsetia occidentalis* | 0 | 0 | 0 |
|  | *Lobularia libyca* | 0 | 0.0116 | 0 |
|  | *Notoceras bicorne* | 0 | 0 | 0 |
|  | *Parolinia ornata* | 0 | 0 | 0 |
|  | *Morettia canescens* | 0.0306 | 0 | 0 |
|  | *Cochlearia borzaeana* | 0 | 0 | 0 |
|  | *Megacarpaea polyandra* | 0 | 0.0116 | 0 |
|  | *Biscutella laevigata* | 0.0306 | 0 | 0 |
|  | *Iberis amara* | 0 | 0 | 0 |
| ***psbK*** | *Farsetia occidentalis* | 0 | 0 | 0 |
|  | *Lobularia libyca* | 0.076 | 0 | 0 |
|  | *Notoceras bicorne* | 0.0752 | 0.0143 | 0.190159574 |
|  | *Parolinia ornata* | 0.0498 | 0 | 0 |
|  | *Morettia canescens* | 0.0755 | 0.0071 | 0.094039735 |
|  | *Cochlearia borzaeana* | 0.0498 | 0.0288 | 0.578313253 |
|  | *Megacarpaea polyandra* | 0.076 | 0 | 0 |
|  | *Biscutella laevigata* | 0.0495 | 0.0071 | 0.143434343 |
|  | *Iberis amara* | 0.076 | 0.0071 | 0.093421053 |
| ***psbL*** | *Farsetia occidentalis* | 0 | 0 | 0 |
|  | *Lobularia libyca* | 0.0423 | 0 | 0 |
|  | *Notoceras bicorne* | 0.0423 | 0 | 0 |
|  | *Parolinia ornata* | 0.0423 | 0 | 0 |
|  | *Morettia canescens* | 0.0423 | 0 | 0 |
|  | *Cochlearia borzaeana* | 0.0871 | 0.0112 | 0.12858783 |
|  | *Megacarpaea polyandra* | 0.0423 | 0 | 0 |
|  | *Biscutella laevigata* | 0 | 0 | 0 |
|  | *Iberis amara* | 0.0423 | 0.0112 | 0.264775414 |
| ***psbM*** | *Farsetia occidentalis* | 0 | 0 | 0 |
|  | *Lobularia libyca* | 0 | 0 | 0 |
|  | *Notoceras bicorne* | 0 | 0 | 0 |
|  | *Parolinia ornata* | 0 | 0 | 0 |
|  | *Morettia canescens* | 0.0423 | 0 | 0 |
|  | *Cochlearia borzaeana* | 0.0423 | 0 | 0 |
|  | *Megacarpaea polyandra* | 0.1347 | 0 | 0 |
|  | *Biscutella laevigata* | 0.0871 | 0 | 0 |
|  | *Iberis amara* | 0 | 0.0261 | 0 |
| ***psbN*** | *Farsetia occidentalis* | 0 | 0 | 0 |
|  | *Lobularia libyca* | 0 | 0 | 0 |
|  | *Notoceras bicorne* | 0 | 0 | 0 |
|  | *Parolinia ornata* | 0 | 0 | 0 |
|  | *Morettia canescens* | 0.0314 | 0 | 0 |
|  | *Cochlearia borzaeana* | 0.0314 | 0 | 0 |
|  | *Megacarpaea polyandra* | 0 | 0 | 0 |
|  | *Biscutella laevigata* | 0 | 0 | 0 |
|  | *Iberis amara* | 0 | 0 | 0 |
| ***psbT*** | *Farsetia occidentalis* | 0 | 0 | 0 |
|  | *Lobularia libyca* | 0.0435 | 0.0134 | 0.308045977 |
|  | *Notoceras bicorne* | 0 | 0 | 0 |
|  | *Parolinia ornata* | 0 | 0 | 0 |
|  | *Morettia canescens* | 0 | 0 | 0 |
|  | *Cochlearia borzaeana* | 0.0441 | 0 | 0 |
|  | *Megacarpaea polyandra* | 0 | 0 | 0 |
|  | *Biscutella laevigata* | 0 | 0 | 0 |
|  | *Iberis amara* | 0 | 0 | 0 |
| ***psbZ*** | *Farsetia occidentalis* | 0 | 0 | 0 |
|  | *Lobularia libyca* | 0 | 0 | 0 |
|  | *Notoceras bicorne* | 0.0451 | 0 | 0 |
|  | *Parolinia ornata* | 0.0222 | 0 | 0 |
|  | *Morettia canescens* | 0 | 0 | 0 |
|  | *Cochlearia borzaeana* | 0.0453 | 0.0072 | 0.158940397 |
|  | *Megacarpaea polyandra* | 0.0451 | 0.0144 | 0.319290466 |
|  | *Biscutella laevigata* | 0.1433 | 0 | 0 |
|  | *Iberis amara* | 0.0928 | 0 | 0 |
| ***rbcL*** | *Farsetia occidentalis* | 0.0115 | 0.0028 | 0.243478261 |
|  | *Lobularia libyca* | 0.0174 | 0.0055 | 0.316091954 |
|  | *Notoceras bicorne* | 0.0503 | 0.0101 | 0.200795229 |
|  | *Parolinia ornata* | 0.0115 | 0.0046 | 0.4 |
|  | *Morettia canescens* | 0.0203 | 0.0046 | 0.226600985 |
|  | *Cochlearia borzaeana* | 0.0487 | 0.0079 | 0.162217659 |
|  | *Megacarpaea polyandra* | 0.0427 | 0.0041 | 0.096018735 |
|  | *Biscutella laevigata* | 0.0381 | 0.0055 | 0.144356955 |
|  | *Iberis amara* | 0.0626 | 0.0055 | 0.087859425 |
| ***rpl2*** | *Farsetia occidentalis* | 0 | 0.0016 | 0 |
|  | *Lobularia libyca* | 0.0098 | 0 | 0 |
|  | *Notoceras bicorne* | 0.0049 | 0 | 0 |
|  | *Parolinia ornata* | 0.0049 | 0.0016 | 0.326530612 |
|  | *Morettia canescens* | 0 | 0 | 0 |
|  | *Cochlearia borzaeana* | 0.0098 | 0.0033 | 0.336734694 |
|  | *Megacarpaea polyandra* | 0.0098 | 0.0016 | 0.163265306 |
|  | *Biscutella laevigata* | 0.0147 | 0.0049 | 0.333333333 |
|  | *Iberis amara* | 0.0098 | 0.0033 | 0.336734694 |
| ***rpl14*** | *Farsetia occidentalis* | 0.0111 | 0.0036 | 0.324324324 |
|  | *Lobularia libyca* | 0.0223 | 0.0036 | 0.161434978 |
|  | *Notoceras bicorne* | 0.0111 | 0 | 0 |
|  | *Parolinia ornata* | 0.0111 | 0.0036 | 0.324324324 |
|  | *Morettia canescens* | 0.0338 | 0 | 0 |
|  | *Cochlearia borzaeana* | 0.0457 | 0.0036 | 0.078774617 |
|  | *Megacarpaea polyandra* | 0.0224 | 0 | 0 |
|  | *Biscutella laevigata* | 0.0458 | 0.0036 | 0.07860262 |
|  | *Iberis amara* | 0.0455 | 0 | 0 |
| ***rpl16*** | *Farsetia occidentalis* | 0.0099 | 0 | 0 |
|  | *Lobularia libyca* | 0.0609 | 0 | 0 |
|  | *Notoceras bicorne* | 0.0832 | 0.0066 | 0.079326923 |
|  | *Parolinia ornata* | 0.0506 | 0 | 0 |
|  | *Morettia canescens* | 0.0611 | 0 | 0 |
|  | *Cochlearia borzaeana* | 0.1164 | 0.0033 | 0.028350515 |
|  | *Megacarpaea polyandra* | 0.0505 | 0.0033 | 0.065346535 |
|  | *Biscutella laevigata* | 0.0937 | 0 | 0 |
|  | *Iberis amara* | 0.072 | 0.0033 | 0.045833333 |
| ***rpl20*** | *Farsetia occidentalis* | 0.0117 | 0 | 0 |
|  | *Lobularia libyca* | 0.0116 | 0.0192 | 1.655172414 |
|  | *Notoceras bicorne* | 0.0117 | 0.0076 | 0.64957265 |
|  | *Parolinia ornata* | 0.0116 | 0.0038 | 0.327586207 |
|  | *Morettia canescens* | 0 | 0.0038 | 0 |
|  | *Cochlearia borzaeana* | 0.0356 | 0.0114 | 0.320224719 |
|  | *Megacarpaea polyandra* | 0.0358 | 0.0076 | 0.212290503 |
|  | *Biscutella laevigata* | 0.0484 | 0.0152 | 0.314049587 |
|  | *Iberis amara* | 0.0117 | 0.023 | 1.965811966 |
| ***rpl22*** | *Farsetia occidentalis* | 0.0093 | 0.0027 | 0.290322581 |
|  | *Lobularia libyca* | 0.0468 | 0.0296 | 0.632478632 |
|  | *Notoceras bicorne* | 0.0522 | 0.0286 | 0.54789272 |
|  | *Parolinia ornata* | 0.0426 | 0.0177 | 0.415492958 |
|  | *Morettia canescens* | 0.0477 | 0.0081 | 0.169811321 |
|  | *Cochlearia borzaeana* | 0.1133 | 0.0317 | 0.279788173 |
|  | *Megacarpaea polyandra* | 0.0625 | 0.0205 | 0.328 |
|  | *Biscutella laevigata* | 0.114 | 0.0288 | 0.252631579 |
|  | *Iberis amara* | 0.0987 | 0.0081 | 0.082066869 |
| ***rpl23*** | *Farsetia occidentalis* | 0 | 0 | 0 |
|  | *Lobularia libyca* | 0 | 0.0047 | 0 |
|  | *Notoceras bicorne* | 0 | 0.0047 | 0 |
|  | *Parolinia ornata* | 0 | 0.0047 | 0 |
|  | *Morettia canescens* | 0 | 0.0047 | 0 |
|  | *Cochlearia borzaeana* | 0.0157 | 0.0047 | 0.299363057 |
|  | *Megacarpaea polyandra* | 0 | 0.0141 | 0 |
|  | *Biscutella laevigata* | 0 | 0.0047 | 0 |
|  | *Iberis amara* | 0 | 0.0047 | 0 |
| ***rpl32*** | *Farsetia occidentalis* | 0.0619 | 0 | 0 |
|  | *Lobularia libyca* | 0.0303 | 0.0082 | 0.270627063 |
|  | *Notoceras bicorne* | 0.0959 | 0.0165 | 0.172054223 |
|  | *Parolinia ornata* | 0.0619 | 0 | 0 |
|  | *Morettia canescens* | 0.0303 | 0 | 0 |
|  | *Cochlearia borzaeana* | 0.0944 | 0.0327 | 0.346398305 |
|  | *Megacarpaea polyandra* | 0.0303 | 0 | 0 |
|  | *Biscutella laevigata* | 0.0949 | 0 | 0 |
|  | *Iberis amara* | 0.0949 | 0 | 0 |
| ***rpl33*** | *Farsetia occidentalis* | 0 | 0 | 0 |
|  | *Lobularia libyca* | 0.023 | 0 | 0 |
|  | *Notoceras bicorne* | 0.0706 | 0.0065 | 0.092067989 |
|  | *Parolinia ornata* | 0.023 | 0 | 0 |
|  | *Morettia canescens* | 0.023 | 0 | 0 |
|  | *Cochlearia borzaeana* | 0.0467 | 0.0265 | 0.56745182 |
|  | *Megacarpaea polyandra* | 0.0228 | 0.0198 | 0.868421053 |
|  | *Biscutella laevigata* | 0.0834 | 0.0299 | 0.358513189 |
|  | *Iberis amara* | 0.0348 | 0.0164 | 0.471264368 |
| ***rpl36*** | *Farsetia occidentalis* | 0 | 0 | 0 |
|  | *Lobularia libyca* | 0 | 0 | 0 |
|  | *Notoceras bicorne* | 0.0801 | 0 | 0 |
|  | *Parolinia ornata* | 0 | 0 | 0 |
|  | *Morettia canescens* | 0 | 0 | 0 |
|  | *Cochlearia borzaeana* | 0.039 | 0 | 0 |
|  | *Megacarpaea polyandra* | 0.0798 | 0 | 0 |
|  | *Biscutella laevigata* | 0.0801 | 0 | 0 |
|  | *Iberis amara* | 0.039 | 0 | 0 |
| ***rpoA*** | *Farsetia occidentalis* | 0.0141 | 0.0039 | 0.276595745 |
|  | *Lobularia libyca* | 0.0189 | 0.0078 | 0.412698413 |
|  | *Notoceras bicorne* | 0.0236 | 0.0105 | 0.444915254 |
|  | *Parolinia ornata* | 0.0188 | 0.0052 | 0.276595745 |
|  | *Morettia canescens* | 0.0188 | 0.0078 | 0.414893617 |
|  | *Cochlearia borzaeana* | 0.0381 | 0.0145 | 0.380577428 |
|  | *Megacarpaea polyandra* | 0.0236 | 0.0144 | 0.610169492 |
|  | *Biscutella laevigata* | 0.0624 | 0.0104 | 0.166666667 |
|  | *Iberis amara* | 0.0577 | 0.0145 | 0.251299827 |
| ***rpoB*** | *Farsetia occidentalis* | 0.004 | 0.0012 | 0.3 |
|  | *Lobularia libyca* | 0.048 | 0.0053 | 0.110416667 |
|  | *Notoceras bicorne* | 0.0403 | 0.0051 | 0.126550868 |
|  | *Parolinia ornata* | 0.0189 | 0.0028 | 0.148148148 |
|  | *Morettia canescens* | 0.0237 | 0.0026 | 0.109704641 |
|  | *Cochlearia borzaeana* | 0.0496 | 0.0053 | 0.106854839 |
|  | *Megacarpaea polyandra* | 0.0382 | 0.0053 | 0.138743455 |
|  | *Biscutella laevigata* | 0.0509 | 0.0041 | 0.080550098 |
|  | *Iberis amara* | 0.0452 | 0.0045 | 0.099557522 |
| ***rpoC1*** | *Farsetia occidentalis* | 0.0063 | 0 | 0 |
|  | *Lobularia libyca* | 0.059 | 0.0032 | 0.054237288 |
|  | *Notoceras bicorne* | 0.041 | 0.0026 | 0.063414634 |
|  | *Parolinia ornata* | 0.0235 | 0.0013 | 0.055319149 |
|  | *Morettia canescens* | 0.0367 | 0.0013 | 0.035422343 |
|  | *Cochlearia borzaeana* | 0.0613 | 0.0032 | 0.052202284 |
|  | *Megacarpaea polyandra* | 0.0322 | 0.0032 | 0.099378882 |
|  | *Biscutella laevigata* | 0.0567 | 0.0032 | 0.05643739 |
|  | *Iberis amara* | 0.0571 | 0.0052 | 0.091068301 |
| ***rpoC2*** | *Farsetia occidentalis* | 0.0084 | 0.0032 | 0.380952381 |
|  | *Lobularia libyca* | 0.0498 | 0.0104 | 0.208835341 |
|  | *Notoceras bicorne* | 0.0552 | 0.0117 | 0.211956522 |
|  | *Parolinia ornata* | 0.032 | 0.0073 | 0.228125 |
|  | *Morettia canescens* | 0.0309 | 0.0076 | 0.245954693 |
|  | *Cochlearia borzaeana* | 0.0794 | 0.0169 | 0.212846348 |
|  | *Megacarpaea polyandra* | 0.0441 | 0.0101 | 0.229024943 |
|  | *Biscutella laevigata* | 0.0597 | 0.0162 | 0.271356784 |
|  | *Iberis amara* | 0.0653 | 0.0165 | 0.252679939 |
| ***rps2*** | *Farsetia occidentalis* | 0 | 0 | 0 |
|  | *Lobularia libyca* | 0.0314 | 0.0092 | 0.292993631 |
|  | *Notoceras bicorne* | 0.0186 | 0.011 | 0.591397849 |
|  | *Parolinia ornata* | 0 | 0.0037 | 0 |
|  | *Morettia canescens* | 0.0124 | 0.0037 | 0.298387097 |
|  | *Cochlearia borzaeana* | 0.0578 | 0.0092 | 0.15916955 |
|  | *Megacarpaea polyandra* | 0.0188 | 0.0111 | 0.590425532 |
|  | *Biscutella laevigata* | 0.0315 | 0.0129 | 0.40952381 |
|  | *Iberis amara* | 0.0574 | 0.0148 | 0.257839721 |
| ***rps3*** | *Farsetia occidentalis* | 0.0141 | 0.002 | 0.141843972 |
|  | *Lobularia libyca* | 0.0964 | 0.0059 | 0.06120332 |
|  | *Notoceras bicorne* | 0.089 | 0.0039 | 0.043820225 |
|  | *Parolinia ornata* | 0.0431 | 0.0059 | 0.136890951 |
|  | *Morettia canescens* | 0.0581 | 0.0039 | 0.067125645 |
|  | *Cochlearia borzaeana* | 0.1129 | 0.0079 | 0.069973428 |
|  | *Megacarpaea polyandra* | 0.0736 | 0.0079 | 0.107336957 |
|  | *Biscutella laevigata* | 0.1374 | 0.0099 | 0.072052402 |
|  | *Iberis amara* | 0.0887 | 0.0059 | 0.066516347 |
| ***rps4*** | *Farsetia occidentalis* | 0 | 0 | 0 |
|  | *Lobularia libyca* | 0.0139 | 0.0044 | 0.316546763 |
|  | *Notoceras bicorne* | 0.0353 | 0.0044 | 0.124645892 |
|  | *Parolinia ornata* | 0.0069 | 0.0044 | 0.637681159 |
|  | *Morettia canescens* | 0.0069 | 0.0044 | 0.637681159 |
|  | *Cochlearia borzaeana* | 0.0572 | 0.0044 | 0.076923077 |
|  | *Megacarpaea polyandra* | 0.0499 | 0.011 | 0.220440882 |
|  | *Biscutella laevigata* | 0.0647 | 0.011 | 0.170015456 |
|  | *Iberis amara* | 0.0647 | 0.0066 | 0.102009274 |
| ***rps7*** | *Farsetia occidentalis* | 0 | 0 | 0 |
|  | *Lobularia libyca* | 0 | 0 | 0 |
|  | *Notoceras bicorne* | 0.0088 | 0 | 0 |
|  | *Parolinia ornata* | 0 | 0 | 0 |
|  | *Morettia canescens* | 0 | 0 | 0 |
|  | *Cochlearia borzaeana* | 0 | 0 | 0 |
|  | *Megacarpaea polyandra* | 0 | 0 | 0 |
|  | *Biscutella laevigata* | 0.0088 | 0 | 0 |
|  | *Iberis amara* | 0 | 0 | 0 |
| ***rps8*** | *Farsetia occidentalis* | 0.0102 | 0.0066 | 0.647058824 |
|  | *Lobularia libyca* | 0.0419 | 0.0033 | 0.07875895 |
|  | *Notoceras bicorne* | 0.0418 | 0.0099 | 0.236842105 |
|  | *Parolinia ornata* | 0.0102 | 0 | 0 |
|  | *Morettia canescens* | 0.0205 | 0 | 0 |
|  | *Cochlearia borzaeana* | 0.1206 | 0.0033 | 0.027363184 |
|  | *Megacarpaea polyandra* | 0.0524 | 0.0201 | 0.383587786 |
|  | *Biscutella laevigata* | 0.0985 | 0.0099 | 0.100507614 |
|  | *Iberis amara* | 0.0748 | 0.0099 | 0.132352941 |
| ***rps11*** | *Farsetia occidentalis* | 0 | 0 | 0 |
|  | *Lobularia libyca* | 0.0281 | 0 | 0 |
|  | *Notoceras bicorne* | 0.028 | 0 | 0 |
|  | *Parolinia ornata* | 0.0187 | 0.0033 | 0.176470588 |
|  | *Morettia canescens* | 0.0281 | 0 | 0 |
|  | *Cochlearia borzaeana* | 0.0281 | 0 | 0 |
|  | *Megacarpaea polyandra* | 0.0282 | 0.0033 | 0.117021277 |
|  | *Biscutella laevigata* | 0.0376 | 0 | 0 |
|  | *Iberis amara* | 0.0473 | 0.0099 | 0.209302326 |
| ***rps12*** | *Farsetia occidentalis* | 0 | 0 | 0 |
|  | *Lobularia libyca* | 0.0102 | 0 | 0 |
|  | *Notoceras bicorne* | 0.0102 | 0 | 0 |
|  | *Parolinia ornata* | 0.0102 | 0 | 0 |
|  | *Morettia canescens* | 0.0102 | 0 | 0 |
|  | *Cochlearia borzaeana* | 0 | 0 | 0 |
|  | *Megacarpaea polyandra* | 0.0102 | 0 | 0 |
|  | *Biscutella laevigata* | 0.0102 | 0 | 0 |
|  | *Iberis amara* | 0.0205 | 0 | 0 |
| ***rps14*** | *Farsetia occidentalis* | 0 | 0.0043 | 0 |
|  | *Lobularia libyca* | 0.0146 | 0.0131 | 0.897260274 |
|  | *Notoceras bicorne* | 0.0146 | 0.0131 | 0.897260274 |
|  | *Parolinia ornata* | 0 | 0.0043 | 0 |
|  | *Morettia canescens* | 0 | 0.0043 | 0 |
|  | *Cochlearia borzaeana* | 0.0764 | 0.0131 | 0.171465969 |
|  | *Megacarpaea polyandra* | 0.0146 | 0.0043 | 0.294520548 |
|  | *Biscutella laevigata* | 0.0601 | 0.0043 | 0.071547421 |
|  | *Iberis amara* | 0.0445 | 0.0131 | 0.294382022 |
| ***rps15*** | *Farsetia occidentalis* | 0.0349 | 0.0049 | 0.140401146 |
|  | *Lobularia libyca* | 0.0905 | 0.0148 | 0.163535912 |
|  | *Notoceras bicorne* | 0.0703 | 0.0198 | 0.281650071 |
|  | *Parolinia ornata* | 0.0802 | 0.0123 | 0.153366584 |
|  | *Morettia canescens* | 0.0712 | 0.0098 | 0.137640449 |
|  | *Cochlearia borzaeana* | 0.1391 | 0.0173 | 0.124370956 |
|  | *Megacarpaea polyandra* | 0.1091 | 0.0098 | 0.089825848 |
|  | *Biscutella laevigata* | 0.1294 | 0.0148 | 0.114374034 |
|  | *Iberis amara* | 0.0898 | 0.0148 | 0.16481069 |
| ***rps16*** | *Farsetia occidentalis* | 0 | 0.005 | 0 |
|  | *Lobularia libyca* | 0 | 0 | 0 |
|  | *Notoceras bicorne* | 0.0855 | 0.0424 | 0.495906433 |
|  | *Parolinia ornata* | 0.0314 | 0.0101 | 0.321656051 |
|  | *Morettia canescens* | 0.0313 | 0.0204 | 0.651757188 |
|  | *Cochlearia borzaeana* | 0.063 | 0.0205 | 0.325396825 |
|  | *Megacarpaea polyandra* | 0.0331 | 0.0109 | 0.329305136 |
|  | *Biscutella laevigata* | 0.0514 | 0.0288 | 0.560311284 |
|  | *Iberis amara* | 0.0366 | 0.0584 | 1.595628415 |
| ***rps18*** | *Farsetia occidentalis* | 0 | 0 | 0 |
|  | *Lobularia libyca* | 0.0143 | 0.0043 | 0.300699301 |
|  | *Notoceras bicorne* | 0 | 0 | 0 |
|  | *Parolinia ornata* | 0 | 0 | 0 |
|  | *Morettia canescens* | 0.0291 | 0 | 0 |
|  | *Cochlearia borzaeana* | 0.0595 | 0 | 0 |
|  | *Megacarpaea polyandra* | 0.0144 | 0 | 0 |
|  | *Biscutella laevigata* | 0.0441 | 0 | 0 |
|  | *Iberis amara* | 0.0291 | 0.0043 | 0.147766323 |
| ***rps19*** | *Farsetia occidentalis* | 0 | 0 | 0 |
|  | *Lobularia libyca* | 0.0827 | 0 | 0 |
|  | *Notoceras bicorne* | 0.0656 | 0 | 0 |
|  | *Parolinia ornata* | 0.083 | 0 | 0 |
|  | *Morettia canescens* | 0.0487 | 0 | 0 |
|  | *Cochlearia borzaeana* | 0.1004 | 0.0143 | 0.142430279 |
|  | *Megacarpaea polyandra* | 0.0831 | 0.0095 | 0.114320096 |
|  | *Biscutella laevigata* | 0.1196 | 0.0095 | 0.079431438 |
|  | *Iberis amara* | 0.0842 | 0.0094 | 0.111638955 |
| ***ycf1*** | *Farsetia occidentalis* | 0.0065 | 0.0102 | 1.569230769 |
|  | *Lobularia libyca* | 0.0626 | 0.0546 | 0.872204473 |
|  | *Notoceras bicorne* | 0.0535 | 0.0523 | 0.977570093 |
|  | *Parolinia ornata* | 0.0322 | 0.033 | 1.02484472 |
|  | *Morettia canescens* | 0.0357 | 0.0306 | 0.857142857 |
|  | *Cochlearia borzaeana* | 0.0874 | 0.0608 | 0.695652174 |
|  | *Megacarpaea polyandra* | 0.0632 | 0.0512 | 0.810126582 |
|  | *Biscutella laevigata* | 0.088 | 0.0809 | 0.919318182 |
|  | *Iberis amara* | 0.0747 | 0.0755 | 1.010709505 |
| ***ycf2*** | *Farsetia occidentalis* | 0.0007 | 0.0006 | 0.857142857 |
|  | *Lobularia libyca* | 0.0067 | 0.0032 | 0.47761194 |
|  | *Notoceras bicorne* | 0.0095 | 0.0034 | 0.357894737 |
|  | *Parolinia ornata* | 0.0054 | 0.0024 | 0.444444444 |
|  | *Morettia canescens* | 0.008 | 0.003 | 0.375 |
|  | *Cochlearia borzaeana* | 0.0161 | 0.0081 | 0.50310559 |
|  | *Megacarpaea polyandra* | 0.0101 | 0.0047 | 0.465346535 |
|  | *Biscutella laevigata* | 0.0088 | 0.0055 | 0.625 |
|  | *Iberis amara* | 0.0081 | 0.0062 | 0.765432099 |
| ***ycf3*** | *Farsetia occidentalis* | 0 | 0 | 0 |
|  | *Lobularia libyca* | 0.0175 | 0 | 0 |
|  | *Notoceras bicorne* | 0.0362 | 0 | 0 |
|  | *Parolinia ornata* | 0.0119 | 0 | 0 |
|  | *Morettia canescens* | 0.0176 | 0.0026 | 0.147727273 |
|  | *Cochlearia borzaeana* | 0.0264 | 0 | 0 |
|  | *Megacarpaea polyandra* | 0 | 0 | 0 |
|  | *Biscutella laevigata* | 0.0362 | 0 | 0 |
|  | *Iberis amara* | 0 | 0 | 0 |
| ***ycf4*** | *Farsetia occidentalis* | 0.0159 | 0 | 0 |
|  | *Lobularia libyca* | 0.0159 | 0.0095 | 0.597484277 |
|  | *Notoceras bicorne* | 0.0239 | 0.0047 | 0.19665272 |
|  | *Parolinia ornata* | 0.0158 | 0.0047 | 0.297468354 |
|  | *Morettia canescens* | 0 | 0.0024 | 0 |
|  | *Cochlearia borzaeana* | 0.1013 | 0.0071 | 0.070088845 |
|  | *Megacarpaea polyandra* | 0.0403 | 0.0095 | 0.23573201 |
|  | *Biscutella laevigata* | 0.0324 | 0.0095 | 0.293209877 |
|  | *Iberis amara* | 0.0741 | 0.0047 | 0.0634278 |

**Supplementary table 11:** The detailed analysis of InDels, Average InDel Length, InDel Diversity K(i), InDel Diversity per site Pi(i) and alignment length in LSC, IR and SSC regions of *F. hamiltonii* by making pairwise alignment with nine species of Clade C of Brassicaceae I.e. *Farsetia occidentalis*, *Lobularia libyca, Notoceras bicorne, Parolinia ornata, Morettia canescens, Cochlearia borzaeana, Megacarpaea polyandra, Biscutella laevigata, and Iberis amara*.

| **Region** | Species (Pairwise alignment with ***Farsetia hamiltonii***) | **Alignment Length** | **No. of InDels** | **InDel Average Length** | **InDel Diversity k(i)** | **InDel Diversity per site Pi(i)** |
| --- | --- | --- | --- | --- | --- | --- |
| **Large Single Copy** | *Farsetia occidentalis* | 84346 | 806 | 6.2480 | 129 | 0.00153 |
|  | *Lobularia libyca* | 84763 | 3314 | 9.6900 | 342 | 0.00403 |
|  | *Notoceras bicorne* | 84865 | 3732 | 11.174 | 334 | 0.00394 |
|  | *Parolinia ornata* | 84926 | 2289 | 7.3840 | 310 | 0.00365 |
|  | *Morettia canescens* | 85587 | 3351 | 9.6570 | 347 | 0.00405 |
|  | *Cochlearia borzaeana* | 85388 | 3185 | 7.2060 | 442 | 0.00518 |
|  | *Megacarpaea polyandra* | 85486 | 3075 | 8.7110 | 353 | 0.00413 |
|  | *Biscutella laevigata* | 85664 | 3762 | 9.2430 | 407 | 0.00475 |
|  | *Iberis amara* | 85221 | 3113 | 8.1710 | 381 | 0.00447 |
| **Inverted Repeat** | *Farsetia occidentalis* | 26457 | 39 | 3.5450 | 11 | 0.00042 |
|  | *Lobularia libyca* | 26511 | 293 | 9.7670 | 30 | 0.00113 |
|  | *Notoceras bicorne* | 26507 | 194 | 5.8790 | 33 | 0.00124 |
|  | *Parolinia ornata* | 26484 | 119 | 4.5770 | 26 | 0.00098 |
|  | *Morettia canescens* | 26559 | 418 | 16.720 | 25 | 0.00094 |
|  | *Cochlearia borzaeana* | 26498 | 143 | 4.9310 | 29 | 0.00109 |
|  | *Megacarpaea polyandra* | 26575 | 263 | 8.7670 | 30 | 0.00113 |
|  | *Biscutella laevigata* | 26517 | 273 | 8.5310 | 32 | 0.00121 |
|  | *Iberis amara* | 26491 | 184 | 5.7500 | 32 | 0.00121 |
| **Small Single Copy** | *Farsetia occidentalis* | 18053 | 149 | 5.9600 | 25 | 0.00138 |
|  | *Lobularia libyca* | 18207 | 740 | 16.087 | 46 | 0.00253 |
|  | *Notoceras bicorne* | 18221 | 877 | 14.617 | 60 | 0.00329 |
|  | *Parolinia ornata* | 18132 | 1041 | 20.412 | 51 | 0.00281 |
|  | *Morettia canescens* | 18302 | 822 | 13.258 | 62 | 0.00339 |
|  | *Cochlearia borzaeana* | 18363 | 597 | 7.9600 | 75 | 0.00408 |
|  | *Megacarpaea polyandra* | 18365 | 650 | 8.904 | 73 | 0.00397 |
|  | *Biscutella laevigata* | 18394 | 857 | 10.202 | 84 | 0.00457 |
|  | *Iberis amara* | 18129 | 851 | 13.297 | 64 | 0.00353 |

**Supplementary table 12: S**ingle nucleotide polymorphisms (SNPs) in LSC, IR and SSC regions of *F. hamiltonii* by making pairwise alignment with nine species of Clade C of Brassicaceae I.e. *Farsetia occidentalis*, *Lobularia libyca, Notoceras bicorne, Parolinia ornata, Morettia canescens, Cochlearia borzaeana, Megacarpaea polyandra, Biscutella laevigata, and Iberis amara*.

| **Region** | **Species** | **Transition substituations** | | **Transversion substituaions** | | | |
| --- | --- | --- | --- | --- | --- | --- | --- |
|  |  | **A/G** | **C/T** | **A/T** | **A/C** | **C/G** | **G/T** |
| **Substitution Type** | ***Pairwise alignment with Farsetia hamiltonii*** | **R** | **Y** | **W** | **M** | **S** | **K** |
| Large Single Copy | *Farsetia occidentalis* | 76 | 66 | 49 | 80 | 8 | 76 |
|  | *Lobularia libyca* | 419 | 431 | 193 | 366 | 98 | 359 |
|  | *Notoceras bicorne* | 452 | 464 | 206 | 373 | 68 | 417 |
|  | *Parolinia ornata* | 310 | 270 | 135 | 242 | 35 | 229 |
|  | *Morettia canescens* | 366 | 336 | 177 | 281 | 50 | 254 |
|  | *Cochlearia borzaeana* | 739 | 761 | 348 | 605 | 155 | 589 |
|  | *Megacarpaea polyandra* | 511 | 485 | 207 | 346 | 65 | 336 |
|  | *Biscutella laevigata* | 651 | 655 | 304 | 444 | 129 | 463 |
|  | *Iberis amara* | 673 | 603 | 333 | 581 | 152 | 596 |
| Inverted Repeat | *Farsetia occidentalis* | 2 | 4 | 2 | 5 | 1 | 7 |
|  | *Lobularia libyca* | 34 | 27 | 10 | 21 | 14 | 25 |
|  | *Notoceras bicorne* | 38 | 36 | 8 | 28 | 12 | 28 |
|  | *Parolinia ornata* | 22 | 22 | 8 | 18 | 12 | 18 |
|  | *Morettia canescens* | 22 | 23 | 6 | 24 | 10 | 25 |
|  | *Cochlearia borzaeana* | 48 | 48 | 15 | 44 | 15 | 47 |
|  | *Megacarpaea polyandra* | 29 | 37 | 9 | 34 | 7 | 26 |
|  | *Biscutella laevigata* | 39 | 38 | 9 | 41 | 12 | 28 |
|  | *Iberis amara* | 42 | 33 | 14 | 31 | 11 | 37 |
| Small Single Copy | *Farsetia occidentalis* | 23 | 22 | 26 | 19 | 6 | 28 |
|  | *Lobularia libyca* | 134 | 139 | 73 | 112 | 42 | 148 |
|  | *Notoceras bicorne* | 125 | 135 | 73 | 121 | 30 | 135 |
|  | *Parolinia ornata* | 82 | 81 | 37 | 58 | 20 | 82 |
|  | *Morettia canescens* | 113 | 104 | 68 | 78 | 25 | 100 |
|  | *Cochlearia borzaeana* | 220 | 191 | 108 | 162 | 54 | 221 |
|  | *Megacarpaea polyandra* | 153 | 138 | 52 | 89 | 31 | 121 |
|  | *Biscutella laevigata* | 213 | 205 | 118 | 125 | 71 | 178 |
|  | *Iberis amara* | 186 | 198 | 84 | 148 | 61 | 187 |

**Supplementary table 13:** Nucleotide Diversity and Alignment Length in coding, non-coding, and intronic regions among *F. hamiltonii* and nine species of Clade C of Brassicaceae I.e. *Farsetia occidentalis*, *Lobularia libyca, Notoceras bicorne, Parolinia ornata, Morettia canescens, Cochlearia borzaeana, Megacarpaea polyandra, Biscutella laevigata, and Iberis amara*.

| **S.No** | ***Region*** | **Location** | **Nucleotide Diversity** | **Avg No. of Mutations** | **Region Length (all silent positions)** | **Alignment Length** |
| --- | --- | --- | --- | --- | --- | --- |
| 1 | *accD* | CDS | 0.03658 | 53 | 1437 | 1512 |
| 2 | *atpA* | CDS | 0.01807 | 28 | 1524 | 1524 |
| 3 | *atpB* | CDS | 0.01406 | 21 | 1473 | 1497 |
| 4 | *atpE* | CDS | 0.01069 | 4 | 399 | 399 |
| 5 | *atpF* | CDS | 0.02118 | 12 | 555 | 555 |
| 6 | *atpH* | CDS | 0.01707 | 4 | 246 | 246 |
| 7 | *atpI* | CDS | 0.01283 | 10 | 750 | 750 |
| 8 | *cemA* | CDS | 0.01771 | 12 | 690 | 690 |
| 9 | *ccsA* | CDS | 0.03061 | 30 | 984 | 1011 |
| 10 | *clpP* | CDS | 0.01267 | 7 | 591 | 591 |
| 11 | *matK* | CDS | 0.03593 | 54 | 1515 | 1612 |
| 12 | *ndhA* | CDS | 0.02298 | 25 | 1083 | 1086 |
| 13 | *ndhB* | CDS | 0.00277 | 3 | 1170 | 1539 |
| 14 | *ndhC* | CDS | 0.01518 | 6 | 363 | 363 |
| 15 | *ndhD* | CDS | 0.02069 | 31 | 1521 | 1524 |
| 16 | *ndhE* | CDS | 0.02055 | 6 | 306 | 306 |
| 17 | *ndhF* | CDS | 0.0245 | 55 | 2241 | 2247 |
| 18 | *ndhG* | CDS | 0.0221 | 12 | 531 | 531 |
| 19 | *ndhI* | CDS | 0.01574 | 8 | 483 | 504 |
| 20 | *ndhJ* | CDS | 0.01104 | 5 | 477 | 504 |
| 21 | *petA* | CDS | 0.01715 | 17 | 963 | 963 |
| 22 | *petB* | CDS | 0.0071 | 5 | 648 | 648 |
| 23 | *petD* | CDS | 0.01504 | 7 | 483 | 483 |
| 24 | *psaA* | CDS | 0.00861 | 19 | 2253 | 2253 |
| 25 | *psaB* | CDS | 0.00952 | 21 | 2205 | 2205 |
| 26 | *psaC* | CDS | 0.01084 | 3 | 246 | 246 |
| 27 | *psbA* | CDS | 0.01149 | 12 | 1062 | 1062 |
| 28 | *psbB* | CDS | 0.01209 | 18 | 1518 | 1527 |
| 29 | *psbC* | CDS | 0.01172 | 16 | 1371 | 1422 |
| 30 | *psbD* | CDS | 0.00908 | 10 | 1062 | 1062 |
| 31 | *psbE* | CDS | 0.00996 | 3 | 252 | 252 |
| 32 | *psbH* | CDS | 0.02513 | 6 | 222 | 222 |
| 33 | *rbcL* | CDS | 0.01284 | 18 | 1440 | 1452 |
| 34 | *rpl2* | CDS | 0.00488 | 4 | 825 | 828 |
| 35 | *rpl14* | CDS | 0.01319 | 5 | 369 | 369 |
| 36 | *rpl16* | CDS | 0.0207 | 8.4 | 408 | 408 |
| 37 | *rpl20* | CDS | 0.01758 | 6.2 | 354 | 354 |
| 38 | *rpl22* | CDS | 0.03505 | 16.9 | 483 | 504 |
| 39 | *rpl23* | CDS | 0.00339 | 1.0 | 282 | 282 |
| 40 | *rpl33* | CDS | 0.02576 | 5.2 | 201 | 201 |
| 41 | *rpoA* | CDS | 0.01626 | 16.0 | 984 | 1076 |
| 42 | *rpoB* | CDS | 0.01426 | 45.9 | 3219 | 3222 |
| 43 | *rpoC1* | CDS | 0.01397 | 28.3 | 2028 | 2043 |
| 44 | *rpoC2* | CDS | 0.02308 | 95.6 | 4140 | 4152 |
| 45 | *rps2* | CDS | 0.01657 | 11.8 | 711 | 726 |
| 46 | *rps3* | CDS | 0.02173 | 14.2 | 654 | 657 |
| 47 | *rps4* | CDS | 0.01511 | 9.2 | 606 | 606 |
| 48 | *rps7* | CDS | 0.00085 | 0.4 | 468 | 468 |
| 49 | *rps8* | CDS | 0.02606 | 10.6 | 405 | 411 |
| 50 | *rps11* | CDS | 0.01231 | 5.1 | 417 | 417 |
| 51 | *rps14* | CDS | 0.01452 | 4.4 | 303 | 303 |
| 52 | *rps15* | CDS | 0.02497 | 6.7 | 267 | 267 |
| 53 | *rps18* | CDS | 0.00879 | 2.7 | 306 | 306 |
| 54 | *rps19* | CDS | 0.02166 | 6.0 | 279 | 279 |
| 55 | *ycf1* | CDS | 0.06567 | 173.4 | 2641 | 5585 |
| 56 | *ycf2* | CDS | 0.00626 | 42.5 | 6792 | 6927 |
| 57 | *ycf3* | CDS | 0.00668 | 2.5 | 381 | 507 |
| 58 | *ycf4* | CDS | 0.0185 | 10.3 | 555 | 558 |
| 59 | *rps8-rpl14* | IGS | 0.08516 | 19.0 | 213 | 297 |
| 60 | *petN-psbM* | IGS | 0.0558 | 28.6 | 460 | 605 |
| 61 | *atpH-atpI* | IGS | 0.06249 | 30.3 | 445 | 571 |
| 62 | *psbK-psbI* | IGS | 0.07146 | 26.4 | 231 | 441 |
| 63 | *rps15-ycf1* | IGS | 0.08223 | 26.0 | 294 | 645 |
| 64 | *ycf4-cemA* | IGS | 0.06027 | 25.6 | 350 | 586 |
| 65 | *psaJ-rpl33* | IGS | 0.06666 | 27.8 | 382 | 501 |
| 66 | *petA-psbJ* | IGS | 0.06852 | 63.2 | 834 | 1177 |
| 67 | *rpl36-rps8* | IGS | 0.07028 | 32.8 | 441 | 554 |
| 68 | *rbcL-accD* | IGS | 0.04711 | 30.5 | 486 | 755 |
| 69 | *psaA-ycf3* | IGS | 0.05034 | 33.7 | 446 | 795 |
| 70 | *ndhF-rpl32* | IGS | 0.06798 | 45.9 | 543 | 1269 |
| 71 | *psbE-petL* | IGS | 0.05293 | 55.6 | 597 | 1499 |
| 72 | *rps18-rpl20* | IGS | 0.04238 | 12.2 | 275 | 313 |
| 73 | *atpF-atpH* | IGS | 0.04947 | 22.1 | 393 | 492 |
| 74 | *ndhG-ndhI* | IGS | 0.0783 | 24.9 | 281 | 458 |
| 75 | *ccsA-ndhD* | IGS | 0.07077 | 15.2 | 194 | 248 |
| 76 | *rps2-rpoC2* | IGS | 0.05199 | 11.2 | 210 | 224 |
| 77 | *psaI-ycf4* | IGS | 0.04643 | 19.3 | 410 | 491 |
| 78 | *atpI-rps2* | IGS | 0.059 | 13.8 | 217 | 275 |
| 79 | *psaC-ndhE* | IGS | 0.05455 | 13.0 | 230 | 272 |
| 80 | *clpP-psbB* | IGS | 0.03463 | 16.7 | 446 | 545 |
| 81 | *atpB-rbcL* | IGS | 0.03878 | 30.2 | 718 | 870 |
| 82 | *ndhB-rps7* | IGS | 0.01129 | 4.4 | 297 | 690 |
| 83 | *accD-psaI* | IGS | 0.03766 | 24.1 | 550 | 716 |
| 84 | *clpP intron2* | Intron | 0.04091 | 23.1 | 523 | 615 |
| 85 | *clpP intron1* | Intron | 0.03877 | 34.3 | 854 | 1039 |
| 86 | *rpl16 intron* | Intron | 0.05515 | 57.8 | 903 | 1228 |
| 87 | *ndhA intron* | Intron | 0.04837 | 51.5 | 1041 | 1185 |
| 88 | *petB intron* | Intron | 0.02933 | 23.0 | 771 | 816 |
| 89 | *atpF intron* | Intron | 0.03491 | 25.1 | 699 | 788 |
| 90 | *petD intron* | Intron | 0.02918 | 20.3 | 625 | 769 |
| 91 | *rpoC1 intron* | Intron | 0.03531 | 27.4 | 760 | 839 |
